# Promoters of ASCL1‐ and NEUROD1‐dependent genes are specific targets of lurbinectedin in SCLC cells

**DOI:** 10.15252/emmm.202114841

**Published:** 2022-03-09

**Authors:** Federico Costanzo, Marta Martínez Diez, Gema Santamaría Nuñez, Juan Ignacio Díaz‐Hernandéz, Carlos Mario Genes Robles, Javier Díez Pérez, Emmanuel Compe, Romeo Ricci, Tsai‐Kun Li, Frédéric Coin, Juan Fernando Martínez Leal, Eva Maria Garrido‐Martin, Jean Marc Egly

**Affiliations:** ^1^ Department of Functional Genomics and Cancer IGBMC, CNRS/INSERM/University of Strasbourg Equipe labellisée Ligue contre le Cancer Strasbourg France; ^2^ Cell Biology Department, Research and Development Pharmamar SA Colmenar Viejo Spain; ^3^ Laboratoire de Biochimie et de Biologie Moléculaire Nouvel Hôpital Civil Strasbourg France; ^4^ College of Medicine Center for Genomics and Precision Medicine National Taiwan University Taipei city Taiwan

**Keywords:** ASCL1/NEUROD1, E‐boxes/CpG islands, lurbinectedin, transcription addiction, Cancer, Chromatin, Transcription & Genomics, Respiratory System

## Abstract

Small‐Cell Lung Cancer (SCLC) is an aggressive neuroendocrine malignancy with a poor prognosis. Here, we focus on the neuroendocrine SCLC subtypes, SCLC‐A and SCLC‐N, whose transcription addiction was driven by ASCL1 and NEUROD1 transcription factors which target E‐box motifs to activate up to 40% of total genes, the promoters of which are maintained in a steadily open chromatin environment according to ATAC and H3K27Ac signatures. This leverage is used by the marine agent lurbinectedin, which preferentially targets the CpG islands located downstream of the transcription start site, thus arresting elongating RNAPII and promoting its degradation. This abrogates the expression of *ASCL1* and *NEUROD1* and of their dependent genes, such as *BCL2*, *INSM1*, *MYC*, and *AURKA*, which are responsible for relevant SCLC tumorigenic properties such as inhibition of apoptosis and cell survival, as well as for a part of its neuroendocrine features. In summary, we show how the transcription addiction of these cells becomes their Achilles’s heel, and how this is effectively exploited by lurbinectedin as a novel SCLC therapeutic endeavor.

## Graphical abstract

SCLCs poorly respond to current treatments, underlying the need for new therapeutic strategies. This study reports how the DNA binder lurbinectedin benefits from the SCLC cells transcription addiction to block the transcriptional program of key transcription factors involved in disease progression.

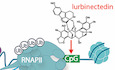

**The paper explained**

**Problem**

SCLC is an extremely aggressive cancer, characterized by rapid growth, early metastasis, and refractoriness to current therapies. In contrast to NSCLC, SCLC did not benefit from significant therapeutic advances over the last decades. We explored a new therapeutic strategy based on a better understanding of SCLC development, and particularly on its addiction to transcription.

**Results**

We found that the SCLC transcription addiction was mainly directed by activators, such as ASCL1 and NEUROD1, that continuously stimulated the expression of their target genes. These genes, constantly in an open chromatin environment, were susceptible to lurbinectedin, a marine alkaloid that specifically targets G‐rich DNA triplets in CpG island motifs located downstream of the gene transcription start site. Accordingly, lurbinectedin was used to block ASCL1‐ and NEUROD1‐dependent transcription programs, and thus trigger apoptotic programs in a panel of SCLC cell lines.

**Impact**

This study demonstrates how targeting specific areas of the genome results in a more effective and less harmful anti‐tumor effect. Our data clarify the mechanism of action of ASCL1 and NEUROD1, revealing their genomic targets in different genomic contexts, which represents a step forward for personalized medicine in SCLC patients.

## Introduction

Lung cancer is one of the most prevalent cancers worldwide, with 2.2 million people newly diagnosed every year, and 1.8 million deaths per year (Bray *et al*, 2018; Fitzmaurice *et al*, 2019; Sung *et al*, 2021). Among lung malignancies, small‐cell lung cancer (SCLC) represents around 15% of cases and, with over 250,000 new diagnoses per year, is considered the sixth most common cause of cancer death (Sabari *et al*, 2017), presenting a 5‐year survival rate below 5% (Augert & MacPherson, 2014).

Small‐cell lung cancer is an extraordinarily aggressive disease, with a very rapid growth, early metastasization, and acquisition of resistance to current therapies (Gazdar *et al*, 2017). In clear contrast with nonsmall‐cell lung cancer (NSCLC), where targeted therapies and immunotherapy have greatly improved patient survival, SCLC still has not witnessed significant therapeutic advances over the last decades. In particular, immunotherapy had only a modest impact in the first line of treatment in combination with classical etoposide and platinum‐containing regimes (Horn *et al*, 2018). A thorough molecular understanding of the genomic aberrations of NSCLC has facilitated the development of targeted therapies that improved patient survival (Zappa & Mousa, 2016). On the contrary, the lack of actionable targets in SCLC has precluded the development of better treatments.

Fortunately, recent progress has led to a better understanding of the molecular processes occurring in SCLC, highlighting transcriptional addiction as the main actionable target for patient treatment (Christensen *et al*, 2014; Kim *et al*, 2018). Although SCLC is usually characterized by the almost ubiquitous inactivation of both *TP53* and *RB1* tumor suppressor genes (Sato *et al*, 2007; Peifer *et al*, 2012; Rudin *et al*, 2019), the cell of origin is of paramount importance for the triggering of the disease, and other events dependent on additional transcriptional regulators are necessary and indispensable to drive cells toward tumorigenesis (Sutherland *et al*, 2011). Indeed, epigenetic and transcriptomic studies have revealed an unsuspected molecular diversity among SCLC tumors that can be divided into neuroendocrine and non‐neuroendocrine groups, both being subdivided into two subgroups that depend on the transcription factor driving the oncogenic process (Rudin *et al*, 2019). In the neuroendocrine group, SCLC‐A tumors are driven by overexpression of Achaete‐scute homolog 1 (*ASCL1*) while SCLC‐N tumors are driven by Neurogenic Differentiation 1 (*NEUROD1*) overexpression. Other SCLCs are driven by POU Class 2 Homeobox 3 (*POU2F3*) or Yes‐Associated Protein 1 (*YAP‐1*). As a consequence, downregulation of these transcription factors and/or of their responsive genes arose as a key target for SCLC therapy (Augustyn *et al*, 2014). Indeed, using a DNA binder and/or preventing their elimination (through DNA damage repair) might represent another area of development for SCLC treatments (Sen *et al*, 2018) which are not currently satisfactory (Poirier *et al*, 2020).

Here, we focused on the study of the transcriptional process underlying two of the major subtypes of SCLC, SCLC‐A and SCLC‐N, both with neuroendocrine features with which respective transcriptional dysregulation programs are carried out by ASCL1 and NEUROD1 (Rudin *et al*, 2019; Ireland *et al*, 2020). In cell lines belonging to these groups, we first checked the high expression of ASCL1 and NEUROD1 transcriptional factors, which are known to be required for the proper development of pulmonary neuroendocrine cells (Borges *et al*, 1997; Ito *et al*, 2000; Neptune *et al*, 2008), for carcinogenesis, and for the survival of a majority of lung cancer cells with neuroendocrine features (Jiang *et al*, 2009; Borromeo *et al*, 2016). These two transcription factors target their E‐box cognate sequence and regulate either uniquely or commonly, a large amount of their responsive genes. In line with the transcription addiction profile of the SCLC cells, we observed that the surroundings of the promoters (including the transcription start site, TSS) of these ASCL1‐ and NEUROD1‐responsive genes exhibited open and accessible chromatin structure that was fully exploitable for genotoxic attack by DNA binders. We then showed that lurbinectedin, a marine derived alkaloid which harbors a high specificity toward CGG‐rich triplets (Leal *et al*, 2010), promptly bound CpG‐rich regions (also named CpG islands) located downstream of the TSS of activated genes. Such binding primed the arrest of elongating RNA polymerase II (RNAPII) and its subsequent degradation, having as a consequence abrogation of specific genes, such as BCL2, INSM1, MYB, MYC family members (all involved in tumorigenesis and in neuroendocrine features of the disease), ultimately triggering SCLC cell death.

## Results

### DMS‐53 cells are characterized by a high transcriptional activity

**Figure 1:**
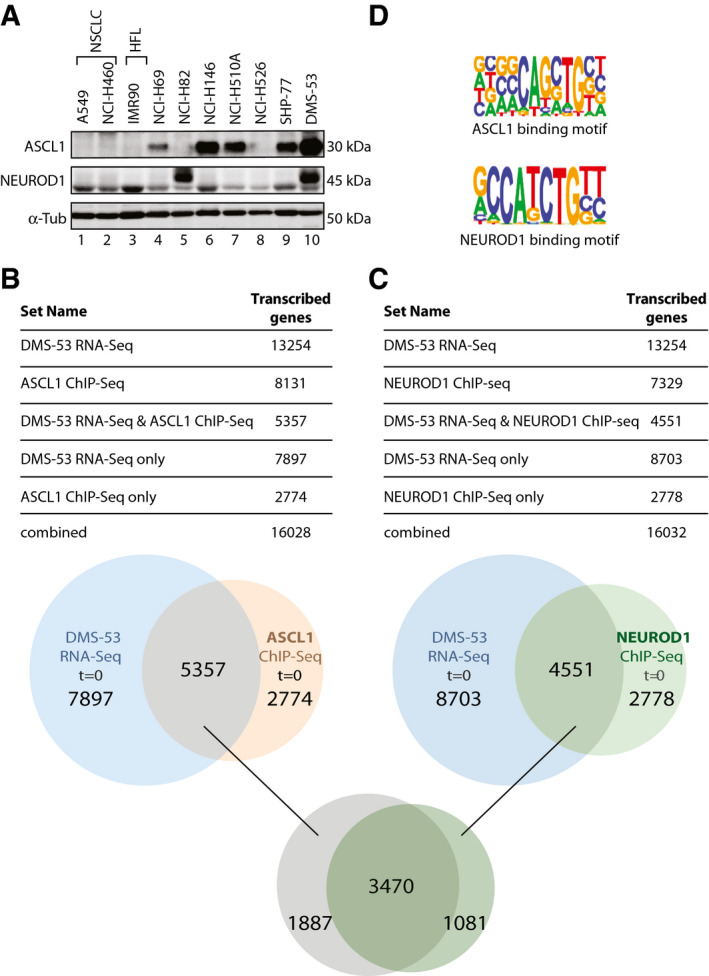
Overexpression of ASCL1 and NEUROD1 promotes the expression of their responsive genes AWestern blot showing the protein expression of ASCL1 and/or NEUROD1 in NCI‐H69, NCI‐H82, NCI‐H146, NCI‐H510A, NCI‐H526, SHP‐77, and DMS‐53 SCLC cells (lanes 4–10); A549 and NCI‐H460 NSCLC cells and a IMR‐90 HFL cell line (lanes 1–3). Tubulin (α‐Tub) is shown as loading control. Each blot is representative from at least three independent experiments.B, CVenn diagrams showing the overlap between DMS‐53 RNA‐seq (Blue circle) and either ASCL1 ChIP‐seq (Orange circle) (B) or NEUROD1 (Green circle) (C) ChIP‐seq promoter‐annotated peaks in untreated conditions (*t* = 0). Top panels. Tables showing the common and unique features from Venn diagram. According to RNA‐seq data, a gene has been considered transcribed, if it has a coverage (number of reads per base of the gene) of more than 1 and a cutoff value of TPM (transcript per million reads) of 4. DMS53 RNA‐seq: genes being transcribed; ASCL1‐ and NEUROD1‐ChIp‐seq: genes targeted byASCL1 or NEUROD1; DMS53 RNA‐seq and either ASCL1‐ or NEUROD1‐seq: genes bound by either ASCL1 or NEUROD1 and being transcribed; DMS‐RNA‐seq only: transcribed genes not targeted by ASCL1 or NEUROD1; ASCL1‐ and NEUROD1‐ChIp‐seq only: nontranscribed genes in our experimental conditions. Lower panel : Venn diagram showing the overlap between ASCL1‐ and NEUROD1 (Dark green Circle)‐targeted and downregulated genes (3,470 genes) in untreated conditions.DPicture of ASCL1 (above) and NEUROD1 (below) ChIP‐seq‐associated motifs around the genome (*P* = 1e‐6800 and *P* = 1e‐60, respectively). 13254 RNA‐seq‐filtered genes by mean coverage > 1 and mean tpm >= 4. 10657 had 1 or more ASCL1 motif in their custom promoter region. 8,925 genes had 1 or more NEUROD1 motifs in their custom promoter region. Source data are available online for this figure.

Transcription addiction in human‐derived SCLC cell lines has been attributed to overexpression of several DNA‐binding transcription factors. Among them ASCL1 and NEUROD1 are two known drivers of SCLC pathogenesis (Poirier *et al*, 2013; Borromeo *et al*, 2016; Rudin *et al*, 2019). We hence first sought to examine a panel of SCLC cell lines and classified them according to their expression profile of either ASCL1 (NCI‐H69, NCI‐H146, NCI‐H510A, and SHP‐77), NEUROD1 (NCI‐H82), or both (DMS‐53) (*n* = 3 biological replicates) (Fig 1A). Two NSCLC, A549 and NCI‐H460, and a Human Fetal Lung (HFL) IMR‐90 cell lines, which did not express ASCL1 or NEUROD1, were included. Among the different SCLC cell lines tested, we focused on DMS‐53 cells that overexpressed both ASCL1 and NEUROD1, and dissected how the gene expression pattern of this cell line depended on the action of these two DNA‐binding factors.

To define the transcription‐addiction profile of DMS‐53 cells, we performed chromatin immunoprecipitation followed by high‐throughput DNA sequencing (ChIP‐seq) on several regulatory components involved in active transcription. A quite large number of genes were targeted by either ASCL1 (8,131) or NEUROD1 (7,329) (Table 1). Moreover, analyses of ChIP‐seq and RNA‐seq data revealed that 40% (5,357) of genes being transcribed (among a total of 13,254) were targeted by ASCL1 (Fig 1B); similarly, 34% (4,551) of transcribed genes were targeted by NEUROD1 (Fig 1C).

**Table 1:** Number of Promoter‐TSS‐annotated Peaks from (hg19) ChIP‐seq upon untreated (*t* = 0 h) and untreated (*t* = 4 h) lurbinectedin DMS53 cells.

	ChIP‐seq	Untreated *t* = 0	Treated *t* = 4	Common genes (*t* = 0 and *t* = 4)	Genes bound by Lur (*t* = 4)
A	Bio‐Lur		7,963		
B	ASCL1	8,131	7,230	6,331 (87%)	4,037 (56%)
C	NEUROD1	7,329	7,214	4,741 (66%)	4,786 (66%)
D	RNAPII	10,030	10,448	7,873 (75%)	5,569 (57%)
E	H3K27Ac	8,835	9,836	7,317 (74%)	4,111 (53%)
F	ATAC	10,164	9,772	8,342 (85%)	4,056 (42%)
	E,F	6,181	6,101		
	B,D,E,F	4,089	3,494	2,793 (68%)	2,177 (62%)
	C,D,E,F	3,215	2,605	1,772 (55%)	1,873 (71%)

Letters corresponding to each ChIP‐seq dataset (Column 1) are shown on the left. Annotated Peaks for *t* = 0 (Column 2) and *t* = 4 (Column 3) are shown. Overlap between different ChIP‐seq datasets and values corresponding to respective lurbinectedin binding are shown (Columns 4 and 5).

ASCL1 and NEUROD1 transcription factors contain basic helix loop helix (bHLH) motifs that bind to CAGCTG and CATCTG sequences, respectively (Fig 1D), and two E‐box sequences identified by ChIP‐seq analysis (*P* = 1e‐6800 and *P* = 1e‐60, respectively). Intersection between ASCL1/NEUROD1 and RNA‐seq dataset revealed how 4,864 transcribed genes in ASCL1 ChIP‐seq (91% of the 5357) were containing an ASCL1 motif. Similarly, 3,573 transcribed genes in NEUROD1 ChIP‐seq (79% of the 4551) were containing a NEUROD1 motif. Given the similarity between both transcription factor‐binding motifs (the E‐box consensus is minimally defined as CANNTG), we sought to analyze a potential overlap in their regulatory function. Interestingly, we found that 3,470 genes (almost 26% of the 13,254 transcribed genes) were actively transcribed and targeted by ASCL1 and NEUROD1 (Fig 1B and C). It seems that in DMS53 cells, a certain number of genes were targeted by either ASCL1 (1,887) or NEUROD1 (1,081) in our experimental conditions.

**Figure 2:**
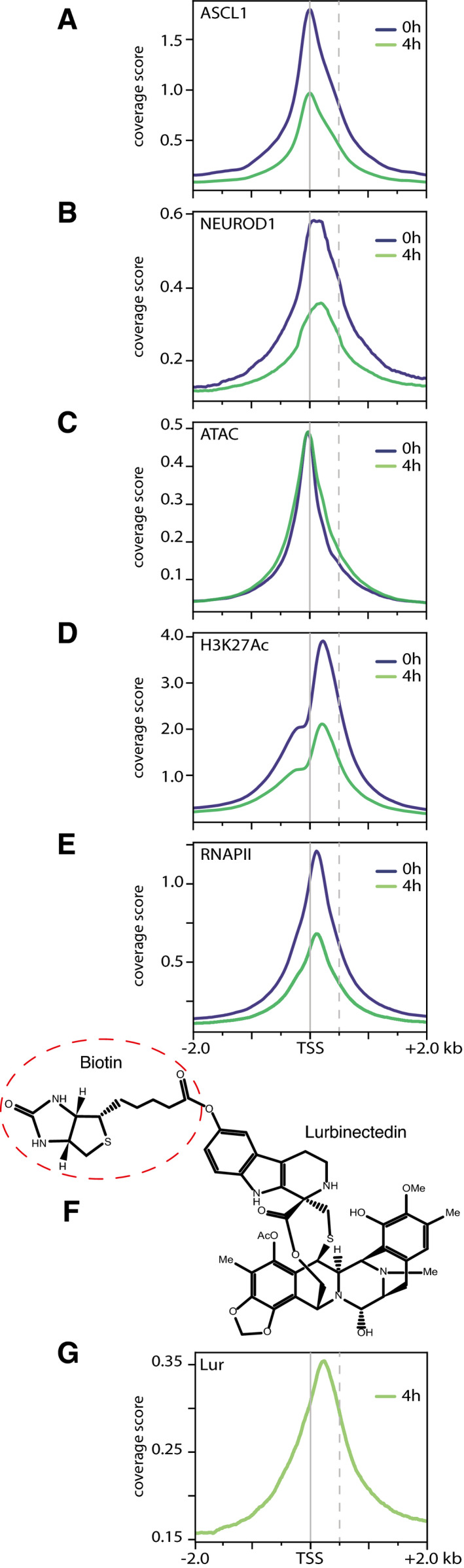
Promoter‐TSS occupancy of ASCL1‐ and NEUROD1‐targeted genes A–EChIP‐seq experiments indicate the localization of (A) ASCL1, (B) NEUROD1, and (E) RNAPII. ATAC‐seq (C) and (D) the presence of H3K27Ac mark indicate the chromatin accessibility around the TSS at *t* = 0 (blue) and *t* = 4 h (green) after lurbinectedin treatment; −2.0 and +2.0 kb to TSS coordinates of all hg19 genes.FStructure of Bio‐lur. The Biotin moiety is highlighted in dotted circle.GChem‐seq (Green) experiments indicate the localization of lurbinectedin around TSS. All ChIP‐seq profiles are representative of two independent experiments.

**Figure EV1:**
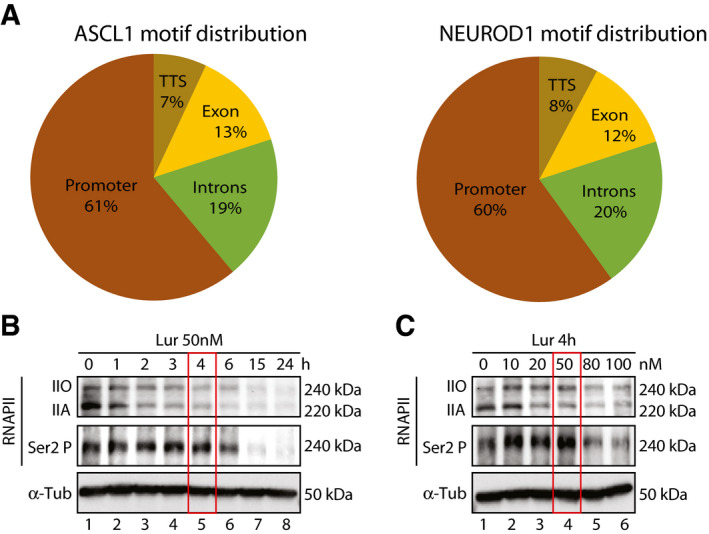
Distribution of ASCL1 and NEUROD1 cognate sequences and optimization of Lurbinectedin treatment Venn diagram showing the Genomic Distribution of ASCL1 and NEUROD1 motif (in percentage) on transcripts from RNA‐seq. ASCL1 and NEUROD1 motif occurrence has been calculated on custom promoter regions (−1 kb/+1 kb) on transcripts having at least 1 motif in their promoter region.Western blot showing time‐dependent hypo‐ (IIA) or hyper (IIO)‐phosphorylated RNAPII (upper panel) and phosphorylated RNAPII at Ser2 (Ser2‐P) (middle panel) protein level decrease in DMS‐53 cell lines from 0 h to 24 h after 50 nM or lurbinectedin treatment. Red box (lane 5) highlights the time‐point of RNAPII significant decrease. α‐Tubulin (α‐Tub) is shown as loading control. For all the experiments, cells were collected 4 h after 50 nM lurbinectedin treatment.Western blot showing dose dependency of RNAPIIA or RNAPIIO) (upper panel) and phosphorylated RNAPII at Ser2 (Ser2‐P) (lower Panel) protein level decrease in DMS‐53 cell lines at different lurbinectedin concentrations at 4 h. Red box (lane 4) highlights the optimal concentration for RNAPII significant decrease. α‐Tubulin (α‐Tub) is shown as loading control. For all the experiments, cells were collected 4 h after 50 nM lurbinectedin treatment. Source data are available online for this figure.

We then identified the genomic localization of both ASCL1 and NEUROD1 at the surroundings of the transcription start site (TSS) in the untreated conditions (*t* = 0) (Fig 2A and B, blue curves) and revealed how ASCL1‐ and NEUROD1‐binding motifs (around 60%) were overrepresented in the promoter‐TSS regions of our RNA‐seq transcript datasets (Fig EV1A). In particular, 13,283 RNA‐seq‐filtered genes by MEAN_COVERAGE > 1 and MEAN_TPM ≥ 4, 10,657 has 1 or more ASCL1 motif in their custom promoter region (Available link). Also 8,925 genes have 1 or more NEUROD1 motif in their custom promoter region.

Transcriptionally active regions are characterized by specific chromatin environment. To highlight the open chromatin accessibility landscape of DMS‐53 cells, we performed an Assay for Transposase‐Accessible Chromatin followed by sequencing (ATAC‐seq). Analysis of the peak distribution of ATAC signal identified 10,164 genes (Table 1) in an open (i.e., potentially active) chromatin state mainly surrounding the TSS (Fig 2C). In parallel, ChIP‐seq analysis showed that acetylated histone H3K27 (H3K27Ac), a mark of highly active transcription, was detected around the TSS (Fig 2D) of 8,835 genes (Table 1). Overlapping ATAC‐seq and ChIp‐seq‐H3K27Ac revealed 6,181 genes (approximately 47%) in an open chromatin, which might indicate an active transcription state. By using specific antibodies targeting the C‐terminal domain (CTD) of the largest subunit of RNAPII, we found RNAPII in the vicinity of TSS (Fig 2E) in up to 10,030 genes (Table 1). These results indicated that in untreated conditions (*t* = 0, when cells were collected), at least 4,089 ASCL1‐targeted genes were actively transcribed as revealed by the ATAC and the H3K27Ac signatures and the presence of elongating RNAPII. Similarly, 3,215 NEUROD1‐targeted genes were actively transcribed in untreated conditions (Table 1).

Altogether, our data show that most of ASCL1‐ and NEUROD1‐targeted genes are continuously transcriptionally active in DMS‐53 SCLC cells, and favor an open chromatin environment around the TSS.

### SCLC cells overexpressing ASCL1 and NEUROD1 are sensitive to lurbinectedin

We hence hypothesized that the open chromatin environment associated with the high transcriptional activity of A‐SCLC and N‐SCLC cells might represent a specific opportunity for a genotoxic attack by specific DNA binders such as lurbinectedin. This compound of marine origin, covalently binds to the central guanine of the triplets (through the hydroxyl of its hemiaminal group) and interacts with the opposite DNA strand (through hydrogen bond and Van der Waals interactions (Marco *et al*, 2006; Leal *et al*, 2010; Feuerhahn *et al*, 2011; Santamaria Nunez *et al*, 2016). DMS53 cells were exposed to 50 nM lurbinectedin for 4 h before being collected for ChIp‐seq and RNA‐seq and additional experiments (Fig EV1B and C; Material and Methods).

**Figure EV2:**
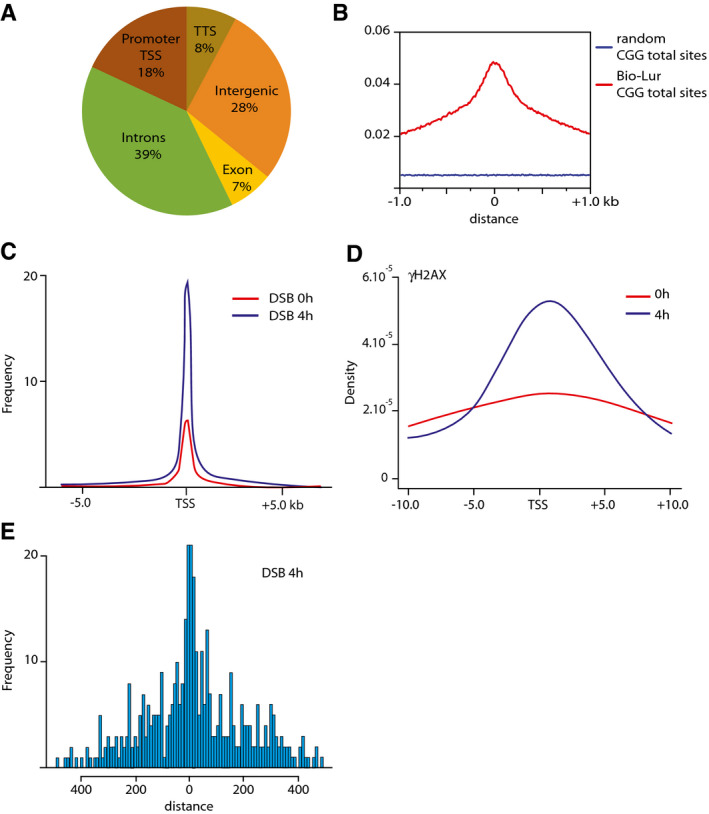
Lurbinectedin induces DNA breaks in its surrounding around gene TSS AVenn diagram showing the distribution of lurbinectedin‐annotated Peaks (in percentage) all over the genome (hg19). Peak annotation has been performed by customizing the promoter region from −1 kb up to +1 kb around TSS, in order to comprise Lur peaks downstream from TSS.BEnrichment of CGG motifs (red) into −/+1/2 kb around lurbinectedin Promoter‐located peaks and random regions of similar size (blue) from hg19 genome promoters as background reference and their presence in 10 bp were counted and scored.C, D(C) BrdUTP‐ChIP‐seq representing DNA breaks (DSB) and (D) ChIP‐seq experiments indicate γH2AX localization. Lines representing ChIP‐seq profile at 0 h (red) and 4 h (blue) after lurbinectedin treatment.EHistogram of the distance (in bp) between the DSBs (BrdUTP ChIP‐seq) and the TSS at 4 h after lurbinectedin treatment.

In the absence of corresponding antibodies, a biotinylated structural analog of lurbinectedin (Bio‐lur, PM120306) was synthesized to study its incorporation within SCLC cells and to identify its genomic binding sites by performing chemical affinity capture (Chem‐Seq, by using antibodies directed towards the biotin moiety) and subsequent sequencing. Bio‐lur resulted from the binding of biotin to the β‐carboline group of lurbinectedin (Fig 2F), and as the parent compound, targets central guanines in the DNA through the hydroxyl of its hemiaminal group. Upon 4 h of treatment of DMS53 cells with Bio‐lur (Santamaria Nunez *et al*, 2016), Chem‐seq experiments showed that certain genomic regions were preferentially targeted by the drug. Surprisingly, up to 18% of the drug bound promoter regions and more precisely downstream of the TSS, reflecting a high specificity of action (Figs 2G and EV2A). Moreover, sequencing analysis revealed that lurbinectedin preferentially bound CGG/GCC‐rich triplets (Fig EV2B). To further assess the efficacy of Bio‐lur, we incorporated Bromodeoxyuridine (BrdU) in DNA (Santamaria Nunez *et al*, 2016) and revealed, by BrdU immune‐precipitation and deep sequencing, the formation of DNA breaks surrounding the promoter area (Fig EV2C), that was further confirmed by the detection of phosphorylated histone H2AX (γH2AX), a hallmark of DNA breaks (Fig EV2D). Moreover, we found a close proximity between Bio‐lur and the DNA breaks, being distant of < 100 bp (Fig EV2E).

**Figure 3:**
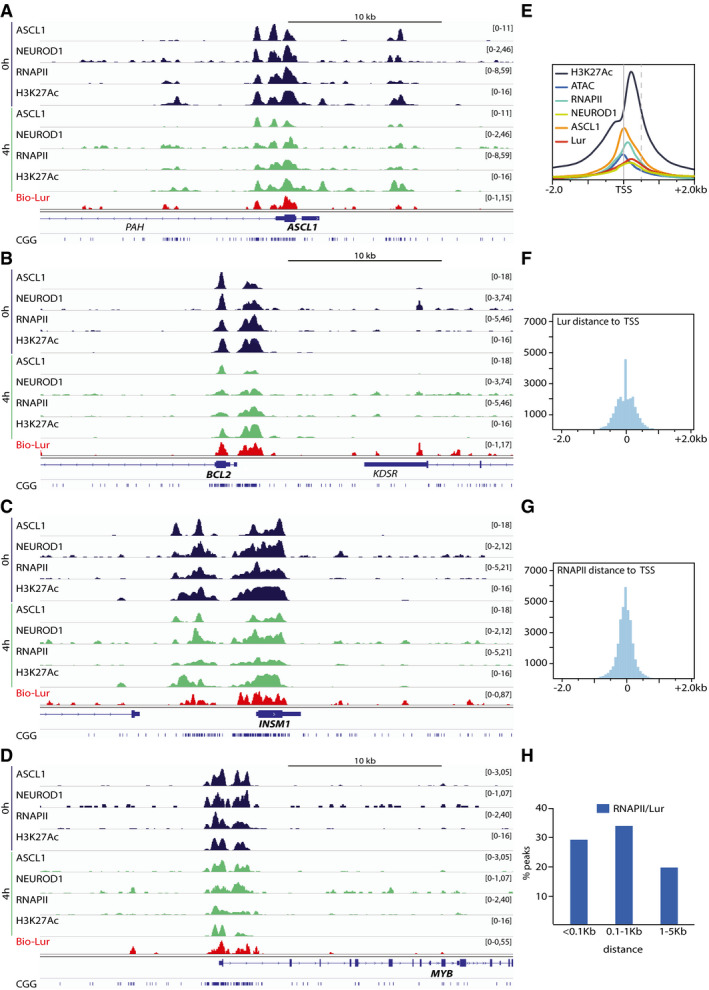
Lurbinectedin binds transcriptionally active regions in SCLC A–DChIp‐seq genome track of ASCL1/NEUROD1, RNAPII, and H3K27Ac on *ASCL1*, *BCL2; INSM1* and *MYB* (hg19), in untreated (dark blue) and in lurbinectedin‐treated (green) conditions, respectively; to be noticed, the location of CGG‐rich motifs (lower part of each panel) which parallel the presence of lurbinectedin (red).ESummary of the localization of lurbinectedin ASCL1‐, NEUROD1‐, RNAPII‐, H3K27Ac‐ChIP‐seq, ATAC‐seq, and lurbinectedin‐Chem‐seq.F, GHistogram showing the distance (in bp) between TSS and either (F) lurbinectedin or (G) RNAPII bound to the DNA. Only peaks located in region between −2.0 and +2.0 kb on each side of TSS were taken into consideration. The y axis on every ChIp‐seq profile represents the Coverage Score.HHistogram showing the absolute distance between RNAPII and lurbinectedin peaks from the nearest TSS. Source data are available online for this figure.

**Figure EV3:**
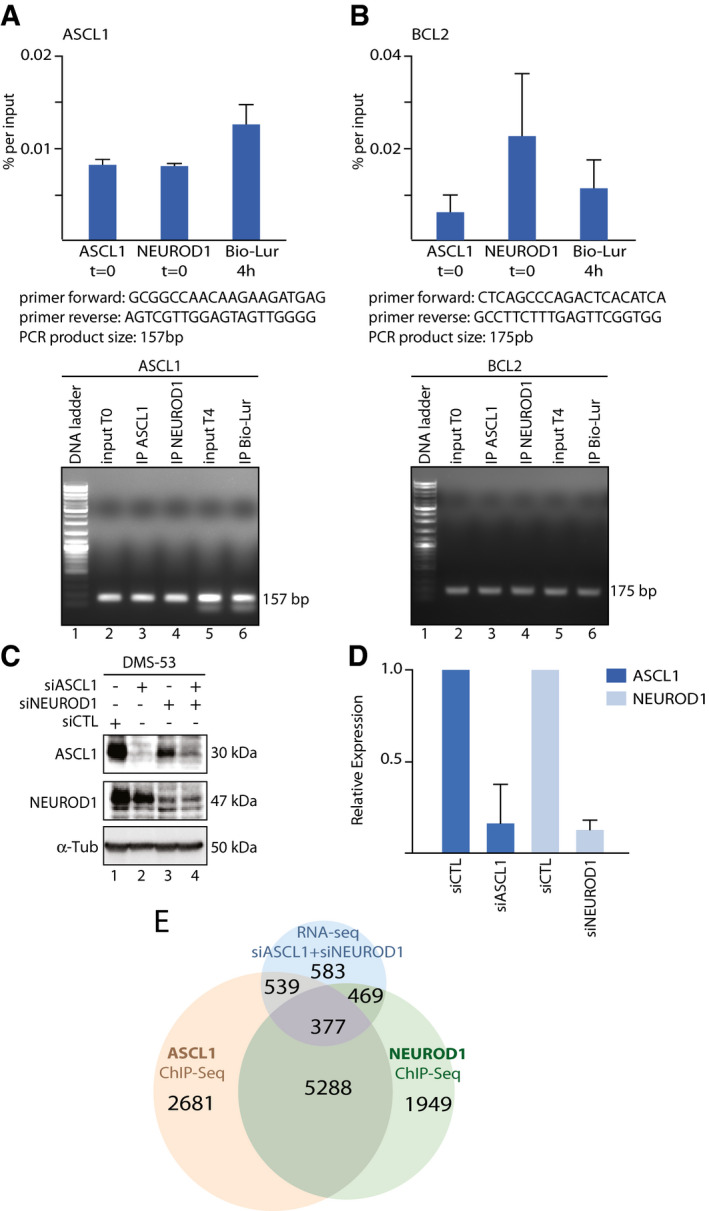
Identification of ASCL1‐ and NEUROD1‐dependent genes in ChIp‐seq/Bio‐Lur, Silencing of ASCL1/NEUROD1 and evaluation of ASCL1/NEUROD1‐dependent genes by RNA‐seq A, BChIP‐qPCR showing the enrichment of ASCL1, NEUROD1, and Lur (Bio‐Lur) at ASCL1 and BCL2 genes (upper panels) and the respective primer sequences, PCR product sizes, and specificity (lower panels showing Agarose signal at the respective amplicon sizes). Data are represented as mean ± SEM (*n* = 3 technical replicates).CWestern blot showing the silencing of both ASCL1 (lanes 2 and 4) and/or NEUROD1 (lanes 3 ad 4) in the DMS53 cell line as compared to nontargeting siRNA (siCTL, Lane 1). αtub is used as loading control.DHistogram of qPCR showing the silencing of ASCL1 (Dark Blue) and NEUROD1 (Light Blue) in the DMS53 cell line as compared to nontargeting siRNA (siCTL,). Data (represented as mean ± SEM, *n* = 3 technical replicates) are shown as relative mRNA expression normalized to nontargeting siRNA (siCTL).EVenn diagrams showing the overlap between genes downregulated in double silenced siASCL1 /siNEUROD1 DMS53 (blue circle) and ASCL1 and NEUROD1 ChIP‐seq (orange and green circles, respectively). Source data are available online for this figure.

After 4 h of 50 nM lurbinectedin treatment of DMS53 cells, ChIP‐seq analyses showed that most of the genes (7,963) targeted by the drug (Table 1) were recognized by either ASCL1 (7,230) or NEUROD1 (7,214). Of note, a similar number of genes were transcribed at *t* = 0 and *t* = 4 h, indicating a continuous active transcription under the control of ASCL1 (6,331, 87%) and NEUROD1 (4,741, 66%). In fact, between 75% and 85% of the genes were being probably actively transcribed as revealed by the presence of either RNAPII and H3K27Ac and ATAC signatures. More than 50% of them were already targeted by either ASCL1 (4,037, 56%) or NEUROD1 (4,786, 66%) underlining the specificity of the drug toward genes undergoing a transcriptional process. Among them, 2,177 (62%) of the ASCL1‐targeted and 1,873 (71%) of the NEUROD1‐targeted genes were found in an open chromatin environment (according to ATAC and H3K27Ac signatures), actively transcribed (as revealed by the presence of RNAPII) and bound by lurbinectedin (Table 1). We especially noticed that lurbinectedin targeted the promoter area of activated genes as exemplified for *ASCL1*, *BCL2*, *INSM1*, and *MYB* genes, all of them being targeted by ASCL1 and/or NEUROD1 activators, read by phosphorylated RNAPII and in open chromatin state according to the presence of H3K27Ac and ATAC signatures (Fig 3A–D; Appendix Fig S1A–D). Interestingly, Bio‐lur enrichment overlapped with CGG‐rich regions found in promoters. Moreover, it is worthwhile to notice that among the 2,194 downregulated genes that were targeted by lurbinectedin, 1,672 (76%) were bound by ASCL1 and NEUROD1 (Table 2), which underlined a certain degree of specificity of lurbinectedin toward these ASCL1‐ or NEUROD1‐“dependent” genes. RT‐PCR experiments then show that ChIP‐seq fractions indeed contain ASCL1‐ and/or NEUROD1‐targeted genes such as ASCL1 and NEUROD1 (*n* = 3 technical replicates; Figs EV3A and B). ChIP‐seq profiles summarized how the different components involved in the transcriptional process were positioned at the promoters from 3′ to 5′, RNAPII being at a certain distance from TSS as summarized Fig 3E. Notably, the bindings of RNAPII and lurbinectedin peaked at approximately 250 bp and 375 bp from the TSS, respectively (Figs 3F and G), suggesting that the drug might represent a roadblock for elongating RNAPII; the distance between lurbinectedin and RNAPII was < 0.1 kb in 30% of cases (Fig 3H).

**Table 2:** Lurbinectedin downregulated genes are categorized as a function of the target proteins.

Total targeted by Lur	2,194
Targeted by Lur and ASCL1/NEUROD1	1,672 (76%)
Targeted by Lur only (Housekeeping)	522 (9% HK)

The table shows the genes targeted by lurbinectedin and downregulated in DMS53 RNA‐seq and bound by ASCL1/NEUROD1. Housekeeping genes (HK) are shown in genes bound by lurbinectedin but not by ASCL1/NEUROD1.

Altogether, our data show that promoters with CGG‐rich regions located downstream to TSS of active ASCL1‐ and NEUROD1‐targeted genes are the preferential targets for lurbinectedin in SCLC cells.

### Lurbinectedin induces ubiquitin/proteasome degradation of elongating RNAPII

**Figure 4:**
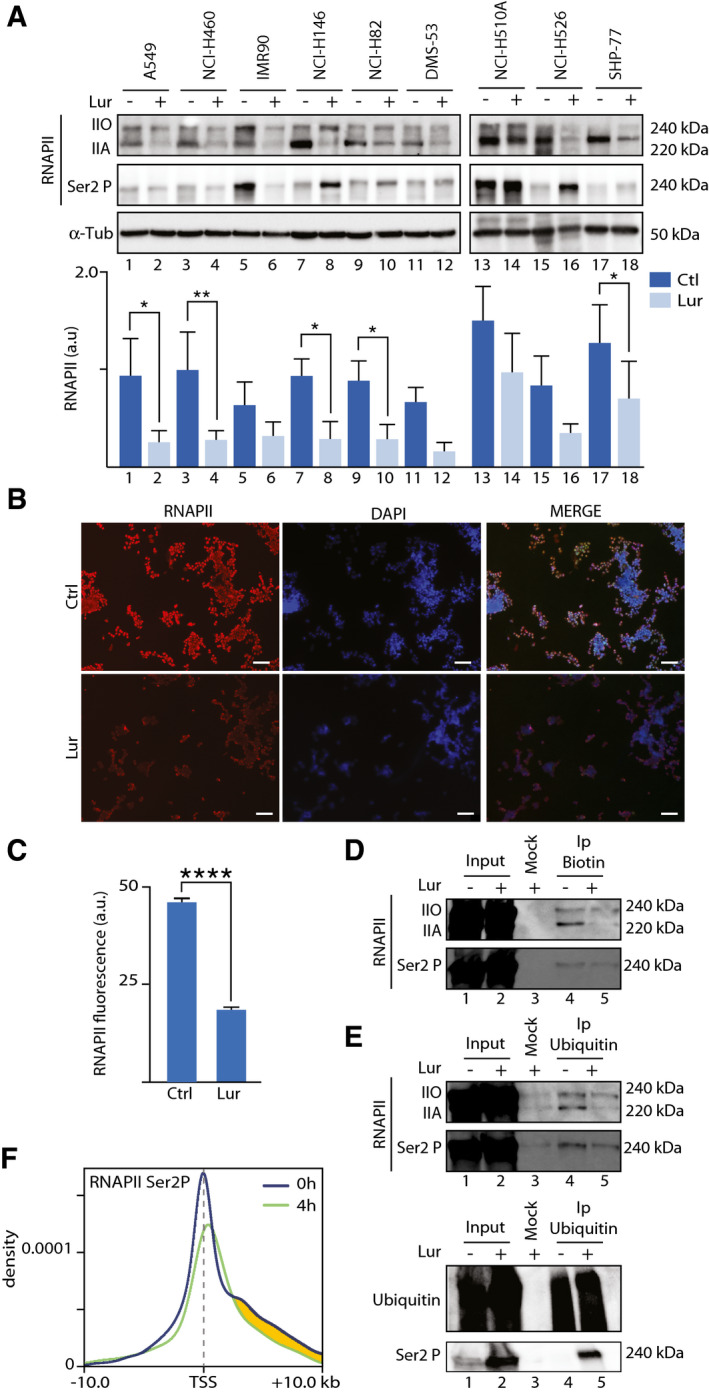
Lurbinectedin primes Ubiquitination and degradation of RNAPII AWestern blot showing RNAPII across the panel of A549 and NCI‐H460 NSCLC cells; IMR‐90 HFL cells; NCI‐H146, NCI‐H82, DMS‐53, NCI‐H510A, NCI‐H526, and SHP‐77 SCLC cells before (−) and after (+) treatment with lurbinectedin. α‐Tubulin (α‐Tub) is shown as loading control; lower panel, histogram showing the average of RNAPII before (dark blue) and after (light Blue) lurbinectedin treatment in arbitrary units (a.u.) normalized against α‐Tub. Data are presented as Mean ± SEM as determined by two‐way RM ANOVA and Šídák's multiple comparisons test (*n* = 3 biological replicas). ****P ≤ 0.0001.B, CImmunofluorescence of RNAPII (red) before (Ctrl) and after lurbinectedin treatment. DAPI (blue) was used to stain nuclei; scale bar is 65 μm. Data are presented as Mean ± SEM. ****P ≤ 0.0001, *P ≤ 0.00332, **P ≤ 0.0021, as determined by Student’s *t*‐test (*n* = 3 biological replicates).DAffinity purification of Bio‐lur fractions containing elongating RNAPII Ser2 (lane 4). Input (lanes 1–2) represents 10% of the starting material. Respective molecular weights are shown on the right (kDa).EUpper panel: GST‐pulldown of ubiquitinated proteins showing hypo‐ (IIA), hyper (II0)‐phosphorylated RNAPII (lanes 4–5 upper panel) and Ser2 Phosphorylated RNAPII (Ser2P) (lanes 4–5 lower panel) **a**fter lurbinectedin treatment. Empty beads (Mock) (lane 3) as a negative control; Lower Panel: GST‐pulldown of ubiquitinated proteins Ser2P) (lanes 4–5). Representative smear of successful ubiquitinated proteins pulldown (Lanes 4 and 5) as compared to input (lanes 1 and 2). Empty beads (Mock) (lane 3) as a negative control. Each Western blot is representative of three independent experiments.FDensity profile of annotated genes for RNAPII‐Ser2 P ChIP seq. Lines representing ChIP seq profile at 0 h (blue) and 4 h (green) after lurbinectedin treatment −10.0 and +10.0 kb to TSS coordinates (*P* = 0.005476). Decrease in density between 0 h and 4 h is highlighted in yellow. Source data are available online for this figure.

Western blot experiments revealed a sharp decrease of total RNAPII and more precisely hypo‐phosphorylated RNAPII (IIA), in all lurbinectedin‐treated SCLC as well as in the two NSCLC cells (Fig 4A and histogram). The phosphorylation of the carboxyl‐terminal domain of RNAPII largest subunit reflects different stages in the transcription process; RNAPII gets hyperphosphorylated (RNAPIIO) in the process of active transcription (Phatnani & Greenleaf, 2006). In parallel, immunofluorescence further showed a lower cellular abundance of RNAPII in DMS‐53 SCLC cells, 4 h after lurbinectedin treatment (Mean ± SEM = −27.60 ± 1.514, *t* = 18.23, *P* < 0.0001, *n* = 3 biological replicates; Fig 4B and C), which was accompanied by a decrease in ASCL1, NEUROD1, leading to a failure in the transcription process (Fig 2A and B, green curves).

To further investigate the fate of RNAPII, whole‐cell extracts from Bio‐lur‐treated DMS‐53 cells were subjected to affinity purification by streptavidin to isolate the biotin‐bound fraction. RNAPII phosphorylated at Ser2 (a hallmark of elongating RNAPII) was found among the precipitated proteins (*n* = 3 biological replicates; Fig 4D, lane 4). Moreover, further isolation of ubiquitinated proteins with Ubi‐GST beads (using a glutathione beads matrix), from the same DMS‐53 lurbinectedin‐treated extract, also pulled down phosphorylated Ser2‐RNAPII from the ubiquitin‐precipitated fractions (*n* = 3 biological replicates; Fig 4E, lane 5). Of note, the absence of RNAPIIA in the Ip‐Ubiquitin precipitated fraction indicates an RNAPII degradation process in progress.

In line with the above‐indicated observations, ChIP‐seq data showed the presence of Ser‐2‐phosphorylated RNAPII downstream of TSS in untreated DMS53 cells (*t* = 0), underlining an active ongoing elongation process (*P* = 0.005476; Fig 4F, blue curve). Importantly, after lurbinectedin treatment, we observed a decrease in the concentration of chromatin‐bound, Ser‐2‐phosphorylated RNAPII downstream of TSS (green curve, and yellow inlay).

The above data show that lurbinectedin treatment results in a rapid ubiquitin/proteasome‐dependent degradation of elongating RNAPII, the key molecule involved in RNA synthesis. Such degradation seems to be engaged when elongating RNAPII is arrested by lurbinectedin in SCLC cells (Fig 3E).

### Lurbinectedin downregulates the expression of ASCL1‐ and NEUROD1‐mediated genes

We next evaluated the gene expression changes induced by lurbinectedin treatment. RNA sequencing (RNA‐seq) of DMS‐53 cells (overexpressing both ASCL1 and NEUROD1) showed that 6,785 genes were downregulated 4 h after lurbinectedin treatment (Table 3), 2,194 (32%) of which being bound by the drug. Of note, 4,037 and 4,786 genes bound by either ASCL1 or NEUROD1, respectively, were targeted by lurbinectedin (Table 1).

**Table 3:** Evaluation of RNA synthesis before (*t* = 0) and after (*t* = 4 h) lurbinectedin treatment.

Cell lines	*t* = 0	*t* = 4	Cell lines	*t* = 0	*t* = 4	Downregulated genes		Overlapped genes between cell lines	Downregulated and targeted by Lur
DMS‐53 (A + N)	X		DMS‐53		X	A	6,784	A/B/C, 3,374	2,194 (32%)
NCI‐510A(A)	X		NCI H510(A) ‐		X	B	5,692	A/B, 4,400 (74%)	1,971 (33%)
NCI‐H82(N)	X		NCI‐H82(N)		X	C	5,247	A/C, 3,702 (71%)	1,789 (34%)
NCI‐H82(N)	X		NCl‐H510(A)	X		D	4,914		
DMS‐53(siA+siN)	X		DMS‐53 (A + N)	X		E	1,214		
siASCL1‐D	X		siASCL1‐D		X	G	6,794	A/G: 6,331	
siASCL1‐D	X		DMS‐53	X			1,698		
siASCL1‐D	X		NCI‐H82		X		4,959		
siNEUROD1‐D	X		siNEUROD1		X	H	6,755	A/H: 6,347	
siNEUROD1‐D	X		DMS‐53	X			465		
siNEUROD1‐D	X		DMS‐53		X		5,321		

Number of downregulated genes (column 7) for each cell line as well as for DMS‐53 silenced ones (columns 1 and 4) (columns 5–10). Letters are indicated for ease of overlap representations between conditions. Number of genes targeted by lurbinectedin and downregulated are shown and highlighted in percentages (Column 9). *n* = 3 biological replicas. Number of downregulated genes (column 7) for each cell line as well as for DMS‐53 silenced ones (columns 1 and 4) (columns 5–10). Letters are indicated for ease of overlap representations between conditions. Number of genes targeted by lurbinectedin and downregulated are shown and highlighted in percentages (Column 9). *n* = 3 biological replicas.

The gene expression pattern upon lurbinectedin treatment was also investigated in NCI‐H510A and NCI‐H82 SCLC cells, which overexpress ASCL1 and NEUROD1, respectively (Fig 1A). We observed that a significant number of genes were abrogated in these cells, 4 h after lurbinectedin treatment (Table 3). Indeed, 5,962 genes were downregulated in NCI‐H510A cells, (4,400 of which being common with DMS‐53 cells) and 33% (1971) of them being preferentially targeted by lurbinectedin. Similarly, 5,247 genes (3,702 being common with DMS‐53) were downregulated in NCI‐H82 cells, 34% (1789) of them being targeted by lurbinectedin.

We also investigated the regulatory function of either ASCL1 or NEUROD1 by transfecting DMS‐53 cells with small interfering RNA (siRNA) pool targeting either ASCL1 or NEUROD1. This resulted in siASCL1‐ and siNEUROD1‐DMS‐53 cells, as verified by western blot and RT‐PCR analyses (Fig EV3C and D). Unexpectedly, 7,913 and 7,844 genes were downregulated 4 h post lurbinectedin treatment in siASCL1‐ and siNEUROD1‐DMS‐53 cells, respectively (Table 3), suggesting that the silencing of either ASCL1 or NEUROD1 was not sufficient to fully circumvent a lurbinectedin effect. Interestingly, among the 1,698 genes downregulated upon silencing ASCL1, 930 genes were found downregulated by lurbinectedin in DMS53 cells; similarly, among the 464 genes downregulated upon silencing NEUROD1, 217 genes were also found downregulated by lurbinectedin in DMS53 cells (Appendix Fig S2). Moreover, most of the genes (80%) downregulated by lurbinectedin in siASCL1‐ and siNEUROD1‐DMS‐53 cells (6,331 and 6,347, respectively) were also found reduced in lurbinectedin‐treated DMS‐53 cells; only a small proportion of targeted genes was directly affected by the knock down of either ASCL1 or NEUROD1 (1,739 and 480 genes, respectively). This might result from the fact that the transcription factors ASCL1 and NEUROD1 both recognize E‐box response elements and that the invalidation of one of them might be compensated by the other one. Consequently, DMS‐53 cells were next simultaneously silenced for ASCL1 and NEUROD1 by siRNA transfection. Accordingly, we double silenced DMS53 cells and found that 1,214 genes were downregulated in our experimental conditions (Table 3). Fifty‐two percent (631) of them were being usually targeted either by ASCL1, NEUROD1, or both (Fig EV3E), knowing that lurbinectedin targeted 7,963 genes (Table 1).

Altogether our data demonstrated that lurbinectedin abrogated mainly ASCL1/NEUROD1‐targeted genes that were already involved in an active transcriptional process.

### Lurbinectedin blocks the growth of transcription‐addicted cells

**Figure 5:**
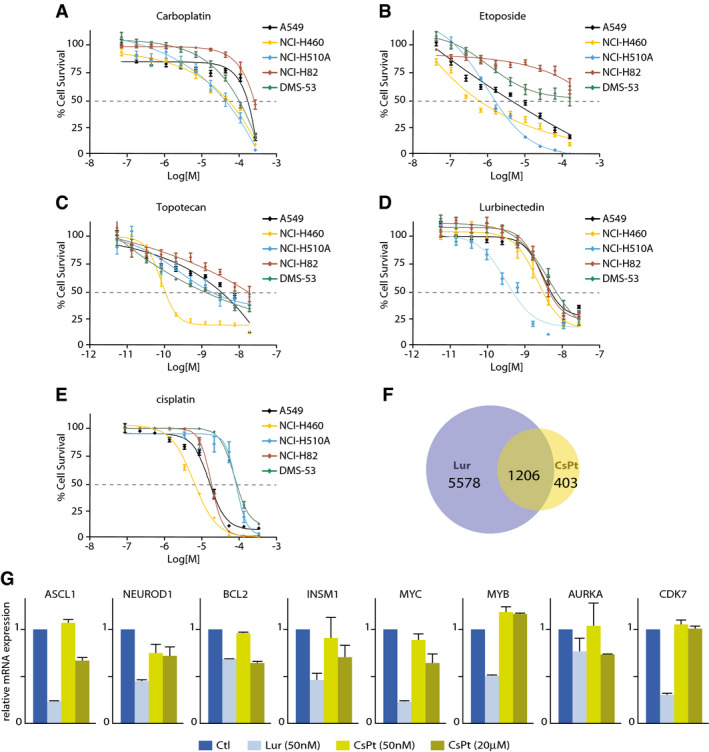
Lurbinectedin inhibits SCLC growth A–EDose–response of A549 (black), NCI‐H460 (yellow), NCI‐H510A (blue), NCI‐H82 (red), and DMS‐53 (green) cell lines after treatment with (A) Carboplatin, (B) Etoposide, (C) Topotecan (D) Lurbinectedin, and (E) Cisplatin. Data representing the half maximal inhibitory concentration (IC_50_) are expressed in molar concentrations. Cell survival is shown in percentage and 50% threshold is highlighted in dotted line. The data (presented as mean ± SEM) are the average of three independent experiments.FVenn diagrams showing the overlap between genes downregulated after 50 nM treatment with lurbinectedin (blue circle) and 20 µM treatment with cisplatin (yellow circle) in RNA‐seq.GRT‐PCR showing the downregulation of ASCL1, NEUROD1, BCL2, INSM1, MYC, MYB, AURKA, and CDK7 genes after 50 nM lurbinectedin treatment (light blue bars), 50 nM or 20 µM Cisplatin (CsPt, light yellow and dark yellow bars, respectively) as compared to untreated samples (Ctl, dark blue bars). Data are represented as Relative mRNA Expression normalized to Ctl. Data are presented as Mean ± SD (*n* = 2 biological replicates).

To further evaluate the efficiency of lurbinectedin, we next compared its activity with that of a panel of drugs currently used for SCLC therapy and known to disturb DNA processes, including carboplatin, cisplatin, etoposide, and topotecan. Carboplatin and cisplatin alkaloids preferentially bind adjacent guanine bases of the DNA all over the genome (Dasari & Yao, 2014). Etoposide prevents the re‐ligation function of Topoisomerase 2 (TOP2) by promoting the formation of a TOP2 cleavable complex (TOP2cc) and the subsequent formation of double‐strand DNA breaks (Burden *et al*, 1996). Topotecan intercalates DNA bases in the Topoisomerase‐I cleavage complex, preventing the re‐ligation of the nicked DNA strand (Li & Liu, 2001; Chhatriwala *et al*, 2006). The three SCLC cell lines DMS‐53, NCI‐H82, and NCI‐H510A as well as the NSCLC A549 and NCI‐H460 cells were treated in parallel with increasing amounts of lurbinectedin, carboplatin, cisplatin, etoposide, and topotecan, and their IC_50_ values were determined. The cell survival index was significantly higher compared to the one showed by lurbinectedin (Fig 5A–E and Table 4). Indeed, comparison of DMS‐53 sensitivity to carboplatin, cisplatin, etoposide, and topotecan revealed how lurbinectedin elicited the most potent effect with IC_50_ values in the nanomolar range (1–2 nM) whether other compounds acted at a micro‐ or millimolar scale (11.1 µM for etoposide, 87 mM for carboplatin, 75 mM for cisplatin, and 1.9 M for topotecan) (*n* = 3 biological replicates). All of the 23 different human SCLC cell lines tested responded to lurbinectedin in the low nanomolar scale, with ranges spanning from 0.12 nM to 6.84 nM, most of them showing a rapid impairment of cell growth (Table EV1), which paralleled RNAPII degradation (Fig 4A). Similarly, IC_50_ values of lurbinectedin‐treated ASCL1^high^ cell line NCI‐H510A (~1.1 nM) and NEUROD1^high^ cell line NCI‐H82 (~1.6 nM) were still within the nanomolar scale. Of note, the NSCLC cell lines, NCI‐H460 and A549, exhibited a higher sensitivity to lurbinectedin than to other drugs (Table 4), a point that should be further investigated. It, however, should be mentioned that ASCL1 was also found to be essential for the survival of a majority of both SCLC and NSCLC (Augustyn *et al*, 2014). Moreover, treatment of DMS53 cells by cisplatin under the same experimental conditions (50 nM drug concentration during 4 h) was not sufficient to significantly abrogate RNA synthesis in DMS53 cells (*n* = 3 biological replicates; Fig 5E). Increasing to 20 µM cisplatin concentration, we observed that only 1,609 genes were downregulated (Fig 5F). RT‐PCR show that cisplatin treatment hardly downregulated genes to the level of lurbinectedin as demonstrated for BCL2 and AURKA; the other genes were preferentially downregulated by 50 nM lurbinectedin (Fig 5G).

**Table 4:** IC_50_ values for each cell line treated with lurbinectedin, carboplatin, etoposide, and topotecan.

Cells	Lurbinectedin	Carboplatin	Etoposide	Topotecan
A549	1.51 × 10^−9^	1.29 × 10^−4^	6.76 × 10^−6^	3.26 × 10^−6^
NCI‐H460	1.06 × 10^−9^	3.70 × 10^−5^	1.16 × 10^−6^	8.95 × 10^−8^
NCI‐H69	5.03 × 10^−10^	2.18 × 10^−4^	3.50 × 10^−5^	1.41 × 10^−5^
NCI‐H146	3.49 × 10^−10^	1.28 × 10^−4^	1.28 × 10^−4^	3.13 × 10^−7^
NCI‐H510A	1.64 × 10^−10^	3.70 × 10^−5^	9.90 × 10^−7^	2.68 × 10^−7^
SHP‐77	2.46 × 10^−8^	2.76 × 10^−4^	2.18 × 10^−4^	3.95 × 10^−6^
NCI‐H82	1.32 × 10^−9^	2.49 × 10^−4^	3.12 × 10^−3^	1.90 × 10^−5^
DMS‐53	2.12 × 10^−9^	8.73 × 10^−5^	> 1.00 × 10^−4^	1.97 × 10^−6^
NCI‐H526	1.26 × 10^−10^	4.13 × 10^−5^	9.29 × 10^−7^	2.32 × 10^−8^

The above data underlined the high sensitivity of lurbinectedin to downregulate a large number of genes (being likely under a transcriptional process) in DMS53 cells.

### Lurbinectedin downregulates key genes involved in tumorigenesis

**Figure EV4:**
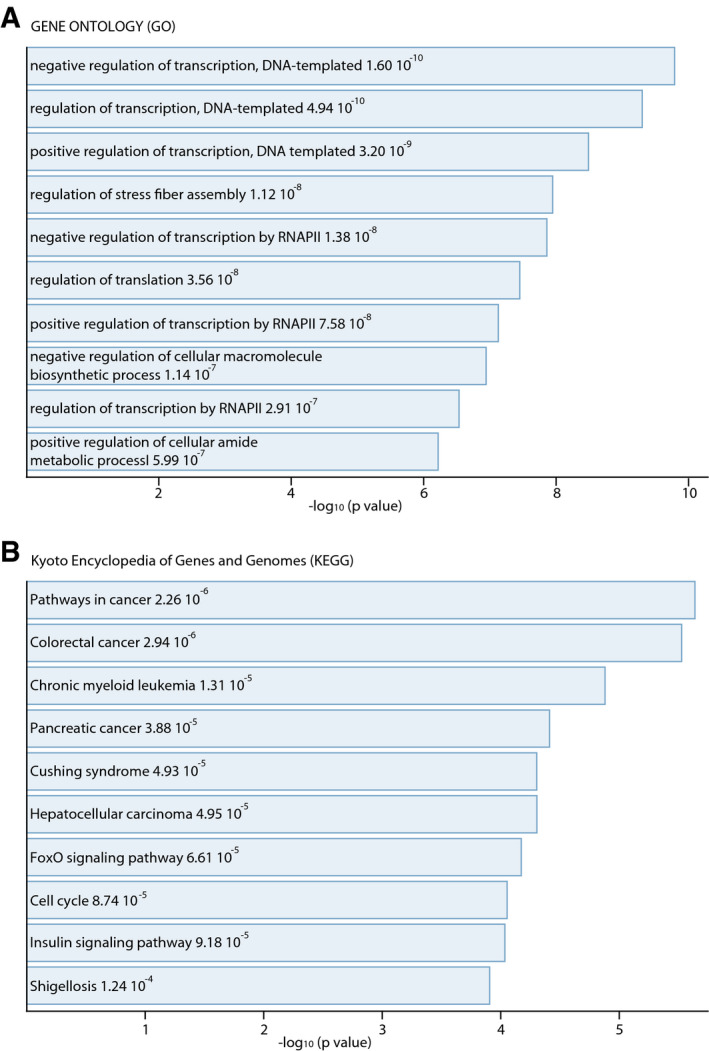
Gene ontology pathway of ASCL1‐ and NEUROD1‐dependent genes A, BGene Ontology (A) and pathway analysis (B) of the intersection between Fig 1 B and C representing the most enriched and statistically significant terms according to GO Biological Processes and KEGG pathways. *P*‐values are shown as −log_10_.

**Figure 6:**
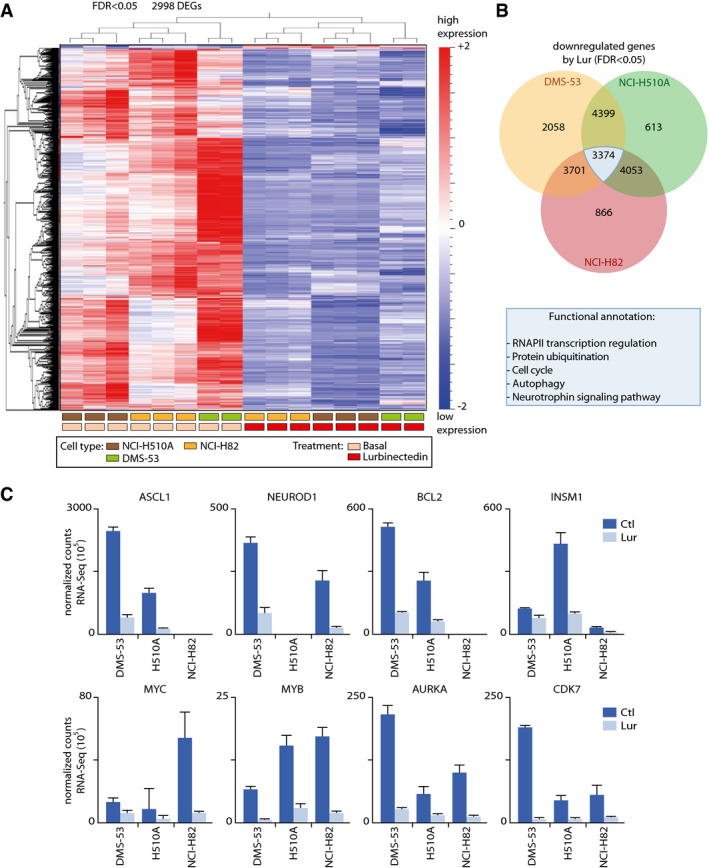
Downregulation of genes involved in SCLC tumorigenesis by lurbinectedin Heatmap of the transcriptomic dysregulation caused after treatment with lurbinectedin in five different cell lines of SCLC ‐DMS‐53 (green rectangle), NCI‐H510A (Brown rectangle), and NCI‐H82 (Yellow Rectangle) with a False Discovery Rate < 0.05 (2,998 common differentially expressed genes, DEGs) shows that the vast majority of genes are downregulated (*n* = 3 biological replicates). Color legends correspond to the three cell lines and ctl (basal ‐ Pink rectangles), as well as samples treated with lurbinectedin (Red rectangles).Venn Diagrams comparing DEGs with FDR < 0.05 in three different cell lines after treatment with lurbinectedin shows overlapping among the three DEG analyses. 3,374 genes are commonly downregulated in the three cell lines, independently of their ASCL1 or NEUROD1 expression levels. Overrepresentation analysis of the common genes downregulated in the three cell lines after lurbinectedin treatment (blue table).Lurbinectedin downregulates genes that have pivotal functions in the pathogenesis of SCLC as well as in the tumorigenic properties of the cells (ASCL1, NEUROD1, BCL2, INSM1, MYC, MYB, AURKA, and CDK7). Data are expressed as normalized counts before (dark blue) and after (light blue) lurbinectedin treatment. Data are presented as Mean ± SD (*n* = 3 biological replicates).

We next sought to understand whether among the whole set of genes affected by lurbinectedin there were important genes involved in SCLC tumorigenesis. We hence overlapped our Chem‐seq dataset with the data from whole RNA‐seq of DMS‐53 cells after lurbinectedin treatment. Gene Ontology analysis of the molecular functions related to the gene set downregulated by lurbinectedin in DLS53 cells showed the involvement in transcription which is in line with the high specificity of lurbinectedin toward CpG‐rich motifs located within gene promoter area (Fig EV4A). By analyzing the KEGG pathways, we observed that genes downregulated by lurbinectedin are genes mostly involved in carcinogenesis (Fig EV4B). Gene expression analysis of the RNA‐seq data across the three SCLC cell lines (DMS‐53, NCI‐H82 not expressing ASCL1; and NCI‐H510A ‐not expressing NEUROD1) treated with lurbinectedin versus basal condition, made by a multi‐group comparison revealed a great level of transcriptional dysregulation, showing 2,998 genes whose expression was altered with a FDR < 0.05. As observed in a heat map with z‐score normalization across the sample, more than 95% of those dysregulated genes are downregulated, demonstrating the significant level of transcription inhibition by lurbinectedin in SCLC cells (Fig 6A). It should be pointed out that RNA‐seq data show that these three cell lines shared in untreated conditions, 10,110 transcribed genes while expressing up to 13,000 genes each of them (Appendix Fig S3A). Moreover, (Appendix Fig S3B and C) of the 5,357 transcribed genes bound by ASCL1 in DMS53, 4,370 were commonly found to be transcribed in NCI‐H82 (NEUROD1 only) cell line, whereas 4,938 were found to be commonly transcribed in NCI‐H510A (ASCL1 only) cell line (Fig 1B). Of the 4,551 transcribed genes bound by NEUROD1 in DMS53, 3,649 were commonly found to be transcribed in NCI‐H82 (NEUROD1 only) cell line, whereas 4,163 were found to be commonly transcribed in NCI‐H510A (ASCL1 only) cell line (Fig 1C). If we assess the effect of lurbinectedin in each SCLC cell line, before and after treatment with the drug, by Differential Gene Expression Analysis (DESQ2, FDR ≤ 0.05), a higher number of dysregulated genes (each comparison is more homogeneous within the same cell type) would be obtained. Focusing on the transcriptional downregulation produced by lurbinectedin on each of the three original cell lines with different endogenous levels of ASCL1 and NEUROD1 (DMS‐53, NCI‐H82 and NCI‐H510A), we observed that there are 3,374 genes that coincide among the three different DESQ2 comparisons (Fig 6B; Appendix Fig S4). Additionally, pathway analysis further revealed that those genes were essential for the biology of the SCLC, showing affectation toward RNAPII transcription regulation, ubiquitin‐mediated pathway, cell cycle and autophagy.

**Figure EV5:**
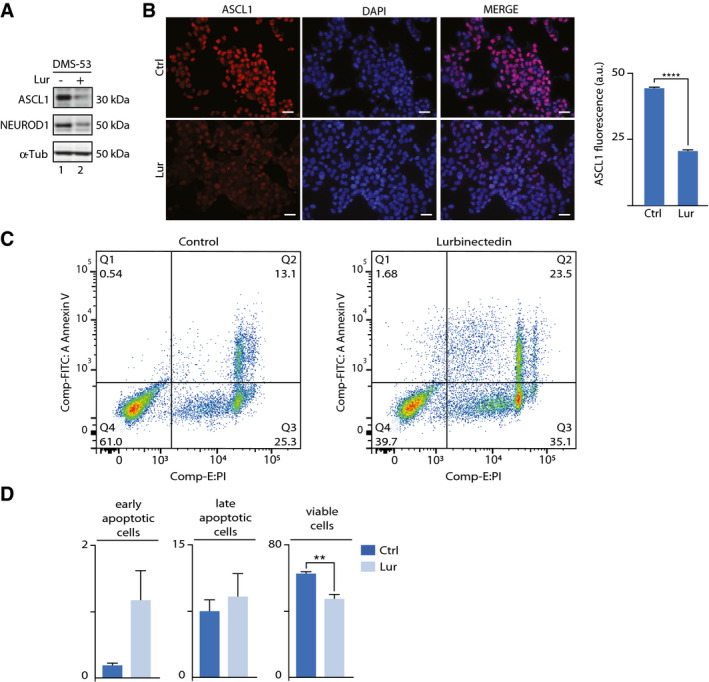
ASCL1 and NEUROD1 expression in DMS‐53 cell line before and after treatment with lurbinectedin Western blot showing ASCL1 and NEUROD1 expression in DMS‐53 cell line before (−) and after (+) lurbinectedin treatment. α‐Tub is shown as loading control. Each blot is representative of at least three different experiments.Immunofluorescence showing the decrease in ASCL1 (red) after lurbinectedin treatment. DAPI (blue) was used to stain nuclei; scale bar is 30 μm. (right panel) Histogram showing the average fluorescence signal of ASCL1 before and after lurbinectedin treatment in arbitrary unit (a.u.). Data are presented as Mean ± SEM. ****P ≤ 0.0001 as determined by Student’s *t*‐test (*n* = 3). Each assay is representative of at least three different experiments.Lurbinectedin triggers apoptosis in DMS‐53 cells. Annexin V‐FITC/PI analysis of apoptosis in DMS‐53 cancer cells before (left panel) and after (right Panel) 50 nM lurbinectedin treatment. Cells were incubated with lurbinectedin for 24 h. The upper left panel (Q1) displays early apoptotic cells, whereas the lower right panel (Q3) represents late apoptotic cells. Lower left panel (Q4) represents viable cells.Histogram showing the percentage of early apoptotic, late apoptotic, and viable cells from Control‐ (Ctrl, dark blue) and lurbinectedin (Lur, light blue)‐treated groups. Data are presented as Mean ± SEM. **P ≤ 0.0021 as determined by Student’s *t*‐test (*n* = 3 technical replicates). Source data are available online for this figure.

From all the genes significantly downregulated by lurbinectedin, we detected several crucial genes which involvement is pivotal in the pathophysiology of the disease. First, both *ASCL1* and *NEUROD1* genes were themselves downregulated by lurbinectedin after only 4‐h treatment of the three SCLC cell lines so far tested (Fig 6C), resulting in a downregulation of all their responsive genes. Indeed western blot analysis as well as immunofluorescence assays showed the decrease in ASCL1 and NEUROD1 protein level in DMS‐53 cells (Mean ± SEM = 22.40 ± 0.4140, *t* = 54.11, *P* < 0.0001; Fig EV5A and B). For example, the downregulation of the pro‐survival gene BCL2 that regulates cell death, pointed a role for lurbinectedin in triggering apoptosis of SCLC cells (Li *et al*, 2021). Insulinoma‐associated 1 (*INSM1*), a highly expressed gene in several SCLC tumors (Lan *et al*, 1994), which is used as a cytoplasmic marker for neuroendocrine differentiation of tumor cells (and indirectly in SCLC tumorigenesis), was also downregulated in the three cell lines. Strikingly, the *MYC* gene, a major oncogene playing a role in SCLC fate determination (Ireland *et al*, 2020), was listed among the most significantly downregulated genes. We found that lurbinectedin elicited a profound effect in other members of the *MYC* family such as *MYB*, which was transcriptionally arrested. *AURKA*, an important factor in tumorigenesis and a potential target for SCLC therapy since its reduced expression inhibited cell proliferation (Lu *et al*, 2014), was significantly downregulated in all the cell lines so far tested in our experimental conditions. *CDK7* a central component of the protein‐coding gene transcription abrogation will undoubtedly disturb cellular life. Indeed, abrogating its kinase activity as part of the general transcription factor TFIIH toward the transactivation of nuclear receptors as well as the phosphorylation of RNAPII, two events that condition optimal RNA synthesis (Compe *et al*, 2019).

To verify whether lurbinectedin might trigger apoptosis in SCLC as suggested by the abrogation of the BCL2 apoptosis regulator, we analyzed the apoptotic rate of DMS‐53 cells 24 h after lurbinectedin treatment. Staining and FACS analysis of DMS‐53 with Annexin V and Propidium Iodide showed that lurbinectedin induced a decrease in the viability of cells (39.7%) compared to the nontreated ones (61.0%) (Mean ± SEM = −14.90 ± 2.389, *t* = 6.237, *P* = 0.0034) (*n* = 3 biological replicates), as shown by Annexin V‐, PI‐ group (Q4). (Fig EV5C and D). In DMS‐53 cells, lurbinectedin at least doubled the population of cells in different stages of programmed cell death as shown in the gates for both early (0.54% vs 1.68%, quadrant Q1) and late (25.3%vs 35.1% quadrant Q3) apoptosis.

In summary, our results show that lurbinectedin efficiently takes advantage of the tumorigenic properties of SCLC cells by hindering the aberrantly high rate of transcriptional activity mediated by both ASCL1 and NEUROD1 and downregulated target genes commonly overexpressed in SCLC, finally impairing oncogenic programs and forcing SCLC cell apoptosis.

## Discussion

Among the standard care treatments for lung cancer in general, and SCLC in particular, platinum derivatives that covalently bind DNA have been widely accepted and used in the clinic. Due to their own structure, these drugs bind guanine bases all over the genome generating G(X)G intra‐ or inter‐strand cross‐linking, preventing the proper functioning of DNA processes such as replication or transcription. However, the broad spectrum of these agents and the requirement of high doses results in great levels of toxicity, rather intolerable for patients to receive. In comparison with the wide spectrum of DNA‐binding drugs, other chemical compounds, in addition to binding DNA, are able to specifically impair key regulatory domains involved in the modulation of gene expression, representing a novel concept in the treatment of transcription addiction‐driven cancers such as SCLC. The better understanding of the tumor growth driven by the overexpression of transcription activators, and consequently by transcriptional hyperactivity, has offered new perspectives in designing the targets to abrogate cancer growth. Exploiting the transcriptional dependency of cancer cells (here mainly directed by two well‐known DNA‐binding proteins: ASCL1 and NEUROD1) as an Achilles’s heel to thwart their own tumorigenic potential, represents a key actionable mechanism against SCLC. The present study takes advantage of the identification of the SCLC‐A and SCLC‐N subgroups of SCLC cells which overexpress ASCL1 and NEUROD1 transcriptional activators, respectively, in order to first study their transcriptional programs and second to molecularly analyze the effect of the marine‐derived alkaloid lurbinectedin, that has proven clinically meaningful effects in metastatic SCLC (Singh *et al*, 2021).

We have observed that the highly overexpressed ASCL1 and NEUROD1 activators target more than one third of the total number of genes expressed in SCLC (8,131 and 7,329, respectively; Table 1, Fig 1B and C). ASCL1‐ and NEUROD1‐targeted genes are very similar (likely due to some similarities between their cognate E‐boxes; Fig 1D), explaining at least partially common clinical outcomes between SCLC‐A and SCLC‐N groups of patients (Fig 1B and C, lower panel). Overlap between ASCL1 and NEUROD1 ChIP‐seq dataset further confirmed an enrichment of transcription‐related processes as the most significant scored terms (Fig 1B and C, and Table 1), thus strengthening the importance of these two transcription factors in transcription addiction for SCLC. In line, Gene Ontology and KEGG pathway analysis of ASCL1‐target genes and NEUROD1‐target genes in our RNA‐seq dataset showed that ASCL1 and NEUROD1 are involved in DNA metabolic process mainly devoted to RNAPII transcription regulation and cancer‐related pathways (Fig EV4A and B).

Up to 60% (56 and 66%, respectively) of the RNA transcripts were synthesized from ASCL1‐ and NEUROD1‐targeted genes (Tables 1 and 3), clearly defining them as main players in the transcriptional addiction of SCLC. Indeed, the promoters of a large number of genes are either in an open chromatin state (as judged by the H3K27Ac mark and ATAC signature) and/or involved in an active transcriptional process (according to the presence of elongating RNAPII; Fig 2C–E, Table 1). This transcriptional status was exploited by lurbinectedin that specifically targets the CGG motifs mainly found in CpG islands located downstream of gene promoters. As a consequence, lurbinectedin treatment imposes a profound transcriptional dysregulation of the SCLC cells. Around 18% of the drug binds in the surroundings of gene promoters (Figs 2G and EV2A). Up to 7,963 genes (25% of the genome) are targeted by lurbinectedin (Table 1).

It was interesting to notice that a large number of ASCL1‐ and NEUROD1‐targeted genes were in an open chromatin conformation and targeted by RNAPII (4,089 and 3,215 respectively). Genome browser visualization showed that in untreated conditions, ASCL1 or NEUROD1 bound to a number of genes as exemplified for *ASCL1*, *BCL2*, *INSM1*, and *MYB* being read by elongating RNAPII and in open chromatin state according to the presence of H3K27Ac and ATAC mark (Fig 3A–D, Appendix Fig S1). These genes were found to be targeted by lurbinectedin at their CGG‐rich regions located downstream from their TSS as visualized at the bottom of each panel. Moreover, among the 2,194 downregulated genes that were targeted by lurbinectedin, 1,672 (76%) were bound by ASCL1 and NEUROD1 (Table 2), which underlined a certain degree of specificity of lurbinectedin toward activated genes that are mainly under the control of either ASCL1 or NEUROD1 transcription factors. We speculate that the remaining 24% are either genes that might be regulated by the ASCL1‐ and/or NEUROD1‐targeted gene products and/or are simply housekeeping genes.

The binding of lurbinectedin or structural analogs leads to a stall of elongating RNAPII (Santamaria Nunez *et al*, 2016). We demonstrated that RNAPII was stalled upstream of lurbinectedin adduct (Fig 3E–H) and that it underwent further ubiquitination rapidly followed by degradation after lurbinectedin treatment (Fig 4A–C). Such an ubiquitin/proteasome degradation process, already observed upon cisplatin or UV treatment, could be initiated by CSA and CSB proteins, part of a ubiquitin ligase complex (Groisman *et al*, 2006; Anindya *et al*, 2010; Ho *et al*, 2021). These proteins (Epanchintsev *et al*, 2017) as well as others, such as SUG1, a proteasome subunit (Fraser *et al*, 1997; Weeda *et al*, 1997), were found associated with RNAPII machinery upon genotoxic stress. In addition to its direct effects by targeting active promoters and blocking transcription as previously observed (Santamaria Nunez *et al*, 2016), lurbinectedin binding promotes persistent DNA breaks in its vicinity, which might be considered as a third line of action for this drug. These DNA breaks likely result from failures in DNA repair pathways such as Nucleotide Excision Repair (NER) and Inter‐cross‐linking (ICL) DNA repair, which are solicited to eliminate lurbinectedin bound to DNA (Santamaria Nunez *et al*, 2016).

Compared to other anti‐tumoral drugs, such as carboplatin and cisplatin, that bind all over the genome, as well as topotecan and etoposide that target topoisomerases and are present in nondefined DNA sequences, lurbinectedin exhibits a clear specificity by preferentially targeting the CpG motifs found in 70% of gene promoters. Such DNA sequence specificity might explain, in part, the nano‐molar doses required for lurbinectedin anti‐tumoral treatment, while micromolar or even millimolar (carboplatin) concentrations are required for other therapeutic drugs (Fig 5 and Table 4). In addition, cisplatin downregulated a much lower number of genes, half of them, however, being targeted by ASCL1 and/or NEUROD1. Moreover, it must be pointed out that, with the exception of BCL2 and AURKA genes, cisplatin treatment hardly downregulated genes in SCLC to the same extent as lurbinectedin. Rather, in comparison to cisplatin, the remaining majority of the genes were more profoundly affected by 50 nM lurbinectedin (Fig 5G). In addition, of the 1,609 downregulated genes after high‐dose cisplatin treatment, only 690 were targeted by ASCL1, 634 by NEUROD1 and 462 by both ASCL1 and NEUROD1.

It was also interesting to note that a large amount (5,143) of downregulated genes were similarly targeted (and certainly regulated) by either ASCL1 or NEUROD1 which could partially explain some identical clinical outcomes between these two subtypes of SCLC (Fig 1B and C, lower panel). ASCL1 and NEUROD1 also exert specific bindings, as observed by the significant number of genes that are regulated by either one or the other of these transcription factors. Both ASCL1 and NEUROD1 genes were themselves downregulated by lurbinectedin (Fig 6C). ASCL1 is a transcription factor required for the proper development of pulmonary neuroendocrine cells (Augustyn *et al*, 2014) while NEUROD1 is a neuronal/neuroendocrine protein that helps migration and survival of neuroendocrine carcinomas (Osborne *et al*, 2013). Both have been described as master regulators in the transcriptional addiction of SCLC and define the two major molecular genotypes of the disease, playing crucial roles in promoting malignant behavior and survival (Rudin *et al*, 2019; Gay *et al*, 2021).

Our work demonstrates the crucial role of ASCL1 and NEUROD1 activators in regulating the expression of genes involved in tumorigenesis (some of which have even been previously identified as therapeutic targets) and how lurbinectedin might abrogate their function. As first example, *BCL2*, that regulates cell death by either inhibiting or inducing apoptosis, is also dramatically downregulated by lurbinectedin. *BCL2* has already been proposed as a therapeutic target as its pharmacological inhibition stops ASCL1‐dependent tumor growth (Shoemaker *et al*, 2008; Augustyn *et al*, 2014; Gay *et al*, 2021). Similarly, transcription termination factor 1 (*TTF1* also named *NKX2‐1*), which is particularly highly expressed in SCLC‐A subtype, is also clearly downregulated after lurbinectedin treatment. *TTF1* has been shown to promote SCLC cell growth and to contribute to neuroendocrine and antiapoptotic gene expression programs (Hokari *et al*, 2020). Strikingly, the *MYC* gene, a major oncogene playing a role in SCLC fate determination (Ireland *et al*, 2020), was also listed among the most significantly downregulated genes. We found that lurbinectedin elicited a profound effect in other members of the *MYC* family such as *MYB* that was also transcriptionally arrested. Although the overexpression of different genes belonging to the *MYC* family has been considered as mutually exclusive in different SCLC subtypes (Bragelmann *et al*, 2017), the high specificity of lurbinectedin enabled a prompt downregulation on the overall *MYC* family of oncogenes in different SCLC cellular backgrounds. Abrogation of INSM1 (proposed as target gene for SCLC cancer therapy; Pedersen *et al*, 2006), that exerts a crosstalk with the sonic hedgehog transcription pathway, and also critical for NE differentiation, by lurbinectedin might also affect the NE lung cancer development. The reduced expression of *AURKA*, an important factor in tumorigenesis, inhibited cell proliferation and was also proposed as a potential target for SCLC therapy (Lu *et al*, 2014). Additionally, by targeting CDK7, it is clear that lurbinectedin affects a key component at the crossroad of transcription, DNA repair, and cell cycle (Compe & Egly, 2016). CDK7 inhibition has been demonstrated to disrupt cell cycle progression and to induce DNA replication stress and genome instability in SCLC, being another candidate for targeted therapy in SCLC (Zhang *et al*, 2017). In summary, many pivotal genes involved in SCLC neuroendocrine features and tumorigenic properties (inhibition of apoptosis, cell survival, etc.) are critically downregulated by lurbinectedin, acting as a specific therapy to the most crucial molecules causing pathogenicity in SCLC.

Besides the downregulation of *ASCL1* itself, that was shown to be essential for the survival of a majority of lung cancers, we discovered that in our experimental conditions, lurbinectedin targets and downregulates 18 genes among the 72 ASCL1‐dependent gene expression signatures previously identified (such as the antiapoptotic regulator *BCL2*) as neuroendocrine differentiation markers in SCLC (Table EV2) that constitute potential novel druggable targets (Augustyn *et al*, 2014).

This work sheds new light on the mechanism of action of ASCL1 and NEUROD1 in SCLC and demonstrates how specifically targeting certain areas of the genome results in a more effective chemotherapy, thus providing a step forward for precision medicine. By recognizing CpG motifs located at promoters of activated genes, lurbinectedin gains in specificity when compared to all the other DNA binders used in the clinic up to date. This could limit some of the secondary effects observed with other therapeutic approaches. Moreover, such specificity of action of lurbinectedin allows the identification of active genes involved in tumorigenesis and in neuroendocrine reprogramming of the cell. This new drug has completely changed the therapeutic landscape of SCLC since its recent accelerated approval as a monotherapy in metastatic disease by the FDA (Kepp *et al*, 2020; Shinn *et al*, 2020; Trigo *et al*, 2020; Baena *et al*, 2021; Cortinovis *et al*, 2021; Singh *et al*, 2021). This work provides important molecular information underlying its efficacy.

## Materials and Methods

### Cell culture and treatments

The following cell lines were obtained from the ATCC: A549 (lung adenocarcinoma; CCL‐185), DMS‐53 (small‐cell lung carcinoma; CRL‐2062), IMR‐90 (normal lung; CCL‐186), NCI‐H69 (small‐cell lung carcinoma; HTB‐119), NCI‐H82 (small‐cell lung carcinoma; HTB‐175), NCI‐H146 (small‐cell lung carcinoma; HTB‐173), NCI‐H460 (large cell lung carcinoma; HTB‐177), NCI‐H510A (small‐cell lung carcinoma; HTB‐184), NCI‐H526 (small‐cell lung carcinoma; CRL‐5811), and SHP‐77 (small‐cell lung carcinoma; CRL‐2195). The cells were authenticated and tested for mycoplasma contamination. All cell lines were cultured in the medium and conditions recommended by the supplier and supplemented with 10%FBS, 2 nmol/L l‐glutamine, and penicillin–streptomycin mix (Sigma). For lurbinectedin treatment, cells were seeded and grown to subconfluency before the addition of the drug to the culture medium after having optimized drug concentration (50 nM) and time of lurbinectedin treatment (4 h).

### Cell proliferation

Cell proliferation was studied from conversion of [3‐(4,5‐dimethythiazol‐2‐yl)‐2,5‐diphenyl] tetrazolium bromide (MTT) (Sigma) to its colored reaction product, MTT formazan, which was dissolved in DMSO so as to measure absorbance at 450 nm with POLARStar Omega Reader (BMG Labtech). Cells were seeded in 96‐well plates. Serial dilutions of lurbinectedin, carboplatin, cisplatin, or etoposide were added to the medium. Exposure to the drugs was maintained over 72 h. Determination of IC_50_ values was performed by iterative nonlinear curve fitting using the Prism 5.0 statistical software (GraphPad). The data presented are the average of three independent experiments.

### siRNA transient transfection

For siRNA transfection, pools of oligonucleotides targeting either ASCL1 or NEUROD1 mRNA (siRNA) (SMARTpool – Horizon Discovery) were transfected in DMS‐53 cells at a concentration of 100 nM. SMARTpool Ctrl RNA oligonucleotides without any target mRNA (siCTL) were used as control. siRNA transfection was performed using Lipofectamine 2000 Transfection Reagent (Thermo Fisher Scientific), with antibiotic‐free culture medium according to the manufacturer’s instructions. Cells were harvested 48 h after transfection.

### Western blotting analysis

For immunoblotting, cell protein extracts were prepared following standard procedures in RIPA buffer in the presence of protease inhibitors (Complete, Roche Diagnostics) and phosphatase inhibitors (PhosStop, Roche Diagnostics). After quantitation with the Micro‐BCA Protein Assay Kit (Thermo Fisher Scientific), 25 µg of protein was separated by SDS–PAGE and transferred to PVDF membranes (Immobilon‐P, Millipore). After using appropriated primary and secondary antibodies, blots were developed by a peroxidase reaction using ECL detection system (Amersham‐G.E. Healthcare). Antibodies used were Recombinant Anti‐NeuroD1 antibody [EPR4008] (ab109224; 1:1,000 dilution), Anti‐MASH1/Achaete‐scute homolog 1 (Abcam ab211327; 1:1,000 dilution), Mouse Anti‐RNAPII 7c2 (IGBMC Antibody facility; 1:1,000 dilution), and Anti‐Phospho‐Serine 2 RNAPII (Abcam ab5095; 1:1,000 dilution). Anti α‐Tubulin (SIGMA T5168) (Novus Biologicals hVIN‐1 NB600‐1293; 1:1,000 dilution) was used as loading control. All the blots are representative of at least three independent experiments.

### Immunostaining

DMS‐53 cells were treated with 50 nM of lurbinectedin for 4 h, washed, fixed (4% paraformaldehyde, PFA), permeabilized (0.1% Triton X‐100), and blocked (5% bovine serum albumin). Cells were incubated with the respective primary antibody for 1 h at 37°C. Antibodies used were the following: rabbit Recombinant Anti‐MASH1/Achaete‐scute homolog 1 (Abcam ab211327; 1:100 dilution) and Mouse Anti‐RNAPII 7c2 (IGBMC Antibody facility; 1:100 dilution; Santamaria Nunez *et al*, 2016). Thereafter, the cells were washed and incubated with the AlexaFluor 594 secondary goat anti‐rabbit IgG (Invitrogen; 1:1,000 dilution) and Hoechst 33342 (Sigma) for 1 h at room temperature and mounted with Mowiol mounting medium. Pictures were taken with Leica DM IRM fluorescence microscope equipped with a DFC 340 FX digital camera (Leica). Quantitation of the fluorescence signals was performed with Fiji software (Schindelin *et al*, 2012).

### Real‐Time RT‐PCR

DMS‐53 cells were treated with 50 nM of lurbinectedin for 4 h, and total RNA was extracted. For all RNA‐related experiments, we used QIAquick RNA purification kit (Qiagen), SuperScript II RT reverse transcription kit (Thermo‐scientific), and Quanti‐Tect SYBR Green PCR MasterMix (Qiagen) according to the manufacturer’s recommendations.

### *In vitro* detection of ubiquitinated proteins

For *in vitro* pulldown of ubiquitinated proteins, Ubiquitinated Protein enrichment kit (Merck 662200) was used. Briefly, pellets from DMS‐53 treated or not with lurbinectedin, were lysed by using Lysis Buffer (50 mM HEPES (pH 7.5), 5 mM EDTA, 150 mM NaCl, and 1% Triton^®^ X‐100 detergent). 500 µg of protein extracts was incubated with 40 µl of GST‐bound ubiquitin beads slurry for 4 h at 4°C and then washed in lysis buffer. Ubiquitinated proteins were eluted by boiling the beads in Laemli 2X at 95C for 5 min and then analyzed by SDS–PAGE. Blots were incubated with antibody against Ubiquitin (Santa Cruz), Anti‐Phospho‐Serine 2 RNAPII (Abcam ab5095), and Mouse Anti‐RNAPII 7c2 (IGBMC Antibody facility; Santamaria Nunez *et al*, 2016).

### Target enrichment analysis

For target enrichment analysis, protein extracts from DMS‐53 cells treated or not with lurbinectedin, were incubated overnight with a molar excess of Bio‐lur (1 µM). The resulting complexes were then immunoprecipitated by using Streptavidin Magnetic Beads (M280–Thermo Scientific) for 2 h at 4°C, washed in lysis buffer (50 mM HEPES (pH 7.5), 5 mM EDTA, 150 mM NaCl, and 1% Triton^®^ X‐100 detergent), and eluted by boiling the beads in Laemli 2X at 95°C for 5 min and then analyzed by SDS–PAGE. Blots were incubated with antibody against Ubiquitin (Santa Cruz sc‐8017), Anti‐Phospho Serine 2 RNAPII (Abcam ab5095), and Mouse Anti‐RNAPII 7c2 (IGBMC Antibody facility).

### FACS, Annexin V/PI apoptosis detection

DMS‐53 cells were seeded on to 6‐well plates, treated with 50 nM lurbinectedin and finally harvested via trypsinization 24 h later. Cells were further stained using annexin V‐FITC apoptosis detection kit (Abcam ab14085). Cells were immediately analyzed on FACS analyzer. Analysis was performed using FlowJo™ v10.8.1 Software (BD Life Sciences).

### RNA‐seq and bioinformatic analysis

Purified RNA was subjected to library preparation and high‐throughput sequencing on Illumina Hiseq 4000 as Single‐Read 50 base reads following manufacturer's instructions. Sequenced samples were analyzed using nf‐core RNA‐seq pipeline v1.3 (Ewels *et al*, 2020). In brief, after a quality control and trimming of the samples, the sequences were aligned to the reference human genome (UCSC hg19 human genome) using HISAT2 (Kim *et al*, 2019). The identification and quantification of transcripts were performed using feature Counts from Subread package (Liao *et al*, 2014). For identification of transcripts, a gene has been considered transcribed in our RNA‐seq data if, at least, it has a coverage equal to or higher than 1 and a TPM (transcript per million reads) cutoff value of 4. We use the definition of coverage as the number of reads per base of the gene. DEG analysis were done using DeSeq2 (Love *et al*, 2014). Differential expressed gene (DEG) analysis were done using DeSeq2 (Love *et al*, 2014). Sequence and processed data had been submitted to National Center for Biotechnology Gene Expression Omnibus with GEO accession number GSE179074 and GSE195663.

### ATAC‐seq, ChIP‐seq, Chem‐seq, and BioChIP

For ATAC‐seq, “ATAC‐Seq Services Genome‐wide profiles of open chromatin regions from < 100,000 cells” from active motif was used. DMS‐53 cells were seeded in 15‐cm dishes and grown to subconfluency before treatment with 50 nM lurbinectedin. Cells were then cross‐linked with 1% of PFA and quenched with 0.125 M Glycine. Cell pellets were then washed in ice cold PBS before processing. For BioChIP, cross‐linked cells were permeabilized with cytonin (Active Motif) for 30 min at RT. After washing with PBS, terminal deoxynucleotidyl transferase (TdT) reaction was performed using Biotin‐16‐dUTP (Roche) and 60 units of recombinant enzyme rTdT (Promega). TdT reaction was then stopped with stop buffer (Chemicon International) for 15 min at RT. After washing with PBS, the samples were sonicated and immunoprecipitated using anti‐Biotin antibodies and treated as described in the ChIP protocol. Pellets were then lysed in cytosolic buffer (25 mM HEPES pH 7.8, 1.5 mM MgCl_2_, 10 mM KCl, 0.5% NP‐40, 1 mM DTT) to remove the cytosolic fraction. After spinning, isolated nuclei were lysed in SDS lysis buffer (0.1% SDS, 10 mM EDTA, 50 mM Tris HCl pH 7.5) and sonicated. 70 µg of chromatin fragmented up to 500 bp was then used for ChIP. Chromatin was diluted in Dilution Buffer (1% Triton, 2 mM EDTA, 20 mM Tris HCl pH 7.5, 150 mM NaCl) and incubated with 5 µg of the respective antibodies (Recombinant Anti‐NeuroD1 antibody [EPR4008] (Abcam ab109224), Anti‐MASH1/Achaete‐scute homolog 1 (Abcam ab211327), Mouse Anti‐RNAPII 7c2 (IGBMC Antibody facility; Santamaria Nunez *et al*, 2016), Anti‐Phospho‐Serine 2 RNAPII (Abcam ab5095), γH2AX (Abcam ab2893), Anti‐Biotin antibody (Abcam ab53494), and Histone H3K27Ac antibody (pAb) (Active motif) overnight at 4°C. Antigen–antibody complexes were then isolated with Protein A/G Sepharose beads (Pierce) for 2 h at 4°C. Beads containing the immunoprecipitated proteins were then washed in Low Salt Washing Buffer (1% Triton, 2 mM EDTA, 20 mM Tris HCl pH 7.5, 150 mM NaCl, 0.1% SDS), High salt Washing Buffer (1% Triton, 2 mM EDTA, 20 mM Tris HCl pH 7.5, 500 mM NaCl, 0.1% SDS), and TE buffer (100 mM Tris HCl pH 7.5, 10 mM EDTA). Immunoprecipitated chromatin was then eluted from the beads by incubation at 65°C for 30 min in Elution Buffer (1% SDS, 100 mM NaHCO_3_). Eluted complexes and respective inputs were then de‐cross‐linked with Proteinase K (50 µg/ml) at 65°C overnight and DNA purified with phenol–chloroform extraction (Drane *et al*, 2004; Le May *et al*, 2010). For Chem‐seq, DMS‐53 cells were incubated with Bio‐lur for the respective time points. Purified DNA was subjected to library preparation and high‐throughput sequencing on Illumina Hiseq 4000 as Single‐Read 50 base reads following the manufacturer's instructions.

### ChIP‐seq and chem‐seq bioinformatic analysis

Sequenced samples were analyzed using nf‐core ChIP‐seq pipeline v1.1.0 (Ewels *et al*, 2020). In brief, after a quality control and trimming of the samples, the sequences were aligned to the reference human genome (UCSC hg19 human genome) using Burrows‐Wheeler Aligner (BWA v0.7.17‐r11889; Li & Durbin, 2009). Peak calling was performed using MACS2 (Zhang *et al*, 2008) with default options, providing input chromatin data as control. Output BED files were then used for annotation. Peak profiles and heatmap plots were generated using scripts from package deepTools2 (Ramirez *et al*, 2016). Homer package (Heinz *et al*, 2010) were used to compute genomic distribution of peaks using default settings. For annotation of peaks on hg19, Homer package was used (annotatePeaks.pl <peak/BED file> <genome> > <output file>). Resulting output tables of annotated peaks were then by filtered by “PROMOTER‐TSS.” The same software was used to generate overlaps between gene names. Custom genomic annotation for Bio‐lur has been calculated by customizing the GRCh37.87 annotated‐promoter at −1 kb/+1 kb from TSS. The custom definition for Promoter was done filtering “distance to TSS” column by the limits selected. The custom annotation considers both customized values and ENSEMBL GRCh37.87 annotated promoters that were not included in the selected range (i.e., with a distance more than −/+1 kb from TSS).

Ascl1 and Neurod1 motif were obtained HOMER Motif Enrichment Analysis. Triplet motif files were generated using HOMER suite tools for each of the sequences. Motifs were then searched into −/+1/2 kb around Chem‐seq Promoter‐located peaks and random regions of similar size from hg19 genome promoters as background reference and their presence in 10‐bp genomic fragments were counted and a score were calculated for all the regions of interest using Homer suite v4.1. Sequence and processed data had been submitted to National Center for Biotechnology Gene Expression Omnibus with GEO accession number GSE179074 and GSE195663.

### Plots and venn diagrams

GraphPad Prism 9 software was used to generate all plots and IC_50_ values by iterative nonlinear curve fitting and perform statistical analysis. The area‐proportional Venn diagrams were drawn based on overlaps of annotated ChIP‐seq Peaks or RNA‐seq DESEQ2 gene lists generated by BioInforX (http://apps.bioinforx.com/bxaf7c/app/venn/app_overlap.php).

## Author contributions

FCos, JME, JFML, and EMG‐M conceived and designed the experiments. FCos, MMD, GSN, and CMGR carried out the experiments. FCoi, EC, RR, and T‐KL contributed reagents, materials, analysis tools and very fruitful discussion. FCos, JID‐H, JDP, EMG‐M, JFML, and JME analyzed the data. FCos, JME, JFML, and EMG‐M wrote the manuscript. All authors reviewed the final manuscript.

## Disclosure and competing interests statement

The authors declare that they have no conflict of interest.

JME is an editorial advisory board/EMBO Member. This has no bearing on the editorial consideration of this article for publication.

Our laboratory has a longstanding collaboration with Pharma Mar and has received partial research support from them. This work aimed to understand the mechanism of action activators overexpressed in SCLC cells and the mechanism of DNA binders that block transcriptional process. We found absolutely necessary to work with highly advanced chemists such as those working in the Pharma Mar company who were designing drugs that could specifically targets certain DNA motifs. MMD., GSN, JID‐H, JDP, JFML, and EMGM are employees of Pharma Mar SA (Madrid, Spain). FCos and CMGR were partly financed by Pharma Mar SA. Data and materials availability: lurbinectedin is available from Pharma Mar for noncommercial use under an MTA. All relevant data are included within this manuscript and all materials other than lurbinectedin are readily available via the links provided within the manuscript and upon request from Pharma Mar SA.

## Supporting information



Appendix

Expanded View Figures PDF

Table EV1

Table EV2

Source Data for Expanded View

Source Data for Figure 1

Source Data for Figure 3

Source Data for Figure 4

Source Data for Table 1

Source Data for Table 2

## Data Availability

All NGS data are available at Gene Expression Omnibus under GSE179074 (https://www.ncbi.nlm.nih.gov/geo/query/acc.cgi?acc=GSE179074) and GSE195663 (https://www.ncbi.nlm.nih.gov/geo/query/acc.cgi?acc=GSE195663).
