# Supplementary material for: Promoters of ASCL1‐ and NEUROD1‐dependent genes are specific targets of lurbinectedin in SCLC cells
Source: EMBO Mol Med. 2022 Mar 9;14(4):e14841. doi: 10.15252/emmm.202114841 (PMC8988166; doi:10.15252/emmm.202114841)
Supplement: Supplementary file 1 — Appendix [file EMMM-14-e14841-s005.pdf]

Appendix

Promoters of ASCL1- and NEUROD1-dependent genes are specific targets of lurbinectedin in SCLC cells.

Federico Costanzo et al.,

Table of content:

- Appendix Figure S1: (A-D) ATAC-seq and Chip-seq genome track of ASCL1/NEUROD1, RNAPII and Bio-Lur on *ASCL1, BCL2; INSM1* and *MYB*, in untreated (dark blue) and in lurbinectedin treated (green) conditions, respectively.

- Appendix Figure S2: Venn diagram of downregulated genes in DMS-53 after lurbinectedin, siNEUROD1 and siASCL1 treatments.

- Appendix Figure S3: (A)Venn diagram of genes expression in DMS-53, NCI-H510A and NCI-H82 cells. (B-C) Venn diagram of genes targeted by ASCL1 (B) and NEUROD1 (C) in DMS-53, NCI-H510A and NCI-H82 cells.

- Appendix Figure S4: ASCL1 and NEUROD1-dependent genes in DMS-53, NCI-H510A and NCI-H82 cells.

- Appendix Figure S5: Statistical analyses for Figure 4A, 4C, 5A-E, EV5B and EV5D

Appendix Figure S1

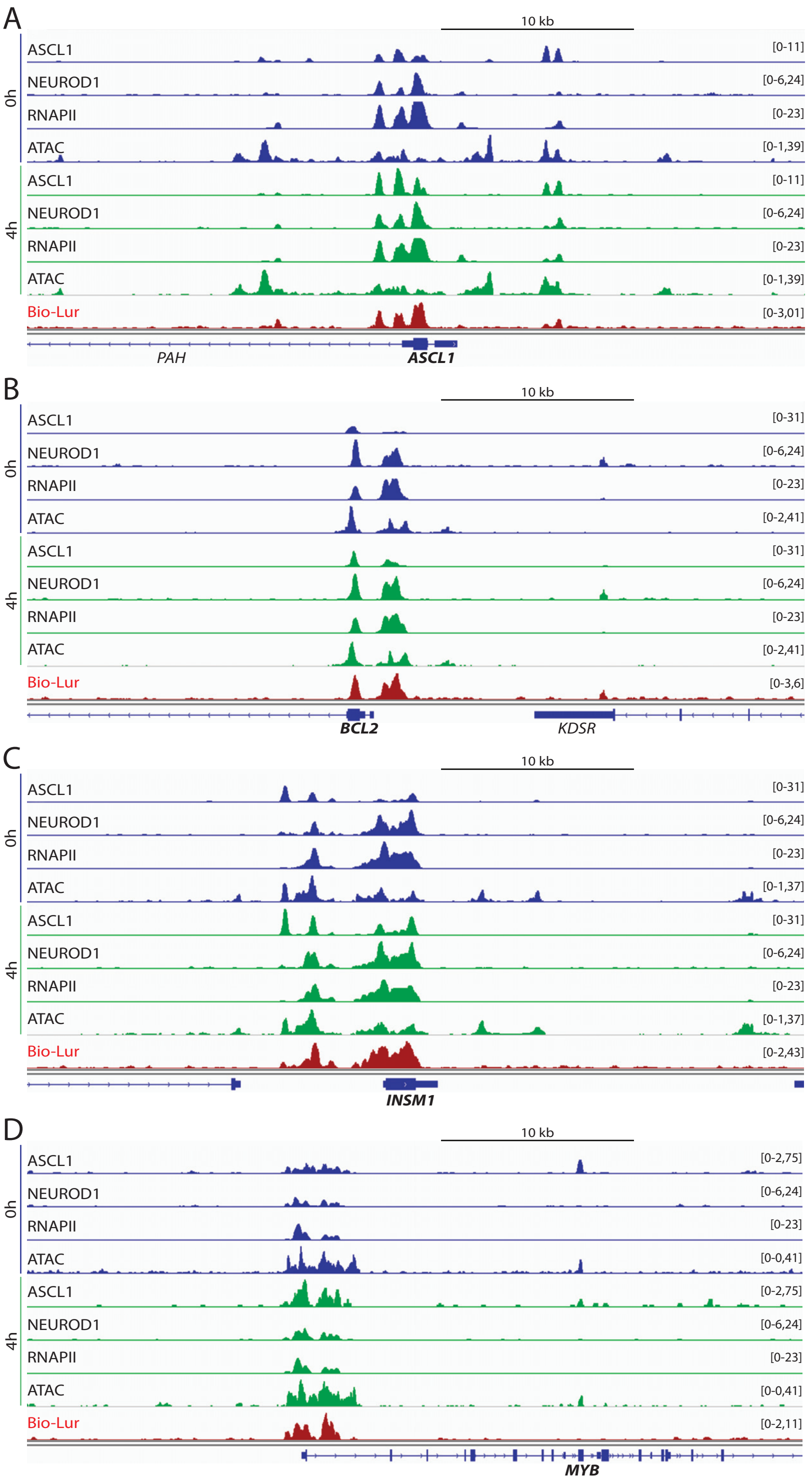

Chip-seq genome track of a second set of experiment showing ASCL1/NEUROD1, RNAPII, ATAC and H3K27Ac on *ASCL1* (A), *BCL2* (B), *INSM1*(C) and *MYB* (D) loci in untreated (dark blue) and in lurbinectedin treated (green) conditions, respectively. The presence of lurbinectedin (Bio-Lur, red) is also shown.

Appendix Figure S2

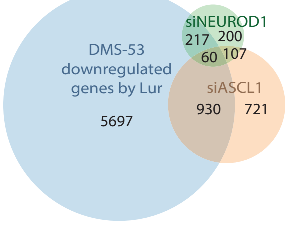

Venn diagrams showing the overlap between genes downregulated after 50nM treatment with lurbinectedin (Blue circle) and genes downregulated after silencing either ASCL1 (orange circle) or Neurod1 (green Circle).

Appendix Figure S3

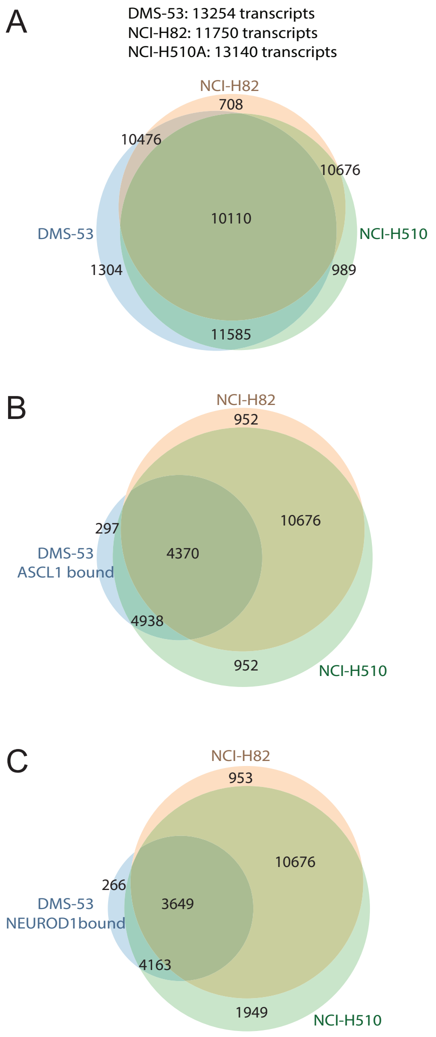

Expressed genes shared between DMS53, NCI-H82, and NCI H510A in untreated conditions (A) and targeted either by ASCL1 (B) or NEUROD1 (C). The number of transcripts from NCI-H82 and NCI-H510A cell lines was defined with the same parameters used for DMS-53 in Figure 1 (i.e. coverage equal to or higher than 1 and a TPM cut off value of 4).

Appendix Figure S4

A	DMS-53 down + NCI-H82 down	3701
B	NCI-H82 down + NCI-H510A down	4053
C	DMS-53 down + NCI-H510A down	4399
D	DMS-53 down + NCI-H510A down + NCI-H82 down	3374
E	ASCL1 ChIPseq	8131
F	NEUROD1 ChIPseq	7329

A + E	1671
A + F	1408
A + E + F	1096

B + E	1853
B + F	1607
B + E + F	1244

C + E	1957
C + F	1612
C + E + F	1241

D + E	1523
D + F	1289
D + E + F	998

Overlapping and comparison of the ASCL1 and NEUROD1 targeted genes and downregulated genes between the DMS53, NCI-H82, and NCI H510A cell lines after 50nM lurbinectedin treatment. Promoter-TSS annotated ChIPseq datasets. Letters corresponding to respective conditions are shown in the left. Overlap between different conditions are sown as additional tables below.

Appendix Figure S5

Statistical analyses for Figure 4A, 4C, 5A-E, EV5B and EV5D.

Figure 4A

Two-way RM ANOVA

ANOVA table	SS	DF	MS	F (DFn, DFd)	P value
Row Factor x Column Factor	0,1778	8	0,02223	F (8, 18) = 0,4541	P=0,8721
Row Factor	3,295	8	0,4119	F (8, 18) = 1,143	P=0,3832
Column Factor	4,261	1	4,261	F (1, 18) = 87,05	P<0,0001
Subject	6,489	18	0,3605	F (18, 18) = 7,365	P<0,0001
Residual	0,881	18	0,04895		

Šídák's multiple comparisons test	Mean Diff,	95,00% CI of diff,	Below threshold?	Summary	Adjusted P Value
**Ctrl - Lur**					
A549	0,6837	0,1168 to 1,251	Yes	*	0,0121
NCI-H460	0,7192	0,1523 to 1,286	Yes	**	0,0078
IMR90	0,3182	-0,2487 to 0,8851	No	ns	0,5932
NCI-H146	0,6456	0,07872 to 1,212	Yes	*	0,0194
NCI-H82	0,6042	0,03734 to 1,171	Yes	*	0,032
DMS-53	0,5056	-0,06130 to 1,072	No	ns	0,1019
NCI-H510	0,5268	-0,04006 to 1,094	No	ns	0,0799
NCI-H526	0,4825	-0,08440 to 1,049	No	ns	0,1318
SHP-77	0,5703	0,003372 to 1,137	Yes	*	0,048

Figure 4C

Unpaired t test			
P value	<0,0001	Mean of column A	46,15
P value summary	****	Mean of column B	18,55
Significantly different (P < 0.05)?	Yes	Difference between means (B - A) ± SEM	-27,60 ± 1,514
One- or two-tailed P value?	Two-tailed	95% confidence interval	-30,58 to -24,63
t, df	t=18,23, df=719	R squared (eta squared)	0,3161

**Sample size, column Ctrl**	508	F	Dfn	Dfd
**Sample size, column Lur**	213	4,495	507	212

Figure 5A

Carboplatin	A549	NCIH460	DMS53	NCIH82	NCIH510A
**log(inhibitor) vs. response - Variable slope (four parameters)**					
**Best-fit values**					
Bottom	~ -25871	~ -68331	-316,9	~ -1371	~ -62140
Top	87,62	100,3	109,5	102,3	122,7
LogIC50	~ -1,736	~ 4,694	-2,578	~ -2,300	~ 6,884
HillSlope	-1,395	~ -0,3490	-0,5551	-1,108	~ -0,2607
IC50	~ 0,01838	~ 49428	0,002645	~ 0,005009	~ 7653588
Span	~ 25959	~ 68431	426,4	~ 1473	~ 62262
**Std. Error**					
Bottom	~ 11367900	~ 36267963	667,2	~ 31783	~ 44500781
Top	1,462	7,46	2,882	1,206	28,37
LogIC50	~ 137,9	~ 665,0	1,815	~ 9,387	~ 1206
HillSlope	0,9096	~ 0,1991	0,1586	0,5395	~ 0,3307
Span	~ 11367901	~ 36267969	669,3	~ 31784	~ 44500807
**95% CI (asymptotic)**					
Bottom	(Very wide)	(Very wide)	-1634 to 1000	(Very wide)	(Very wide)
Top	84,73 to 90,50	85,00 to 115,7	103,8 to 115,2	99,86 to 104,7	65,85 to 179,5
LogIC50	(Very wide)	(Very wide)	-6,161 to 1,006	(Very wide)	(Very wide)
HillSlope	-3,192 to 0,4031	(Very wide)	-0,8682 to -0,2419	-2,180 to -0,03503	(Very wide)
IC50	???	???	6,900e-007 to 10,14	???	???
Span	(Very wide)	(Very wide)	-895,1 to 1748	(Very wide)	(Very wide)
**Goodness of Fit**					
Degrees of Freedom	146	26	165	86	56
R squared	0,7368	0,9633	0,8571	0,8362	0,8463
Sum of Squares	24740	808,1	24158	4986	11712
Sy.x	13,02	5,575	12,1	7,614	14,46
**Number of points**					
# of X values	240	240	240	240	240
# Y values analyzed	150	30	169	90	60

Figure 5B

Etoposide	A549	NCIH460	DMS53	NCIH82	NCIH510A
**log(inhibitor) vs. response - Variable slope (four parameters)**					
**Best-fit values**					
Bottom	~ -151,3	-1,219	53,42	-745146	-1,767
Top	~ 3857	~ 55020	117,1	97,05	130,7
LogIC50	~ -30,52	~ -20,96	-5,927	9,099	-6,004
HillSlope	~ -0,05055	~ -0,2044	-0,7447	-0,3436	-0,7475
IC50	~ 3,037e-031	~ 1,102e-021	0,000001184	1255131688	9,904E-07
Span	~ 4008	~ 55022	63,67	745243	132,4
**Std. Error**					
Bottom	~ 1668	34	4,997		3,338
Top	~ 547880	~ 51203074	9,076		6,902
LogIC50	~ 1548	~ 2002	0,2059		0,07373
HillSlope	~ 0,7659	~ 0,3319	0,2716		0,09339
Span	~ 549543	~ 51203106	12,59		9,2
**95% CI (asymptotic)**					
Bottom	(Very wide)	-71,10 to 68,67	43,55 to 63,28		-8,454 to 4,920
Top	(Very wide)	(Very wide)	99,16 to 135,0		116,8 to 144,5
LogIC50	(Very wide)	(Very wide)	-6,333 to -5,520		-6,152 to -5,856
HillSlope	(Very wide)	(Very wide)	-1,281 to -0,2085		-0,9346 to -0,5604
IC50	???	???	4,644e-007 to 3,019e-006		7,049e-007 to 1,392e-006
Span	(Very wide)	(Very wide)	38,82 to 88,52		114,0 to 150,9
**Goodness of Fit**					
Degrees of Freedom	146	26	166		56
R squared	0,864	0,9406	0,5616		0,9702
Sum of Squares	16427	1050	57539		3389
Sy.x	10,61	6,353	18,62		7,779
**Number of points**					
# of X values	240	240	240	240	240
# Y values analyzed	150	30	170	90	60

Figure 5C

Topotecan	A549	NCIH460	DMS53	NCIH82	NCIH510A
**log(inhibitor) vs. response - Variable slope (four parameters)**					
**Best-fit values**					
Bottom	~ -121383	19,36	~ -3,169	~ -7031	34,69
Top	107,5	102,7	~ 1297	~ 133,1	105,6
LogIC50	~ 8,848	-7,048	~ -16,24	~ 11,89	-6,573
HillSlope	~ -0,2332	-2,071	~ -0,1317	~ -0,1166	-0,6135
IC50	~ 704494874	8,945E-08	~ 5,745e-017	~ 780328278226	2,675E-07
Span	~ 121491	83,37	~ 1301	~ 7164	70,96
**Std. Error**					
Bottom	~ 191885000	1,419	~ 105,1	~ 4165887	11,14
Top	19,86	2,405	~ 35917	~ 239,7	13,58
LogIC50	~ 2958	0,03187	~ 120,4	~ 2330	0,2972
HillSlope	~ 0,2585	0,2889	~ 0,4054	~ 1,053	0,3293
Span	~ 191885018	2,958	~ 36020	~ 4166123	22,62
**95% CI (asymptotic)**					
Bottom	(Very wide)	16,54 to 22,18	(Very wide)	(Very wide)	12,38 to 57,00
Top	68,05 to 147,0	97,94 to 107,5	(Very wide)	(Very wide)	78,45 to 132,8
LogIC50	(Very wide)	-7,112 to -6,985	(Very wide)	(Very wide)	-7,168 to -5,977
HillSlope	(Very wide)	-2,646 to -1,497	(Very wide)	(Very wide)	-1,273 to 0,04615
IC50	???	7,731e-008 to 1,035e-007	???	???	6,794e-008 to 1,054e-006
Span	(Very wide)	77,49 to 89,25	(Very wide)	(Very wide)	25,65 to 116,3
Goodness of Fit					
**Degrees of Freedom**	86	86	86	86	56
R squared	0,8694	0,9402	0,8635	0,5399	0,6994
Sum of Squares	7317	6956	6696	20382	11893
Sy.x	9,224	8,994	8,824	15,39	14,57
**Number of points**					
# of X values	240	240	240	240	240
# Y values analyzed	90	90	90	90	60

Figure 5D

Lurbinectedin	A549	NCIH460	DMS53	NCIH82	NCIH510A
**log(inhibitor) vs. response - Variable slope (four parameters)**					
**Best-fit values**					
Bottom	19,41	8,215	1,52	16,25	9,723
Top	93,15	97,41	105,1	101,4	94,69
LogIC50	-8,822	-8,975	-8,674	-8,879	-9,786
HillSlope	-1,752	-1,447	-0,9883	-1,66	-1,212
IC50	1,506E-09	1,059E-09	2,116E-09	1,322E-09	1,637E-10
Span	73,75	89,19	103,6	85,1	84,97
**Std. Error**					
Bottom	3,12	5,991	15,18	5,221	3,113
Top	1,222	2,579	2,863	2,138	3,516
LogIC50	0,04165	0,07574	0,1637	0,06351	0,06836
HillSlope	0,2474	0,3141	0,2434	0,3402	0,2177
Span	3,549	7,051	16,58	6,007	5,312
**95% CI (asymptotic)**					
Bottom	13,26 to 25,55	-3,651 to 20,08	-28,54 to 31,58	5,791 to 26,71	3,571 to 15,88
Top	90,74 to 95,56	92,30 to 102,5	99,47 to 110,8	97,07 to 105,6	87,74 to 101,6
LogIC50	-8,904 to -8,740	-9,125 to -8,825	-8,999 to -8,350	-9,006 to -8,752	-9,921 to -9,651
HillSlope	-2,240 to -1,265	-2,069 to -0,8249	-1,470 to -0,5063	-2,341 to -0,9785	-1,642 to -0,7818
IC50	1,247e-009 to 1,820e-009	7,499e-010 to 1,496e-009	1,003e-009 to 4,464e-009	9,860e-010 to 1,772e-009	1,200e-010 to 2,235e-010
Span	66,76 to 80,74	75,23 to 103,2	70,77 to 136,5	73,07 to 97,14	74,47 to 95,47
**Goodness of Fit**					
Degrees of Freedom	236	116	116	56	146
R squared	0,8221	0,7959	0,7846	0,9015	0,8122
Sum of Squares	39963	35262	32194	6838	40072
Sy.x	13,01	17,44	16,66	11,05	16,57
**Number of points**					
# of X values	240	240	240	240	240
# Y values analyzed	240	120	120	60	150

Figure 5E

Cisplatin	A549	NCIH460	DMS53	NCIH82	NCIH510A
**log(inhibitor) vs. response - Variable slope (four parameters)**					
**Best-fit values**					
Bottom	6,276	-0,4616	9,243	0,5795	0,932
Top	94,5	102,6	100,1	99,89	94,87
LogIC50	-4,791	-5,226	-4,125	-4,755	-4,076
HillSlope	-1,942	-1,416	-2,248	-2,788	-3,063
IC50	0,00001618	0,000005938	0,00007501	0,00001757	0,00008404
Span	88,22	103	90,86	99,31	93,94
**Std. Error**					
Bottom	1,931	1,098	1,561	0,901	5,403
Top	1,272	1,05	0,4401	0,6378	1,765
LogIC50	0,02686	0,01768	0,01304	0,009778	0,04206
HillSlope	0,2023	0,07424	0,1172	0,1603	0,5719
Span	2,448	1,674	1,68	1,147	5,774
**95% CI (asymptotic)**					
Bottom	2,437 to 10,11	-2,644 to 1,721	6,139 to 12,35	-1,212 to 2,371	-9,808 to 11,67
Top	91,97 to 97,03	100,5 to 104,7	99,23 to 101,0	98,62 to 101,2	91,36 to 98,38
LogIC50	-4,844 to -4,738	-5,262 to -5,191	-4,151 to -4,099	-4,775 to -4,736	-4,159 to -3,992
HillSlope	-2,345 to -1,540	-1,563 to -1,268	-2,481 to -2,015	-3,106 to -2,469	-4,200 to -1,926
IC50	1,431e-005 to 1,830e-005	5,476e-006 to 6,438e-006	7,066e-005 to 7,962e-005	1,680e-005 to 1,837e-005	6,932e-005 to 0,0001019
Span	83,36 to 93,09	99,71 to 106,4	87,52 to 94,20	97,03 to 101,6	82,46 to 105,4
**Goodness of Fit**					
Degrees of Freedom	86	86	86	86	86
R squared	0,9612	0,9887	0,99	0,9915	0,8614
Sum of Squares	5079	1891	902,4	1474	16048
Sy.x	7,685	4,689	3,239	4,14	13,66
**Number of points**					
# of X values	240	240	240	240	240
# Y values analyzed	90	90	90	90	90

Figure EV5B

ASCL1 (a.u.)

**Unpaired t test**	
P value	<0,0001
P value summary	****
Significantly different (P < 0.05)?	Yes
One- or two-tailed P value?	Two-tailed
t, df	t=54,11, df=2205

Mean of column A	44,34
Mean of column B	21,94
Difference between means (B - A) ± SEM	22,40 ± 0,4140
95% confidence interval	21,59 to 23,21
R squared (eta squared)	0,5704

**Sample size, column Ctrl**	994
**Sample size, column Lur**	1213

F	Dfn	Dfd
2,197	993	1212

Figure EV5D

Viable Cells

Unpaired t test

P value	0,0034	Mean of column A	62,9
P value summary	**	Mean of column B	48
Significantly different (P < 0.05)?	Yes	Difference between means (B - A) ± SEM	-14,90 ± 2,389
One- or two-tailed P value?	Two-tailed	95% confidence interval	-21,53 to - 8,267
t, df	t=6,237, df=4	R squared (eta squared)	0,9068

**Sample size, column Ctrl**	3	F	Dfn	Dfd
**Sample size, column Lur**	3	4,47	2	2

Early Apoptotic Cells

Unpaired t test

P value	0,0906	Mean of column A	0,1933
P value summary	ns	Mean of column B	1,177
Significantly different (P < 0.05)?	No	Difference between means (B - A) ± SEM	0,9833 ± 0,4429
One- or two-tailed P value?	Two-tailed	95% confidence interval	-0,2463 to 2,213
t, df	t=2,220, df=4	R squared (eta squared)	0,552

**Sample size, column Ctrl**	3	F	Dfn	Dfd
**Sample size, column Lur**	3	193	2	2

Late Apoptotic Cells

Unpaired t test

P value	0,5865	Mean of column A	7,49
P value summary	ns	Mean of column B	9,19
Significantly different (P < 0.05)?	No	Difference between means (B - A) ± SEM	1,700 ± 2,878
One- or two-tailed P value?	Two-tailed	95% confidence interval	-6,291 to 9,691
t, df	t=0,5906, df=4	R squared (eta squared)	0,08021

**Sample size, column Ctrl**	3	F	Dfn	Dfd
**Sample size, column Lur**	3	3,839	2	2
