# Supplementary material for: Promoters of ASCL1‐ and NEUROD1‐dependent genes are specific targets of lurbinectedin in SCLC cells
Source: EMBO Mol Med. 2022 Mar 9;14(4):e14841. doi: 10.15252/emmm.202114841 (PMC8988166; doi:10.15252/emmm.202114841)
Supplement: Supplementary file 2 — Expanded View Figures PDF [file EMMM-14-e14841-s004.pdf]

Expanded View Figures

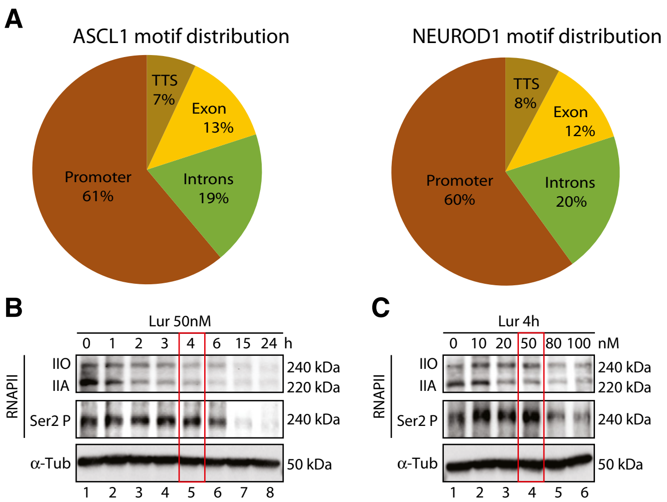

Figure EV1. Distribution of ASCL1 and NEUROD1 cognate sequences and optimization of Lurbinectedin treatment.

- A Venn diagram showing the Genomic Distribution of ASCL1 and NEUROD1 motif (in percentage) on transcripts from RNA-seq. ASCL1 and NEUROD1 motif occurrence has been calculated on custom promoter regions (−1 kb/+1 kb) on transcripts having at least 1 motif in their promoter region.
- B Western blot showing time-dependent hypo- (IIA) or hyper (IIO)-phosphorylated RNAPII (upper panel) and phosphorylated RNAPII at Ser2 (Ser2-P) (middle panel) protein level decrease in DMS-53 cell lines from 0 h to 24 h after 50 nM or lurbinectedin treatment. Red box (lane 5) highlights the time-point of RNAPII significant decrease. α-Tubulin (α-Tub) is shown as loading control. For all the experiments, cells were collected 4 h after 50 nM lurbinectedin treatment.
- C Western blot showing dose dependency of RNAPIIA or RNAPIIO) (upper panel) and phosphorylated RNAPII at Ser2 (Ser2-P) (lower Panel) protein level decrease in DMS-53 cell lines at different lurbinectedin concentrations at 4 h. Red box (lane 4) highlights the optimal concentration for RNAPII significant decrease. α-Tubulin (α-Tub) is shown as loading control. For all the experiments, cells were collected 4 h after 50 nM lurbinectedin treatment.

Source data are available online for this figure.

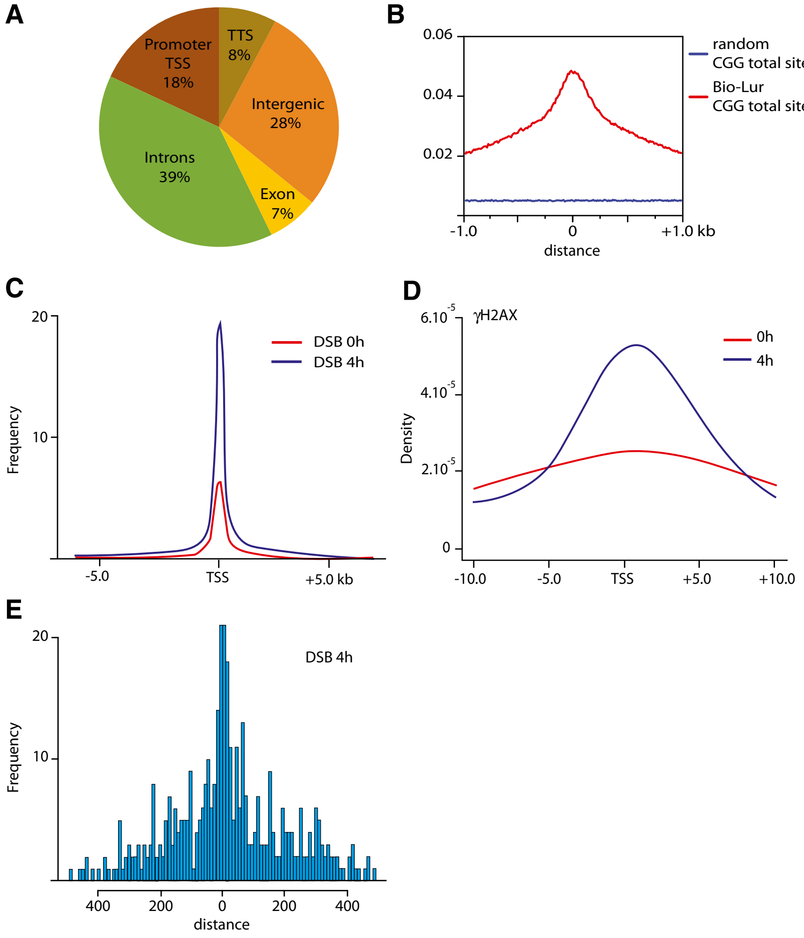

Figure EV2. Lurbinectedin induces DNA breaks in its surrounding around gene TSS.

- A Venn diagram showing the distribution of lurbinectedin-annotated Peaks (in percentage) all over the genome (hg19). Peak annotation has been performed by customizing the promoter region from −1 kb up to +1 kb around TSS, in order to comprise Lur peaks downstream from TSS.
- B Enrichment of CGG motifs (red) into −/+1/2 kb around lurbinectedin Promoter-located peaks and random regions of similar size (blue) from hg19 genome promoters as background reference and their presence in 10 bp were counted and scored.
- C, D (C) BrdUTP-ChIP-seq representing DNA breaks (DSB) and (D) ChIP-seq experiments indicate γH2AX localization. Lines representing ChIP-seq profile at 0 h (red) and 4 h (blue) after lurbinectedin treatment.
- E Histogram of the distance (in bp) between the DSBs (BrdUTP ChIP-seq) and the TSS at 4 h after lurbinectedin treatment.

© 2022 The Authors

© 2022 The Authors

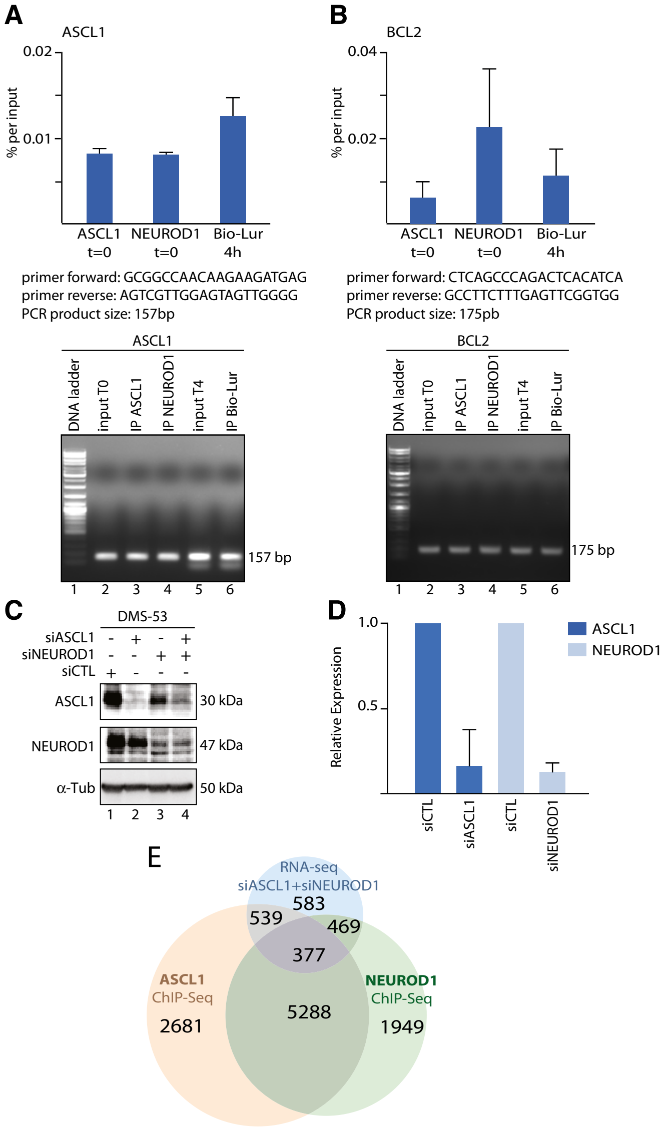

Figure EV3. Identification of ASCL1- and NEUROD1-dependent genes in ChIp-seq/Bio-Lur, Silencing of ASCL1/NEUROD1 and evaluation of ASCL1/NEUROD1-dependent genes by RNA-seq.

A, B ChIP-qPCR showing the enrichment of ASCL1, NEUROD1, and Lur (Bio-Lur) at ASCL1 and BCL2 genes (upper panels) and the respective primer sequences, PCR product sizes, and specificity (lower panels showing Agarose signal at the respective amplicon sizes). Data are represented as mean ± SEM (n = 3 technical replicates).

C Western blot showing the silencing of both ASCL1 (lanes 2 and 4) and/or NEUROD1 (lanes 3 ad 4) in the DMS53 cell line as compared to nontargeting siRNA (siCTL, Lane 1). αtub is used as loading control.

D Histogram of qPCR showing the silencing of ASCL1 (Dark Blue) and NEUROD1 (Light Blue) in the DMS53 cell line as compared to nontargeting siRNA (siCTL,). Data (represented as mean ± SEM, n = 3 technical replicates) are shown as relative mRNA expression normalized to nontargeting siRNA (siCTL).

E Venn diagrams showing the overlap between genes downregulated in double silenced siASCL1 /siNEUROD1 DMS53 (blue circle) and ASCL1 and NEUROD1 ChIP-seq (orange and green circles, respectively).

Source data are available online for this figure.

© 2022 The Authors

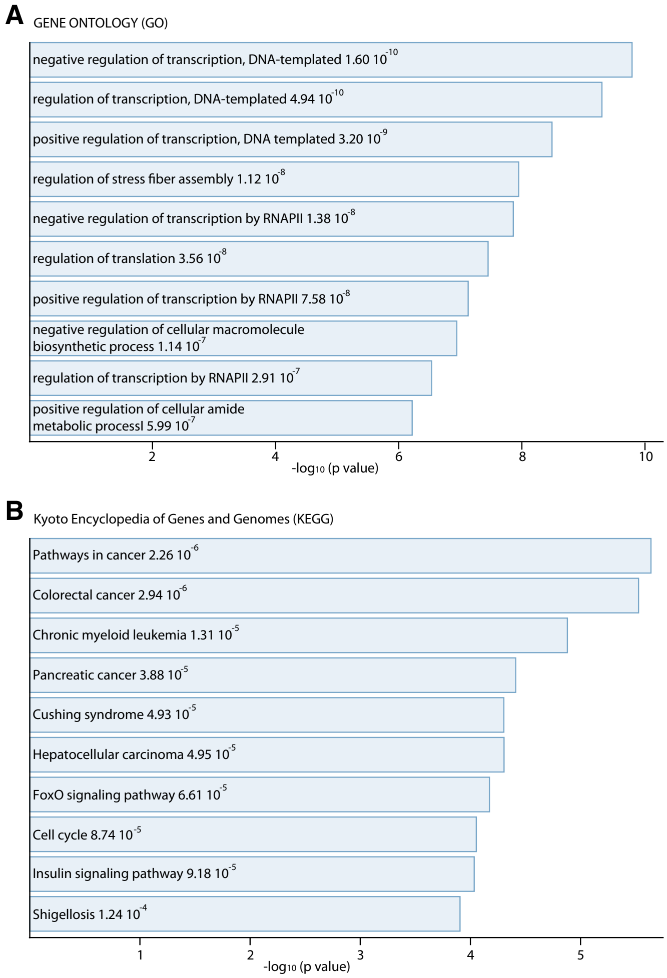

Figure EV4. Gene ontology pathway of ASCL1- and NEUROD1-dependent genes.

A, B Gene Ontology (A) and pathway analysis (B) of the intersection between Fig 1 B and C representing the most enriched and statistically significant terms according to GO Biological Processes and KEGG pathways. *P*-values are shown as $-\log_{10}$.

© 2022 The Authors

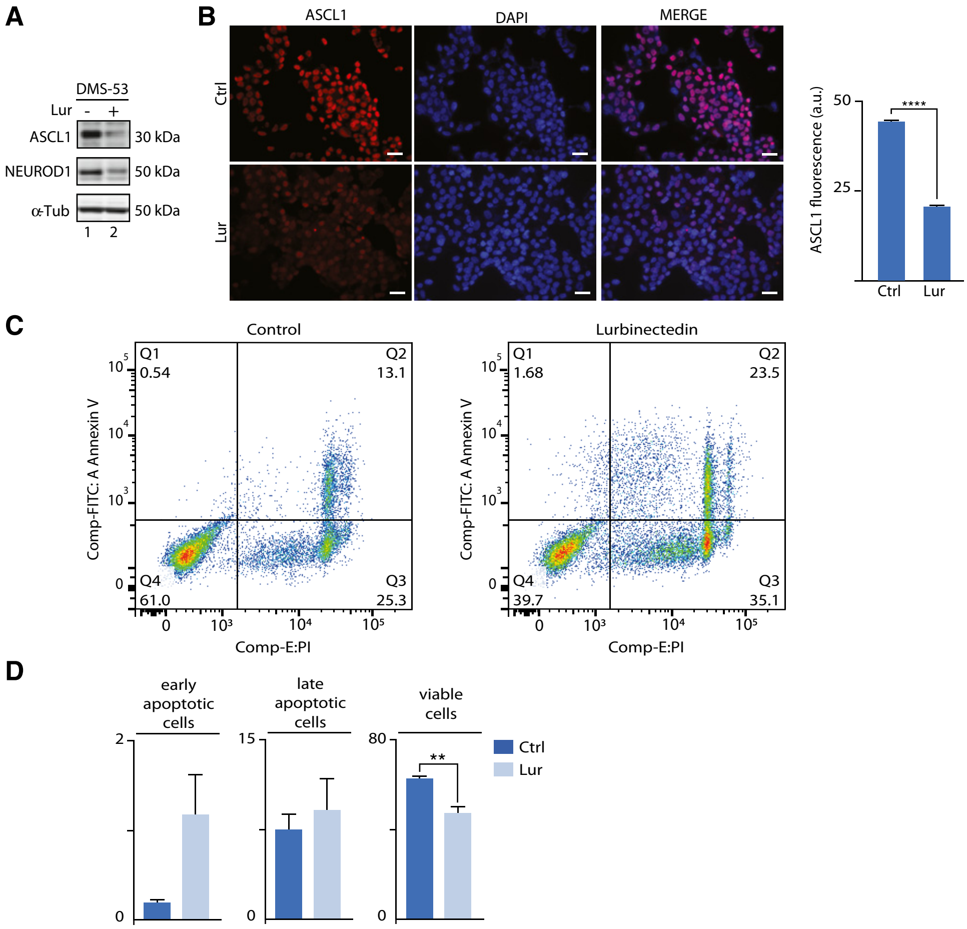

Figure EV5. ASCL1 and NEUROD1 expression in DMS-53 cell line before and after treatment with lurbinectedin.

A Western blot showing ASCL1 and NEUROD1 expression in DMS-53 cell line before (−) and after (+) lurbinectedin treatment. α-Tub is shown as loading control. Each blot is representative of at least three different experiments.

B Immunofluorescence showing the decrease in ASCL1 (red) after lurbinectedin treatment. DAPI (blue) was used to stain nuclei; scale bar is 30 μm. (right panel) Histogram showing the average fluorescence signal of ASCL1 before and after lurbinectedin treatment in arbitrary unit (a.u.). Data are presented as Mean ± SEM. ****$P \leq 0.0001$ as determined by Student's *t*-test ($n = 3$). Each assay is representative of at least three different experiments.

C Lurbinectedin triggers apoptosis in DMS-53 cells. Annexin V-FITC/PI analysis of apoptosis in DMS-53 cancer cells before (left panel) and after (right Panel) 50 nM lurbinectedin treatment. Cells were incubated with lurbinectedin for 24 h. The upper left panel (Q1) displays early apoptotic cells, whereas the lower right panel (Q3) represents late apoptotic cells. Lower left panel (Q4) represents viable cells.

D Histogram showing the percentage of early apoptotic, late apoptotic, and viable cells from Control- (Ctrl, dark blue) and lurbinectedin (Lur, light blue)-treated groups. Data are presented as Mean ± SEM. **$P \leq 0.0021$ as determined by Student's *t*-test ($n = 3$ technical replicates).

Source data are available online for this figure.
