# Supplementary material for: Promoters of ASCL1‐ and NEUROD1‐dependent genes are specific targets of lurbinectedin in SCLC cells
Source: EMBO Mol Med. 2022 Mar 9;14(4):e14841. doi: 10.15252/emmm.202114841 (PMC8988166; doi:10.15252/emmm.202114841)
Supplement: Supplementary file 3 — Table EV1 [file EMMM-14-e14841-s011.docx]

Table EV1

| **cell line** | **IC_50_ Lur** |
| --- | --- |
| A549 | 1,866.10^-9^ |
| NCI-H460 | 1,864.10^-9^ |
| NCI-H69 | 4,144.10^-10^ |
| NCI-H128 | 1,316.10^-9^ |
| NCI-H146 | 1,24.10^-10^ |
| NCI-H187 | 4,998.10^-10^ |
| NCI-H510A | 1,103.10^-10^ |
| NCI-H1105 | 6,839.10^-9^ |
| NCI-H1436 | 1,068.10^-9^ |
| DMS 153 | 1,3.10^-10^ |
| SHP-77 | 2,60.10^-9^ |
| NCI-H82 | 1,64.10^-9^ |
| NCI-H446 | 1,221.10^-9^ |
| NCI-H2171 | 4,894.10^-10^ |
| DMS-53 | 2,161.10^-9^ |
| DMS-273 | 3,61.10^-11^ |
| NCI-H345 | 3,535.10^-10^ |
| NCI-H841 | 8,614.10^-10^ |
| DM S114 | 1,436.10^-9^ |
| NCI-H209 | 1,044.10^-10^ |
| NCI-H211 | 3,738.10^-10^ |
| NCI-H378 | 2,966.10^-11^ |
| NCI-H526 | 2,249.10^-10^ |
| NCI-H1048 | 1,54.10^-10^ |
| DMS-79 | 1,21.10^-10^ |

**Table EV1:** IC_50_ values for SCLC and NSCLC cell lines treated with lurbinectedin.
