# Supplementary material for: Promoters of ASCL1‐ and NEUROD1‐dependent genes are specific targets of lurbinectedin in SCLC cells
Source: EMBO Mol Med. 2022 Mar 9;14(4):e14841. doi: 10.15252/emmm.202114841 (PMC8988166; doi:10.15252/emmm.202114841)
Supplement: Supplementary file 4 — Table EV2 [file EMMM-14-e14841-s007.docx]

Table EV2

| **ASCL1 ChIp-seq**  **(Augustyn et al)** | **Chem-seq.Lur** | **RNA-seq; Lur** |
| --- | --- | --- |
| ASCL1 | **+** | **+** |
| BCL2 | **+** | **+** |
| CACNA1A | **+** | **+** |
| CAMK1D | **+** | **+** |
| CAPS |  |  |
| CNGB1 | **+** |  |
| CRIP2 | **+** |  |
| DGCR2 |  | **+** |
| DGKB |  | **+** |
| DIRAS2 |  | **+** |
| DMPK | **+** |  |
| DOCK10 |  |  |
| DOK6 | **+** |  |
| DUSP6 |  |  |
| ECE1 |  |  |
| ERO1LB |  |  |
| ETS2 |  |  |
| FAM70B |  |  |
| FBP1 |  |  |
| FOS |  |  |
| FOXA2 | **+** |  |
| FOXC1 | **+** |  |
| GCA |  |  |
| GRP | **+** |  |
| ID2 | **+** |  |
| ID4 | **+** |  |
| INA | **+** | **+** |
| IRF2BP2 | **+** | **+** |
| ISG20 |  |  |
| KDM4B |  | **+** |
| KIAA0182 |  |  |
| KRT7 |  |  |
| KSR2 |  | **+** |
| LYPD1 |  |  |
| MAP6 | **+** |  |
| NAV1 | **+** | **+** |
| S6. |  |  |
| NKAIN2 | **+** |  |
| NPTX1 | **+** |  |
| NR0B2 |  | **+** |
| NUAK2 |  | **+** |
| PCNXL2 |  |  |
| PFKFB2 |  |  |
| PLXNA2 |  | **+** |
| PTPRN2 | **+** |  |
| RAB3B |  | **+** |
| RGS12 | **+** | **+** |
| RNF11 |  | **+** |
| RNF183 |  | **+** |
| RPS6KC1 |  | **+** |
| SCN2A |  |  |
| SCN3A |  | **+** |
| SEC11C | **+** |  |
| SEPW1 |  |  |
| SETBP1 | **+** | **+** |
| SH3BP4 | **+** | **+** |
| SLC36A4 | **+** | **+** |
| SLC6A17 |  |  |
| SMOC2 |  |  |
| SPPL2B | **+** |  |
| ST18 | **+** | **+** |
| SVIL |  |  |
| TMEM61 | **+** |  |
| TOX | **+** |  |
| TOX3 | **+** | **+** |
| TSGA10 | **+** | **+** |
| TTC13 | **+** | **+** |
| WASF2 | **+** | **+** |
| ZBTB20 | **+** | **+** |
| ZBTB40 |  | **+** |
| ZFHX3 |  | **+** |
| ZNF516 | **+** | **+** |

**Table EV2:** Overlap between ASCL1 Chem-seq targets (yellow) and RNA-seq downregulated genes (green) after lurbinectedin treatment, and the ASCL1 target genes from (Augustyn et al., 2014). Gene list is shown on Column 1. Successful overlap is highlighted with a (+) sign.
