# Supplementary material for: Promoters of ASCL1‐ and NEUROD1‐dependent genes are specific targets of lurbinectedin in SCLC cells
Source: EMBO Mol Med. 2022 Mar 9;14(4):e14841. doi: 10.15252/emmm.202114841 (PMC8988166; doi:10.15252/emmm.202114841)
Supplement: Supplementary file 11 [file EMMM-14-e14841-s009.pdf]

EMBO Molecular Medicine

Promoters of ASCL1- and NEUROD1-dependent genes are specific targets of lurbinectedin in SCLC cells

Federico Costanzo, Marta Martínez Diez, Gema Santamaría Núnez, Juan Ignacio Díaz-Hernandéz, Carlos Mario Genes Robles, Javier Diez Perez, Emmanuel Compe, Romeo Ricci, Tsai-Kun Li, Frédéric Coin, Juan Fernando Martínez Leal, Eva Maria Garrido-Martin, and Jean-Marc Egly
DOI: 10.15252/emmm.202114841

Corresponding author: Jean-Marc Egly (Jean-Marc.Egly@igbmc.fr)

Review Timeline:

Submission Date:	12th Jul 21
Editorial Decision:	3rd Aug 21
Revision Received:	17th Dec 21
Editorial Decision:	2nd Feb 22
Revision Received:	12th Feb 22
Editorial Decision:	15th Feb 22
Revision Received:	16th Feb 22
Accepted:	17th Feb 22

Editor: Lise Roth

Transaction Report:

(Note: With the exception of the correction of typographical or spelling errors that could be a source of ambiguity, letters and reports are not edited. Depending on transfer agreements, referee reports obtained elsewhere may or may not be included in this compilation. Referee reports are anonymous unless the Referee chooses to sign their reports.)

3rd Aug 2021

Dear Prof. Egly,

Thank you for submitting your work to EMBO Molecular Medicine. We have now heard back from the referees who agreed to evaluate your manuscript. As you will see below, the reviewers raise substantial concerns on your work, which unfortunately preclude its publication in EMBO Molecular Medicine in its current form.

Indeed, the reviewers find that the question addressed by the study is of potential (pre)clinical interest. However, they remain unconvinced that some of the major conclusions are sufficiently supported by the data. In particular, they mention that the NGS data should be accessible and functionally validated.

If you feel you can satisfactorily address this point and the other issues listed by the referees, you may wish to submit a revised version of your manuscript. Please attach a covering letter giving details of the way in which you have handled each of the points raised by the referees. A revised manuscript will once again be subject to review, and we cannot guarantee at this stage that the eventual outcome will be favorable.

Revised manuscripts should be submitted within three months of a request for revision; they will otherwise be treated as new submissions, except under exceptional circumstances in which a short extension is obtained from the editor.

When submitting your revised manuscript, please carefully review the instructions that follow below. We perform an initial quality control of all revised manuscripts before re-review; failure to include requested items will delay the evaluation of your revision.

We require:

1) A .docx formatted version of the manuscript text (including legends for main figures, EV figures and tables). Please make sure that the changes are highlighted to be clearly visible.

2) Individual production quality figure files as .eps, .tif, .jpg (one file per figure). For guidance, download the 'Figure Guide PDF' (https://www.embopress.org/page/journal/17574684/authorguide#figureformat).

3) A .docx formatted letter INCLUDING the reviewers' reports and your detailed point-by-point responses to their comments. As part of the EMBO Press transparent editorial process, the point-by-point response is part of the Review Process File (RPF), which will be published alongside your paper.

4) A complete author checklist, which you can download from our author guidelines (https://www.embopress.org/page/journal/17574684/authorguide#submissionofrevisions). Please insert information in the checklist that is also reflected in the manuscript. The completed author checklist will also be part of the RPF.

5) Please note that all corresponding authors are required to supply an ORCID ID for their name upon submission of a revised manuscript.

6) It is mandatory to include a 'Data Availability' section after the Materials and Methods. Before submitting your revision, primary datasets produced in this study need to be deposited in an appropriate public database, and the accession numbers and database listed under 'Data Availability'. Please remember to provide a reviewer password if the datasets are not yet public (see https://www.embopress.org/page/journal/17574684/authorguide#dataavailability).

In case you have no data that requires deposition in a public database, please state so in this section. Note that the Data Availability Section is restricted to new primary data that are part of this study.

7) For data quantification: please specify the name of the statistical test used to generate error bars and P values, the number (n) of independent experiments (specify technical or biological replicates) underlying each data point and the test used to calculate p-values in each figure legend. The figure legends should contain a basic description of n, P and the test applied. Graphs must include a description of the bars and the error bars (s.d., s.e.m.).

8) We would also encourage you to include the source data for figure panels that show essential data. Numerical data should be provided as individual .xls or .csv files (including a tab describing the data). For blots or microscopy, uncropped images should be submitted (using a zip archive if multiple images need to be supplied for one panel). Additional information on source data and instruction on how to label the files are available at
.

9) Our journal encourages inclusion of *data citations in the reference list* to directly cite datasets that were re-used and obtained from public databases. Data citations in the article text are distinct from normal bibliographical citations and should directly link to the database records from which the data can be accessed. In the main text, data citations are formatted as follows: "Data ref: Smith et al, 2001" or "Data ref: NCBI Sequence Read Archive PRJNA342805, 2017". In the Reference list, data citations must be labeled with "[DATASET]". A data reference must provide the database name, accession number/identifiers and a resolvable link to the landing page from which the data can be accessed at the end of the reference. Further instructions are available at .

10) We replaced Supplementary Information with Expanded View (EV) Figures and Tables that are collapsible/expandable online. A maximum of 5 EV Figures can be typeset. EV Figures should be cited as 'Figure EV1, Figure EV2" etc... in the text and their respective legends should be included in the main text after the legends of regular figures.

- For the figures that you do NOT wish to display as Expanded View figures, they should be bundled together with their legends in a single PDF file called *Appendix*, which should start with a short Table of Content. Appendix figures should be referred to in the main text as: "Appendix Figure S1, Appendix Figure S2" etc.

- Additional Tables/Datasets should be labeled and referred to as Table EV1, Dataset EV1, etc. Legends have to be provided in a separate tab in case of .xls files. Alternatively, the legend can be supplied as a separate text file (README) and zipped together with the Table/Dataset file.

See detailed instructions here:
.

11) The paper explained: EMBO Molecular Medicine articles are accompanied by a summary of the articles to emphasize the major findings in the paper and their medical implications for the non-specialist reader. Please provide a draft summary of your article highlighting
- the medical issue you are addressing,
- the results obtained and
- their clinical impact.

This may be edited to ensure that readers understand the significance and context of the research. Please refer to any of our published articles for an example.

12) For more information: There is space at the end of each article to list relevant web links for further consultation by our readers. Could you identify some relevant ones and provide such information as well? Some examples are patient associations, relevant databases, OMIM/proteins/genes links, author's websites, etc...

13) Every published paper now includes a 'Synopsis' to further enhance discoverability. Synopses are displayed on the journal webpage and are freely accessible to all readers. They include a short stand first (maximum of 300 characters, including space) as well as 2-5 one-sentences bullet points that summarizes the paper. Please write the bullet points to summarize the key NEW findings. They should be designed to be complementary to the abstract - i.e. not repeat the same text. We encourage inclusion of key acronyms and quantitative information (maximum of 30 words / bullet point). Please use the passive voice. Please attach these in a separate file or send them by email, we will incorporate them accordingly.

Please also suggest a striking image or visual abstract to illustrate your article as a PNG file 550 px wide x 300-600 px high.

14) As part of the EMBO Publications transparent editorial process initiative (see our Editorial at http://embomolmed.embopress.org/content/2/9/329), EMBO Molecular Medicine will publish online a Review Process File (RPF) to accompany accepted manuscripts.
In the event of acceptance, this file will be published in conjunction with your paper and will include the anonymous referee reports, your point-by-point response and all pertinent correspondence relating to the manuscript. Let us know whether you agree with the publication of the RPF and as here, if you want to remove or not any figures from it prior to publication.
Please note that the Authors checklist will be published at the end of the RPF.

EMBO Molecular Medicine has a "scooping protection" policy, whereby similar findings that are published by others during review or revision are not a criterion for rejection. Should you decide to submit a revised version, I do ask that you get in touch after three months if you have not completed it, to update us on the status.

I look forward to seeing a revised form of your manuscript as soon as possible. Please use this link to login to the manuscript system and submit your revision: Link Not Available

Yours sincerely,

Lise Roth

Lise Roth, Ph.D
Editor
EMBO Molecular Medicine

***** Reviewer's comments *****

Referee #1 (Comments on Novelty/Model System for Author):

Overall the manuscript is well written, structured and provides a series of novel findings that are relevant and of great interest to the wider academic community in the cancer/DNA damage field. I have added a series of minor observations that could further improve the content and scope of the work.

Referee #1 (Remarks for Author):

The present work focuses on the transcription deregulation programs of two major SCLC subtypes i.e. SCLC-A and SCLC-N. The authors provide evidence that ASCL1 and NEUROD1 transcriptional factors are highly expressed in pulmonary neuroendocrine cells that in turn is responsible the transcription activation of several responsive genes. Further work revealed that the vicinity of ASCL1 and NEUROD1 responsive gene promoters acquire and open chromatin configuration that allows the genotoxic attack by DNA binders. In an attempt to exploit the transcription dependency of SCLC-A and SCLC-N cells and develop a therapeutic strategy, the authors used a marine derived alkaloid i.e. lurbinectedin that preferentially binds to CGG rich regfions allowing to bind to CpG rich regions located downstream of the TSS of activated genes; the patter triggers the arrest and degradation of RNAPII abrogating the transcription induction of BCL2, TTF1, INSM1 and MYC leading to SCLC cell death.

Overall the manuscript is well written, structured and provides a series of novel findings that are relevant and of great interest to the wider academic community in the cancer/DNA damage field. The following minor observations could further improve the content and scope of the work:

1. On Figure 1b and 1c, it may be useful that the authors further clarify how they determine whether genes are being transcribed (or not; cutoff values).
2. The authors state that genes may be ASCL1 dependent (Figure 1b) or NEUROD1 dependent (Figure 1c). Dependencies are not really shown in this figure and the possibility of a larger protein complex cannot be excluded. Most likely the authors mean that an X number of transcribed genes are also bound by ASCL1 or NEUROD1.
3. The authors show that SCLC cells overexpressing ASCL1 and NEUROD1 are sensitive to lurbinectedin. Do other types of tumor cell lines (preferentially those that are not poisoned for transcription induction) are also sensitive to lurbinectedin; Are the findings dependent on the transcription activators ASCL1 and NEUROD1 or the general phenomenon of transcription activation (which is rather common in several tumor cell lines)?
4. How the authors explain the binding preference of lurbinectedin for CpG motifs located at promoters of activated genes?

Referee #2 (Remarks for Author):

Peer review of the manuscript with the number EMM-2021-14841 by Costanzo F et. al. at EMBO Mol Med and with the tittle "Promoters of ASCL1- and NEUROD1-dependent genes are specific targets of lurbinectedin in SCLC cells"

1. General description of the manuscript

Costanzo F et. al. suggest lurbinectedin (a marine derived alkaloid that harbors a high specificity towards CGG rich triplets and binds CpG rich regions that are located downstream of the transcription start sites, TSS) as a novel and highly specific therapeutic drug against small cell lung cancer (SCLC), which is a neuroendocrine type of lung cancer that is characterized by an extremely aggressive course due to high cellular proliferation and early metastatic spread. Currently, first line of therapy against SCLC includes a combination of platinum based treatment, Cisplatin or Carboplatin with Etoposide or Irinotecan. While SCLC is initially chemo- and radiosensitive, this initial response is usually followed by development of resistance. Consequently, patient prognosis is poor with a median survival of 6 to 12 months.

The authors refer in the manuscript to data generated by different techniques implementing next-generation sequencing (NGS), such as RNA-seq, ChIP-seq, ATAC-seq, Chem-seq and BrdUTP ChIP-seq, analyzing biological material generated in various experiments implementing different cell lines, including control, SCLC and non-small cell lung cancer (NSCLC) cell lines.

Costanzo F et al. propose a mechanism of action of lurbinectedin that could be summarized in the following steps:
- Lurbinectedin binds to CpG rich regions located downstream of the TSS of genes with open chromatin structure that are also transcribed.
- This lurbinectedin binding induces the arrest of elongating RNAvpolymerase II (RNAPII) and its subsequent degradation.
- The expression levels of specific genes is reduced, including BCL2, NKX2-1, INSM1, MYC family members, which are involved in tumorigenesis and in neuroendocrine features of SCLC.
- Lurbinectedin ultimately triggers SCLC cell death.

The mechanism proposed by Costanzo F et al. is conceptually interesting. The authors implemented state-of-the-art techniques to generate data supporting their model. The work might be a significant scientific contribution to the field of lung cancer with a great potential for translating their findings into clinical applications. EMBO Mol Med might be an adequate platform to disseminate this work and make it accessible to the broad scientific and clinical community targeted by EMBO Mol Med. However, bevor I suggest this work for publication, the authors need to address the following concerns in order that the manuscript reach the standards of recent publications at EMBO Mol Med.

2. Major Concerns

2.1 Data availability

The model presented by the authors in the manuscript mainly relies on interpretation of data that were generated by NGS-techniques. Even though nowadays the guidelines of recognized scientific journals, including EMBO Mol Med, clearly state that the access to NGS data should be granted by depositing the raw data in specific data depositories, this was not the case for the current version of the manuscript presented by Costanzo et al. Therefore, I was not able to determine the quality of the NGS data generated by the authors, even worse, I was not able to reproduce the NGS data analysis to confirm that the data were analyzed appropriately. Due to the structure of the manuscript, the interpretation of the results and the conclusion made by the authors, the access to the raw NGS data is essential to be able to perform a complete and thought-out peer review.

Constructive Suggestions: The author should deposit the raw data (FASTAQ files) of each the NGS experiments (RNA-seq, ChIP-seq, ATAC-seq, Chem-seq and BrdUTP ChIP-seq) in a data depository, for example NCBI's Gene Expression Omnibus, and provide accession numbers, with which the Reviewers could access the data during the peer review process to determine the quality of the data and reproduce the data analysis. Most of the NGS data depositories offer the option to give access to the Reviewers only, and upon publication to release the access to the public.

2.2 Description and interpretation of the results

The description of the results is very limited and not accurate. It is not possible to read the manuscript without changing to the Figure Legends or the Material and Methods sections. Even by searching in these two sections of the manuscript, aspects that are relevant for the interpretation of the results are not presented. Moreover, the authors tend to over-interpret their results and use specific terms inappropriately, as I will elaborate in my suggestions below.

Constructive Suggestions: The authors should include during the description of the results information that supports their interpretation of the results. It should be possible to read and understand the Results section without constantly checking the Figure Legends, the Material and Methods or the Supplemental Information.

2.2.1 For example, if the authors refer to elongating RNA polymerase II (RNAPII), they should mention the specificity of the antibody used at the first time that they raise the claim. The conclusion made by the authors in Page 5, paragraph 1, related to the presence of RNAPII being deterministic of active transcription, depends on its phosphorylated status. Which antibody was used?

2.2.2 Similarly, the authors refer to "basal conditions" on page 4 during the description of the Figure 1e, but explain that the cells were treated with lurbinectedin for 4 hours on page 6 after the description of the Supplemental Figure 1d.

2.2.3 The authors should consistently include during the description of the results statistical information (P and n values) that demonstrate the statistical significance of their claims. Moreover, the authors should provide as Supplementary Material an Excel file containing a statistical summary, including all statistical relevant information from each one of the plots presented in each Figure panel, such as n values, P values, Test implemented, values used for the plots, numbers of experiments, etc. The information could be organized in the Excel file in different data sheets according to the Figure panels, in order that the reader can easily navigate through the data.

2.2.4 The description of the results obtained by different NGS techniques should be done according to the implemented technique. For example, enrichment of ASCL1 or NEUROD1 on specific genome loci detected after ChIP-seq is not a proof that specific "genes were activated by either ASCL1 (8124) or NEUROD1 (7320) (Table 1)", as written by the authors in the 2nd paragraph on page 3. Further experiments, implementing gain-of-function, loss-of-function, single gene analysis, are required to be able to make this statement written by the authors.

2.2.5 Following similar line of ideas, the authors claim "...Moreover, analyses of ChIP-seq and RNA-seq data revealed that 37% (4947) of genes being transcribed (among a total of 13340), were ASCL1 dependent (Figure 1b); similarly, 32% (4278) of transcribed genes were NEUROD1 dependent (Figure 1c)...". To be able to claim "ASCL1 dependent" or "NEUROD1 dependent", the authors need to present further experiments using ASCL1- and NEUROD1-specific loss-of-function and ideally implementing other techniques that confirm and complement their RNA-seq and ChIP-seq data, for example expression analysis of specific transcripts by qRT-PCR or ChIP analysis of specific promoters.

2.2.6 Similarly as suggested for the presented RNA-seq and ChIP-seq in the previous point, the data generated by ATAC-seq and Chem-seq should be complemented and confirmed by additional experiments analyzing selected genes, which are supposed to be bound by lurbinectedin.

2.2.7 The authors should present in the Main and Supplementary Figures Genome Browser snapshots visualizing selected genome loci of putative target genes of ASCL1, NUEROD1 and more importantly of lurbinectedin. The visualization of selected genome loci should further support the interpretation of the NGS-results generated by the authors. Moreover, the visualization of selected genome loci could serve as orientation for designing the additional experiments suggested in the points 2.2.4 to 2.2.6.

2.2.8 The use of the term "overexpression" might be misinterpreted as "ectopic expression". Since the authors do not present any experiment after overexpression (ectopic expression) of any transcript, the authors should substitute the term "overexpression" by other terms, such as "increased levels of expression", "increased transcript levels", etc. For example, the sub-title "SCLC cells overexpressing ASCL1 and NEUROD1 are sensitive to lurbinectedin" could be substituted by "High levels of ASCL1 and NUEROD1 sensitize SCLC cells to lurbinectedin", which would fit better with the presented results.

2.3 Material and Methods

The description of the Material and Methods is not sufficient for reproducing the experiments presented by the authors. The authors should avoid referring to other publications. The authors should provide sufficient technical details to be able to reproduce the "bench-side part" and the NGS-data analysis of the experiments presented in the manuscript. In case that the space is limited by the guidelines of EMBO Mol Med, the authors should provide these details in the Supplemental Information.

2.4 In the Figures 4c and 4d

The authors write that these are IPs (immune precipitations). However, in the text is written that these experiments are affinity purifications (Figure 4c) and pull-down experiments (Figure 4d), respectively. This should be corrected in the Figures and the corresponding legend. The conditions for both experiments should be described in the corresponding sub-sections of the Material and Methods. Moreover, it will be relevant for the interpretation of these experiments to present the WB also for total RNAPII, in addition to the results presented for RNAPII Ser2P.

3. Minor Concerns

3.1 Addition of line numbers to the manuscript will facilitate the peer review process.

3.2 The authors should uniformly use through the manuscript the official symbols for genes, transcripts and proteins. For example, NKX2-1 instead of TTF1 . In case that the authors prefer to use alias (as TTF1), this should be explained by the first time used in the text. Further, the authors should implement the nomenclature for nucleic acids and proteins.

3.3 In the Figures 1a and 4a, which Tubulin was used as loading control for the Western Blot analysis of protein extracts? Depending on the Tubulin detected, the official protein symbol should be used in the corresponding Figures and in the different sections of the text of the manuscript.

Why was used in the Supplementary Figure 2 a different loading controls for WB analysis (tubulin vs. vinculin)? Did the siRNA transfection affect the levels of Tubulin?

3.4 Tables shown on the right side of Figures 1b and 1c are not providing more information. No further interpretation on the "ChIPseq only" or "RNAseq only" regarding whether other TFs might be regulating those datasets. Better to perform GO or pathways enrichment from the genes located in the intersection.

3.5 In Figure 1d, if these binding motifs were identified in the ChIP-seq experiment, what is their statistical value? For instance, P values, % of genes (from the total, and those from the intersection between ChIPseq and RNAseq experiments), etc.

3.6 In Figure 1e, the intersection between ASCL1 and NEUROD1 targets (4947 and 4278) is missing and would be more relevant.

Moreover, for Figure 1e, in the intersection, to perform GO or pathways enrichment from the genes located in the intersection.

3.7 In Figure 2, were all genes included for the peak distribution analysis? What is the n for Figure 2a and for 2b. This analysis might look better at +/- 2 kb.

3.8 A genome distribution for binding sites for ASCL1 and NEUROD1 should be included in Figures 2a and 2b.

3.9 In Supplementary Figure 1a, is it 15 or 16% the regions bound by Bio-lurbinectedin being around promoters?

3.10 Where is the experimental evidence supporting the following statement written by the authors: "Of note that sequence analysis revealed that Lurbinectedin was bound within the CG rich regions (CpG islands) of the ASCL1 and NEUROD1 dependent genes."

3.11 In the Figures 3b to 3e, the description of the Y-axis is missing.

3.12 In the Figure 4a top, the authors should present the WB results for both, total RNAPII and RNAPII Ser2P. These results will be relevant for the interpretation of the experiment.

3.13 In the Figure 4b bottom, the authors should present P values (see the suggested statistical summary as an Excel file in the Supplementary Materials under point 2.2.3)

3.14 How was the time point of 4h treatment with Lurbinectedin selected?

3.15 Any prediction about the ubiquinase that might be targeting RNAPII Ser2P. As an extension of Figure 4d and to support their conclusions.

3.16 What is the P value in Figure 4e?

3.17 What is the overlap between genes downregulated by Lurbinectedin and in siNEUROD1 and siASCL1 cells?

3.18 The authors declare no competing interests. However, the work was financed and performed with the participation of at least one private company. This point might need further clarification.

4. Clear recommendation

The work presented by Costanzo F et al. will be clearly a significant contribution to the scientific and clinical community working on lung cancer, the leading cause of cancer-related deaths worldwide, whereas SCLC is the most aggressive type of lung cancer. HOWEVER, before recommending the manuscript for publication at EMBO Mol Med, I would like to suggest addressing all concerns that were listed under points 2 and 3. I hope that my suggestions help the authors to improve their manuscript, thereby reaching the standards of manuscripts recently published at EMBO Mol Med AND increasing the impact of the manuscript to the scientific community. I will be glad to peer review the revised version of the manuscript.

Referee #3 (Comments on Novelty/Model System for Author):

A model organism is not used in this paper.

Referee #3 (Remarks for Author):

The authors present data on the transcriptional changes associated with small cel lung cancer cell exposure to lurbinectedin. They argue that the effect has specificity for genes regulated by two key transcription master regulators of SCLC, ASCL1 and NEUROD1. I have several concerns with the manuscript.

Major concerns

The report does not provide novel insight into the mechanisms of action of lurbinectedin. Lurbinectedin is an alkylating agent, binding to and modifying DNA across the genome, and is already known to globally affect transcription. As shown very convincingly in this paper, it alters a huge number of genes, in the very large majority of cases resulting in downregulation of gene expression. The authors demonstrate inhibition of 8000-9000 genes. It is difficult therefore to argue for "specificity" of the response.

The association with specific inhibition of ASCL1 and NEUROD1 and their dependent genes is really entirely unconvincing. The drug affects a huge number of genes, including target genes of ASCL1/NEUROD1 and genes that have nothing to do with

ASCL1/NEURDO1, in approximately equal ratios (generally, between 25 and 70% of genes depending on context). The transcriptional program of SCLC lines that express ASCL1 and/or NEUROD1 is indeed highly dependent on these master transcriptional regulators, and if lurbinectedin is suppressing transcription globally (as is the case) then it is not at all surprising that it is also suppressing target genes of ASCL1 and NEUROD1. There is no evident specificity in the target genes demonstrated in this paper. ASCL1 and NEUROD1 dependent genes tend to be in open chromatin in cells expressing these factors; the genes are therefore more prone to disruption of alkylating agents such as lurbinectedin. It might be of interest, for example, to compare the effects of lurbinectedin on gene expression to the effects of other alkylating agents to see specific effects - my expectation is that all alkylators will suppress transcription of many many genes in the cell, including many many ASCL1/NEUROD1 target genes in SCLC.

Figure 6, where either ASCL1 or NEUROD1 are specifically targeted by siRNA, STRONGLY argues against a specific effect of lurbinectedin on ASCL1/NEUROD1 dependent genes.

The "roadblock" suggested by Figure 3 is not convincing, and does not demonstrate specificity for genes transcriptionally dependent on ASCL1 or NEUROD1. The concluding statement at the bottom of page 6 that this is demonstrates these as a "specific target for lurbinectedin" does not appear to be supported.

The observation that lurbinectedin results in ubiquitin/proteasome dependent degradation of RNAPII is quite interesting and has novelty, but (a) is not further pursued mechanistically, (b) argues AGAINST specificity for ASCL1/NEUROD1 transcriptional selectivity, and (c) argues instead for a global effect on transcription, which in fact seems consistent with all of the data presented.

The authors use ChIP-Seq to characterize "genes being transcribed" but ChIP-Seq doesn't actually measure this - it measures whether the gene locus is accessible. The assumption that this corresponds to level of transcription is not always true. The same mistake is made with regard to the presence of RNAPII - its presence suggests but does not demonstrate that the gene in question is being actively transcribed.

Although the specificity of lurbinectedin is not demonstrated here, there are interesting data, namely the large classes of genes transcription of which is dependent on ASCL1 or NEUROD1 in cell lines such as DMS-53 which expresses both. Are the genes bound by ASCL1 or by NEUROD1 in DMS-53 similarly reflected in ASCL1-only or NEUROD1-only lines? To what extent? Does this correspond with expression data by RNA-Seq?

Minor concerns

Figure 6b does not present the rankings of the pathways called out by functional annotation, or how many of the 6833 dysregulated genes are implicated in each pathway. A better figure is needed.

The authors frequently anthropomorphize the drug, and the cancer cell, suggesting they are acting in purposeful manners. For example for the drug: "This leverage is used by the marine agent lurbinectedin..."; "...this is effectively exploited by lurbinectedin..."; "...lurbinectedin efficiently takes advantage of the tumorigenic properties of SCLC..."

***** Reviewer's comments *****

Referee #1

Overall the manuscript is well written, structured and provides a series of novel findings that are relevant and of great interest to the wider academic community in the cancer/DNA damage field. I have added a series of minor observations that could further improve the content and scope of the work.

1. On Figure 1b and 1c, it may be useful that the authors further clarify how they determine whether genes are being transcribed (or not; cut-off values).

We consider a gene being transcribed, in our RNA-seq data if, at least, it has a coverage equal to or higher than 1 and a TPM (transcript per million reads) cut off value of 4 (to be confident in transcript detection). We use the definition of coverage as the number of reads per base of the gene. Coverage equal to or higher than 1 has been chosen in order to be confident that all mRNAs have been completely transcribed.

This is now added **in the legend of the new fig1 and in M & M.**

2. The authors state that genes may be ASCL1 dependent (Figure 1b) or NEUROD1 dependent (Figure 1c). Dependencies are not really shown in this figure and the possibility of a larger protein complex cannot be excluded. Most likely the authors mean that an X number of transcribed genes are also bound by ASCL1 or NEUROD1.

We now write “ASCL1 and NEUROD1 targeted genes”, according to our ChIP-seq data. By overlapping these data, we were able to evaluate the great number of genes that are targeted by either ASCL1 or NEUROD1 given the similarities between their E-boxes cognate sequence **(new Figure 1B-C, lower panel and legend)**

3. The authors show that SCLC cells overexpressing ASCL1 and NEUROD1 are sensitive to lurbinectedin. Do other types of tumour cell lines (preferentially those that are not poisoned for transcription induction) are also sensitive to lurbinectedin; Are the findings dependent on the transcription activators ASCL1 and NEUROD1 or the general phenomenon of transcription activation (which is rather common in several tumour cell lines)?

Antiproliferative activity of lurbinectedin in tumor cell lines such as human lung (A549), Ewing sarcoma (A673), colon (HCT-116, HT-29), breast (MCF7, MDA-MB-231), cervix (HeLa), and pancreas (PSN-1) cells was also observed (Santamaria-Nunez, Mol. Canc. Ther.2016).

The binding of activators initiates the formation of the transcription initiation complex which include RNAPII the general transcription factors and the Mediator to start RNA synthesis of their responsive genes. In SCLC-A or -N, abnormal increased levels of ASCL1 or NEUROD1 activators, result (as we demonstrated), in a “**continuous transcription activation**” of their responsive genes; in such case, chromatin should be almost continuously kept open and consequently being accessible for drug binding.

The mechanism of transcriptional activation in cancer cells is not always known. Here, this excess of transcriptional activity is mainly due to ASCL1 and NEUROD1 DNA binding factors. Among the (7963) genes targeted by lurbinectedin (Chem-seq), more than 50% were already targeted by either ASCL1 (ChIP-seq, 4037, 56%) or NEUROD1 (4786, 66 %) (Table 1). 2194 genes were both targeted by Lurbinectedin and downregulated (Table 2: 1672 (76%) of them were also bound by either ASCL1 or NEUROD1 (Table 3). This underlines a certain degree of specificity of lurbinectedin towards genes being under a transcriptional process directed by the ASCL1 and NEUROD1 activators.

This comment is now included in the **discussion, page 17**.

4. How the authors explain the binding preference of lurbinectedin for CpG motifs located at promoters of activated genes?

Lurbinectedin and structural analogues bind covalently trough the hydroxy-group to the exocyclic 2 amino group of a central guanine in selected triplet sequences of DNA and also establish an additional noncovalent interaction with the complementary opposite DNA strand (*E. Marco Mol. Pharma. 2005; JFM Leal, British journal of Pharmacology 2010; Feuerhahn, Chemis Biol, 2011; Santa-Maria Nunez, Mol. Canc. Ther, 2016*)**. This is now included page 8.**

Analyzing lurbinectedin binding in DMS53 cells, shows: (i) 18% of the drug bind to promoter (**Figure EV2A**); (ii) the preferential binding to CGG/GCC rich triplets (found mainly in CpG islands) located at promoter-TSS (**Figure EV2B);** (iii) co-localization of lurbinectedin binding sites and the presence of CGG rich sequences as exemplified in several genes (**new Figure 3A-D and Appendix figure S1**).

Referee #2

The mechanism proposed by Costanzo F et al. is conceptually interesting. The authors implemented state-of-the-art techniques to generate data supporting their model. The work might be a significant scientific contribution to the field of lung cancer with a great potential for translating their findings into clinical applications. EMBO Mol Med might be an adequate platform to disseminate this work and make it accessible to the broad scientific and clinical community targeted by EMBO Mol Med. However, bevor I suggest this work for publication, the authors need to address the following concerns in order that the manuscript reach the standards of recent publications at EMBO Mol Med.

2. Major Concerns

We apologize about the lack of data availability and technical clarity. We below answer point by point to the referee concerns

2.1 Data availability. The model presented by the authors in the manuscript mainly relies on interpretation of data that were generated by NGS-techniques. Even though nowadays the guidelines of recognized scientific journals, including EMBO Mol Med, clearly state that the access to NGS data should be granted by depositing the raw data in specific data depositories, this was not the case for the current version of the manuscript presented by Costanzo et al. Therefore, I was not able to determine the quality of the NGS data generated by the authors, even worse, I was not able to reproduce the NGS data analysis to confirm that the data were analyzed appropriately. Due to the structure of the manuscript, the interpretation of the results and the conclusion made by the authors, the access to the raw NGS data is essential to be able to perform a complete and thought-out peer review.

Constructive Suggestions: The author should deposit the raw data (FASTAQ files) of each the NGS experiments (RNA-seq, ChIP-seq, ATAC-seq, Chem-seq and BrdUTP ChIP-seq) in a data depository, for example NCBI's Gene Expression Omnibus, and provide accession numbers, with which the Reviewers could access the data during the peer review process to determine the quality of the data and reproduce the data analysis. Most of the NGS data depositories offer the option to give access to the Reviewers only, and upon publication to release the access to the public.

Sorry for that as it was not clear to us whether this was a process to be done before peer review. We have now uploaded the FASTQ files corresponding to ChIP-seq, Chem-seq and RNA-seq data shown in the manuscript**, "Data Availability: on Gene Expression Omnibus database (GSE179074**).

Secret password knybukcsnxsdvyp

2.2 The description of the results is very limited and not accurate. It is not possible to read the manuscript without changing to the Figure Legends or the Material and Methods sections. Even by searching in these two sections of the manuscript, aspects that are relevant for the interpretation of the results are not presented. Moreover, the authors tend to over-interpret their results and use specific terms inappropriately, as I will elaborate in my suggestions below.

Constructive Suggestions: The authors should include during the description of the results information that supports their interpretation of the results. It should be possible to read and understand the Results section without constantly checking the Figure Legends, the Material and Methods or the Supplemental Information.

We agree but adding all the information is not so easy. Accordingly, we improved the description of the experiments all along the results section.

2.2.1 For example, if the authors refer to elongating RNA polymerase II (RNAPII), they should mention the specificity of the antibody used at the first time that they raise the claim. The conclusion made by the

authors in Page 5, paragraph 1, related to the presence of RNAPII being deterministic of active transcription, depends on its phosphorylated status. Which antibody was used?

We have used an antibody recognizing the phosphorylated Ser2 of the CTD hepta-repeat YSPTSPS of the largest RNAPII subunit. Indeed, Ser2 phosphorylation is a hallmark of elongating RNAPII. In our case, Chip-seq allow us to identify genes being under a transcription process.

This is now mentioned in M &M the text, page 10.

2.2.2 Similarly, the authors refer to "basal conditions" on page 4 during the description of the Figure 1e, but explain that the cells were treated with lurbinectedin for 4 hours on page 6 after the description of the Supplemental Figure 1d.

We now refer **untreated samples** as "normal conditions, t=0" and **treated samples** when exposed to lurbinectedin treatment (t=4h), all over the text.

2.2.3 The authors should consistently include during the description of the results statistical information (P and n values) that demonstrate the statistical significance of their claims. Moreover, the authors should provide as Supplementary Material an Excel file containing a statistical summary, including all statistical relevant information from each one of the plots presented in each Figure panel, such as n values, P values, Test implemented, values used for the plots, numbers of experiments, etc. The information could be organized in the Excel file in different data sheets according to the Figure panels, in order that the reader can easily navigate through the data.

*An Excel file named "**Statistical Summary**",* containing all the statistics and implemented tests for each Figure panel and respective plots **is now provided with the manuscript to the editor**.

2.2.4 The description of the results obtained by different NGS techniques should be done according to the implemented technique. For example, enrichment of ASCL1 or NEUROD1 on specific genome loci detected after ChIP-seq is not a proof that specific "genes were activated by either ASCL1 as written by the authors in the 2nd paragraph on page 3. Further experiments, implementing gain-of-function, loss-of-function, single gene analysis, are required to be able to make this statement written by the authors.

Accordingly, we modify "…genes were activated by either…" to "genes were targeted by either ASCL1(8131) or NEUROD1 (7319) (**Table 1**)". See also below "**Answers to the 2.2.4-2.2.7 concerns**". Silencing experiments also show that among the 1698 genes downregulated upon silencing ASCL1, 930 genes were found downregulated by Lurbinectedin in DMS53 cells; similarly, among the 464 genes downregulated upon silencing NEUROD1, 217 genes were also found downregulated by Lurbinectedin in DMS53 cells **(Appendix S2).**

2.2.5 Following similar line of ideas, the authors claim "...Moreover, analyses of ChIP-seq and RNA-seq data revealed that 37% (4947) of genes being transcribed (among a total of 13340), were ASCL1 dependent (Figure 1b); similarly, 32% (4278) of transcribed genes were NEUROD1 dependent (Figure 1c)...". To be able to claim "ASCL1 dependent" or "NEUROD1 dependent", the authors need to present further experiments using ASCL1- and NEUROD1-specific loss-of-function and ideally implementing other techniques that confirm and complement their RNA-seq and ChIP-seq data, *for example expression analysis of specific transcripts by qRT-PCR or ChIP analysis of specific promoters.*

As mentioned above, we now write: "… were ASCL1 and/or NEUROD1 targeted…". Moreover, among the 2194 downregulated genes that were targeted by Lur, 1672 (76%) were bound by ASCL1 and NEUROD1 (**Table 3**), which underlined a certain degree of specificity of Lurbinectedin towards genes that are targeted by either ASCL1 or NEUROD1 transcription factors.

See also below: "**Answers to the 2.2.4-2.2.7 concerns**"

2.2.6 Similarly as suggested for the presented RNA-seq and ChIP-seq in the previous point, the data generated by ATAC-seq and Chem-seq should be complemented and confirmed by additional experiments analysing selected genes, which are supposed to be bound by lurbinectedin.

As now indicated in the **new figures 3A-D,** we provide a ChIP-seq genome track of a selection of genes *ASCL1, BCL2, INSM1* and *MYB*. Similar patterns were obtained from another experiment **(Appendix figure S1).**

Answers to the 2.2.4-2.2.7 concerns

2.2.7 The authors should present in the Main and Supplementary Figures Genome Browser snapshots visualizing selected genome loci of putative target genes of ASCL1, NUEROD1 and more importantly of lurbinectedin. The visualization of selected genome loci should further support the interpretation of the NGS-results generated by the authors. Moreover, the visualization of selected genome loci could serve as orientation for designing the additional experiments suggested in the points 2.2.4 to 2.2.6.

We now will Answer to the 2.2.4-2.2.7 concerns:

To be able to assert that genes are "ASCL1 dependent" or "NEUROD1 dependent", we now implemented the manuscript with additional experiments/data:

1). Genome browser visualization showed that in untreated conditions, ASCL1 or NEUROD1 bound to a number of genes as exemplified for *ASCL1, BCL2, INSM1* and *MYB*, being read by elongating RNAPII and in open chromatin state according to the presence of H3K27ac and ATAC mark (**Figure 3A-D and Appendix S1A-D).** At 4 h post lurbinectedin treatment, these genes were found to be targeted by lurbinectedin at their CGG rich regions located downstream from their TSS as visualized at the bottom of each panel;

2). Considering **Figure 6B:** *ASCL1 gene is expressed in NCI-H510A* (in which ASCL1 is present and NEUROD1 missing) *but not* in NCI-H82 (in which only NEUROD1 is present). Similarly, *NEUROD1* is expressed in NCI-H82 but not in NCI-H510A. BCL2 and INSM1 are not or weakly expressed in NCI-H82 due to the lack of NEUROD1;

3). We also did q-PCR on 8 genes treated either by cisplatin or lurbinectedin and observed how powerful is lurbinectedin tin downregulating a large number of genes (**new Figure 5G**).

4). Moreover, as examples, RT-PCR identify ASCL1 and BCL2 in ChIp-seq-ASCL1, -NEUROD1, Chem-Lur fractions (**new Figure EV3A and EV3B**) that were found being down regulated upon Lurbinectedin treatment.

2.2.8 The use of the term "overexpression" might be misinterpreted as "ectopic expression". Since the authors do not present any experiment after overexpression (ectopic expression) of any transcript, the authors should substitute the term "overexpression" by other terms, such as "increased levels of expression", "increased transcript levels", etc. For example, the sub-title "SCLC cells overexpressing ASCL1 and NEUROD1 are sensitive to lurbinectedin" could be substituted by "High levels of ASCL1 and NUEROD1 sensitize SCLC cells to lurbinectedin", which would fit better with the presented results.

We now use all over the manuscript, the term “increased levels of expression” which is more accurate.

2.3 Material and Methods

The description of the Material and Methods is not sufficient for reproducing the experiments presented by the authors. The authors should avoid referring to other publications. The authors should provide sufficient technical details to be able to reproduce the "bench-side part" and the NGS-data analysis of the experiments presented in the manuscript. In case that the space is limited by the guidelines of EMBO Mol. Med., the authors should provide these details in the Supplemental Information.

The description of NGS methodologies has now being implemented in Material & Methods with a detailed explanation of software utilized to generate the data shown in this manuscript.

2.4 In the Figures 4c and 4d

The authors write that these are IPs (immune precipitations). However, in the text is written that these experiments are affinity purifications (Figure 4c) and pull-down experiments (Figure 4d), respectively. This should be corrected in the Figures and the corresponding legend. The conditions for both experiments should be described in the corresponding sub-sections of the Material and Methods.

This is now modified in the **legend of figure 4** and description of the experiments added in **M & M.**

Moreover, it will be relevant for the interpretation of these experiments to present the WB also for total RNAPII, in addition to the results presented for RNAPII Ser2P.

As now shown on the **new figures 4D and 4E**, we present a WB for total RNAPII, showing the absence of unphosphorylated RNAPII following lurbinectedin treatment.

3. Minor Concerns

3.1 Addition of line numbers to the manuscript will facilitate the peer review process.

This could be done but it was usually not requested by the authors information.

3.2 The authors should uniformly use through the manuscript the official symbols for genes, transcripts and proteins. For example, NKX2-1 instead of TTF1. In case that the authors prefer to use alias (as TTF1), this should be explained by the first time used in the text. Further, the authors should implement the nomenclature for nucleic acids and proteins.

This has now been done through the text

3.3 In the Figures 1a and 4a, which Tubulin was used as loading control for the Western Blot analysis of protein extracts? Depending on the Tubulin detected, the official protein symbol should be used in the corresponding Figures and in the different sections of the text of the manuscript.

We have utilized α-tubulin; it is now modified figures 1A, 4A and EV1B-C.

Why was used in the Supplementary Figure 2 a different loading controls for WB analysis (tubulin vs. vinculin)?

The molecular weight for NEUROD1 is 45-50kda and Tubulin is 55kda. To avoid overlapping signal in the WB, we used Vinculin as it runs at 120kDa. We now present another experiment performed in **parallel (Figure EV1B-C**)

Did the siRNA transfection affect the levels of Tubulin?

siRNA transfection did not affect the level of Tubulin gene expression in our experimental conditions, as demonstrated by RNA-seq as well as western blot in **Figure EV3C**.

3.4 Tables shown on the right side of Figures 1b and 1c are not providing more information. No further interpretation on the "ChIPseq only" or "RNAseq only" regarding whether other TFs might be regulating those datasets.

Information on the tables of figure 1B and 1C are now indicated in the legend.

We were focusing on ASCL1 and NEUROD1 activators that target E-boxes present in a certain number of genes identified in our RNA-seq dataset. The overlap between ASCL1 ChIP seq or NEUROD1 ChIPseq with RNA seq is used to funnel down the genes targeted by these transcription factors. We hence do not focus on the non-overlapping dataset as deviating from the purpose of the manuscript.

Better to perform GO or pathways enrichment from the genes located in the intersection.

GO and pathways analysis on the genes located in the intersection between ChIP-seq and RNA-seq data sets in Figure 1, are now presented **as a new Figure EV4A and EV4B** and show that most of the ASCL1 and/or NEUROD1 targeted genes are associated with Transcription; KEGG ontology show that those genes are actively transcribed genes in Cancer cells.

3.5 In Figure 1d, if these binding motifs were identified in the ChIP-seq experiment, what is their statistical value? For instance, P values, % of genes (from the total, and those from the intersection between ChIP-seq and RNAseq experiments), etc.

The p-values of the ASCL1 and NEUROD1 motif in our ChIP-seq experiment are P=1e-6800 and P=1e-60, respectively.

Intersection between ASCL1/NEUROD1 and RNA-seq dataset, revealed how 4864 of transcribed genes in ASCL1 ChIP-seq (91% of the 5357) were containing ASCL1 motif. Similarly, 3573 of transcribed genes in NEUROD1 ChIP-seq (79% of the 4551) were containing NEUROD1 motif (**Figure 1B-C**). Interestingly, we found that 3470 genes (almost 26% of the 13254 transcribed genes) were actively transcribed and targeted by ASCL1 and NEUROD1."

These comments are added to the text page 7

3.6 In Figure 1e, the intersection between ASCL1 and NEUROD1 targets (4947 and 4278) is missing and would be more relevant.

The significant overlapping between ASCL1 and NEUROD1 targeted genes is now presented (**New figures 1B and 1C lower panel**). A large number of genes might be targeted by either ASCL1 and/or NEUROD1 given the similarities between the E-box sequences.

Moreover, for Figure 1e, in the intersection, to perform GO or pathways enrichment from the genes located in the intersection.

GO and pathways analysis on the genes located in the intersection between ChIP-seq and RNA-seq data sets in Figure 1, are now presented as **Figure EV4A and EV4B** and pointed out that a large part of the genes was involved in ongoing transcription and cancer development.

3.7 In Figure 2, were all genes included for the peak distribution analysis?
For the peak profiles, the ChIp-seq signal distribution has been produced by taking in consideration the TSS coordinates corresponding to all genes from hg19.
What is the n for Figure 2a and for 2b.
For the profiles shown in figure 2a and 2b the n corresponding to the ChIP-seq shown is equal to 1. However, these profiles (comprising all the genes in hg 19) have been funnelled down by overlapping with our RNAseq gene lists derived from 5 different cell lines (DMS-53, DMS-53 siASCL1, DMS-53 siNEUROD1, NCI-H510A, NCI-H82) and 2 different conditions each (untreated vs Lurbinectedin treated). The RNAseq and ChIPseq experiments have been performed in the same experimental conditions e.g.at the same time, at the same lurbinectedin concentration and in the same culture/calf serum conditions, to avoid potential batch effects and to augment the degree of confidence of our experimental condition and the final message of our manuscript.
This analysis might look better at +/- 2kb
The analysis at +/-2kb was done on **new figures 2A-E,2G and figure 3E-G**.

3.8 A genome distribution for binding sites for ASCL1 and NEUROD1 should be included in Figures 2a and 2b.

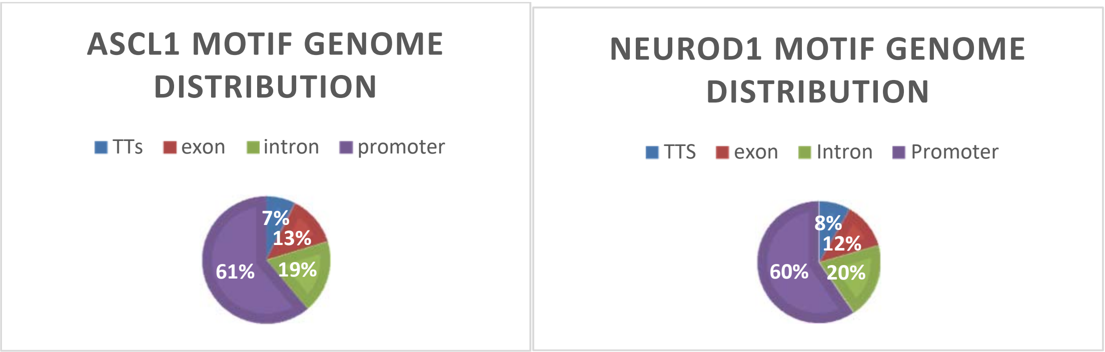

These graphs are presented **in figures EV1A**.

3.9 In Supplementary Figure 1a, is it 15 or 16% the regions bound by Bio-lurbinectedin being around promoters?
We repeated the calculation as described in material and methods and found that approximately 18% of lurbinectedin was bounds around promoters (F**igure EV2A**).

3.10 Where is the experimental evidence supporting the following statement written by the authors: "Of note that sequence analysis revealed that Lurbinectedin was bound within the CG rich regions (CpG islands) of the ASCL1 and NEUROD1 dependent genes."
To assess the binding of Lurbinectedin on CG rich regions, we searched 3-mer motifs corresponding to different DNA triplets into a -/+1 kb sequence area around lurbinectedin promoter-located peaks and random regions of similar size from hg19 genome promoters (as background reference) and their presence in 10 bp bins were counted and a score were calculated for all the regions of interest using Homer suite v4.1. The result showing CCG is now added as F**igure EV2B** whether the remaining 3-mer sequences are enclosed as a new source data file for the manuscript.

3.11 In the Figures 3b to 3e, the description of the Y-axis is missing.

The y axis on every Chip-seq profile represents the Coverage Score is now added to the figures.

3.12 In the Figure 4a top, the authors should present the WB results for both, total RNAPII and RNAPII Ser2P. These results will be relevant for the interpretation of the experiment.
This is now included in the **new figure 4A, 4D and 4E**

3.13 In the Figure 4b bottom, the authors should present P values (see the suggested statistical summary as an Excel file in the Supplementary Materials under point 2.2.3)
Figure 4C show the statistical values of the decrease of RNAPII fluorescence intensity (Figure 4B) signal after Lurbinectedin treatment. Respective p-values corresponding to the stars are now added in the figure legend.
This is now included in the Excel file in the suggested **Statistical Summary** added with the manuscript.

3.14 How was the time point of 4h treatment with Lurbinectedin selected?
In all the experiments we used; 50nM Lurbinectedin and collected the cells at 4h. For selecting this dosage and time, we have performed both a time-response and a dosage-response in SCLC-DMS53 cells. In these conditions, depending also of the culture medium and the cells themselves, we were still able to detect elongating RNAPII (IIO), the degradation of which was starting to be engaged as shown in the **new Figure EV1B and EV1C and in the legend**. Of course, this results in SCLC cell lines matches with several preliminary experiments and previous data as such presented in the figure 3 of the Santamaria Nunez 2016 paper for A549 cells and others.

3.15 Any prediction about the ubiquinase that might be targeting RNAPII Ser2P. As an extension of Figure 4d and to support their conclusions.
As previously observed, the transcription activation complex contains in addition to the DNA binding activators, mediator, the basal transcription factors and RNAPII proteins involved in the Ubiquitin degradation system such as SUG 1 part of the proteasome (Weeda, NAR, 1997, Fraser, JBC, 1997) as well as ELOF1, CSB, CSA to be associated with RNAPII ubiquitination and further proteasome degradation system (van der Weegen, Nat. Cell. Bio., 2021; Nakasawa, Cell, 2020; Anindya ; Mol. Cell, 2010). They likely might participate to RNAPII degradation. This point is documented in the **discussion page 17.**

3.16 What is the P value in Figure 4e?
The p-value of Figure 4e is =0.005476 according to Kolmogorov-Smirnov test. This is a compilation of dfata from Figure 2 to better show the positioning of Lur, RNAPII, H3K27ac towards TSS This is **now Figure 4F.**

3.17 What is the overlap between genes downregulated by Lurbinectedin and in siNEUROD1 and siASCL1 cells?
In our experimental conditions, among 1698, SiASCL1 share 930 genes in common with the list of genes downregulated by Lurbinectedin. Among 464, siNEUROD1 cell line share 217 genes in common with the list of genes downregulated by Lurbinectedin (**Appendix Figure S2**); this is now added page 12

3.18 The authors declare no competing interests. However, the work was financed and performed with the participation of at least one private company. This point might need further clarification.
Our laboratory has a longstanding collaboration (Up to 15 years) with Pharma Mar and have received partial research support from them, the rest providing mainly from the french National Research Agency, the Association pour la Recherche contre le Cancer as well as from European (ERC) grants and from Asian and Korean Research Societies. The work performed in collaboration with Pharma Mar deals with fundamental research to understand the mechanism of action of DNA binders that block transcriptional process and especially RNAPII (Feuerhahn, Chem & Bio, 2011; Santa-Maria Nunez, Mol. Canc. The., 2016). Given that, I found absolutely necessary to work with such highly advanced chemists such as those working in the Pharma Mar company that were designing drugs. MM. D., G. SN, JI. DH, J. DP and EM. GM are employees of Pharma Mar SA (Madrid, Spain). Data and materials availability: lurbinectedin is available

from Pharma Mar for noncommercial use under an MTA. All relevant data are included within this manuscript and all materials other than lurbinectedin are readily available on the link proposed in the paper and upon request.

4. Clear recommendation
The work presented by Costanzo F et al. will be clearly a significant contribution to the scientific and clinical community working on lung cancer, the leading cause of cancer-related deaths worldwide, whereas SCLC is the most aggressive type of lung cancer. HOWEVER, before recommending the manuscript for publication at EMBO Mol Med, I would like to suggest addressing all concerns that were listed under points 2 and 3. I hope that my suggestions help the authors to improve their manuscript, thereby reaching the standards of manuscripts recently published at EMBO Mol Med AND increasing the impact of the manuscript to the scientific community. I will be glad to peer review the revised version of the manuscript.
We are very thankful. I hope we have answered to the referee concerns. Please if additional information is needed, you may ask. We also apologise for taking time but my postdoc being abroad, the Covid situation to make more experiments raised did not facilitate to respond so quickly. We hope that we have answered to most of your requests. Please if we were unclear, don't hesitate to ask
Merci beaucoup.

Referee #3

Major concerns
The report does not provide novel insight into the mechanisms of action of lurbinectedin. Lurbinectedin is an alkylating agent, binding to and modifying DNA across the genome, and is already known to globally affect transcription. As shown very convincingly in this paper, it alters a huge number of genes, in the very large majority of cases resulting in downregulation of gene expression. The authors demonstrate inhibition of 8000-9000 genes. It is difficult therefore to argue for "specificity" of the response. The association with specific inhibition of ASCL1 and NEUROD1 and their dependent genes is really entirely unconvincing.
I first would like to apologise as we confused the referee especially concerning the Lurbinectedin specificity and the silencing of ASCL1 and NEUROD1. In addition, **I r**eally thank the referee for his strong concerns that obviously oblige us to be clearer and provide more explanations.
Lurbinectedin is an alkylating agent that bind to the central G of nucleotides triplet and interacts with the opposite DNA strand (through hydrogen bond and van der Waals interactions (*E. Marco Mol. Pharma. 2005; JFM Leal, British journal of Pharmacology 2010; Feuerhahn, Chemis Biol, 2011; Santa maria Nunez, Mol. Canc. Ther, 2016)*. This is now mentioned **on page 8.** Surprisingly, up to 18% of the (50nM) drug bound specifically to promoter regions 4h after treatment (**Figure 2G, new Fig. EV2A**), and more precisely to CGG motifs which are very often found at the promoter regions at a short distance from the TSS (around 250 bp) (Figure **3F**), as also exemplified by ChIP-seq genome track for *ASCL1, BCL2, INSM1* and *MYB* genes, all of them being targeted by ASCL1 and/or NEUROD1, read by elongating RNAPII and in open chromatin state according to the presence of H3K27ac (**new Figure 3A-D**), underlining a certain degree of specificity of this DNA-binder in targeting genes being actively transcribed.
We have tried to make it clearer in the text.
The drug affects a huge number of genes, including target genes of ASCL1/NEUROD1 and genes that have nothing to do with ASCL1/NEURDO1, in approximately equal ratios (generally, between 25 and 70% of genes depending on context). The transcriptional program of SCLC lines that express ASCL1 and/or NEUROD1 is indeed highly dependent on these master transcriptional regulators, and if lurbinectedin is suppressing transcription globally (as is the case) then it is not at all surprising that it is also suppressing target genes of ASCL1 and NEUROD1. There is no evident specificity in the target genes demonstrated in this paper. ASCL1 and NEUROD1 dependent genes tend to be in open chromatin in cells expressing these factors; the genes are therefore more prone to disruption of alkylating agents such as lurbinectedin.

We understand the referee concerns and apologize for having been unclear. We should point out that ASCL1 and NEUROD1 even more than other TFs, have pioneering activity, (Ireland, Micinski et al., 2020, Rudin et al., 2019) e.g. they activate a large number of genes upon binding to their E-box cognate sequence. Such binding is followed by a chromatin modification as shown by ATAC and H3K27ac signatures (**Figure 2C-D and Table 1**), thus allowing the formation of the preinitiation complex and further the reading of the gene by RNAPII. The opening of the chromatin represents an opportunity for lurbinectedin which might target the DNA at some CGG rich motifs located at the CpG islands.
Moreover, RNA-seq show that a huge number of (6785) genes were down regulated, 4h after 50nM lurbinectedin treatment of DMS53 cells (**Table 2**). As mentioned by the referee, "the transcriptional program of SCLC lines is indeed highly dependent on expressed ASCL1 and/or NEUROD1 transcriptional regulators. Among them, 2194 were targeted by 50nM lurbinectedin 4h post treatment and downregulated **(Table 3):** 76% (1672) of them are ASCL1 and/or NEUROD1 targeted demonstrating a certain degree of specificity; the others are genes either regulated by the proteins produced by the ASCL1/NEUROD1 dependent genes or housekeeping genes being likely in an open chromatin conformation.
It might be of interest, for example, to compare the effects of lurbinectedin on gene expression to the effects of other alkylating agents to see specific effects - my expectation is that all alkylators will suppress transcription of many many genes in the cell, including many many ASCL1/NEUROD1 target genes in SCLC.
According to referee suggestion, we did RNA-seq upon cisplatin treatment.
DMS53 cells were treated either by lurbinectedin (50nM, 4h) or by cisplatin (50µM, 4h) based on its IC50 in these cells according to experiments presented Figure 5D and 5E. We observed that: (i) cisplatin treatment downregulated much less genes (1609, Figure 5F) that lurbinectedin (6785, Table 2); (ii) Moreover, cisplatin seems to be less efficient than lurbinectedin in downregulating ASCL1 and NEUROD1 genes according to q-PCR experiments (**Figure 5G**); (iii) Of the 1609 downregulated genes after high dose cisplatin treatment, only 690 were targeted by ASCL1, 634 by NEUROD1 and 462 by both ASCL1 and NEUROD1. This comment is added page 13.
Figure 6, where either ASCL1 or NEUROD1 are specifically targeted by siRNA, STRONGLY argues against a specific effect of lurbinectedin on ASCL1/NEUROD1 dependent genes.
We apologise for having confused the referee. DMS53 cells were overexpressing both ASCL1 and NEUROD1 having a rather similar cognate DNA binding sequence (Figure 1D). Accordingly, silencing either ASCL1 or NEUROD1, was not sufficient to observed a drop in RNA synthesis. We only demonstrated that these proteins might regulate the same genes (lower part of Figure 1B and 1C). See above.

The "roadblock" suggested by Figure 3 is not convincing, and does not demonstrate specificity for genes transcriptionally dependent on ASCL1 or NEUROD1. The concluding statement at the bottom of page 6 that this is demonstrates these as a "specific target for lurbinectedin" does not appear to be supported.
After having demonstrated that up to 18% of Lur target the promoter of a certain number of genes that are in an open chromatin conformation (Figures 2 and 3), we observed that among the 2194 genes targeted by lurbinectedin 4h post treatment and downregulated, **76% (1672) are ASCL1 and/or NEUROD1 targeted (new Table 3)** which represent a certain degree of specificity.
We also show that a large number of them (around 60%) are targeted by RNAPII suggesting also that they are likely being transcribed. We next provide as an example the gene browser of 4 selected genes bound by ASCL1/NEUROD1, marked by ATAC and H3K27ac signatures and targeted by RNAPII; 4h post treatment, we observed that Lurbinectedin binding overlapped with the CGG rich regions in 2 repeated experiments **(Figure 3A-D and Appendix figure S1A-D).**
The observation that lurbinectedin results in ubiquitin/proteasome dependent degradation of RNAPII is quite interesting and has novelty, but (a) is not further pursued mechanistically, (b) argues AGAINST specificity for ASCL1/NEUROD1 transcriptional selectivity, and (c) argues instead for a global effect on transcription, which in fact seems consistent with all of the data presented.
We did not pursue mechanistically how RNAPII was degraded which was not the aim of our paper. However, according to the referee concern, we add in the discussion the following points.
We and others have previously shown that transcription activation complex might contain some proteins from the ubiquitin/proteasome degradation machinery, (Fraser, JBC, 1997; Weeda, NAR, 1997; Groisman, Genes and Deve. 2006; Svejstrup lab). Upon genotoxic attack CSA part of a ubiquitin ligase complex was

recruited around the RNAPII transcription (Epantchisev, Mol Cell, 2017). We here show that Lur is located upstream to TSS and that RNAPII is located in between at a certain distance downstream to Lurbinectedin (**Figure 3E-H**). Moreover, pulling down proteins by Bio-lur contain RNAPII that is ubiquitinated (Figure 4D-E). We previously have shown that lurbinectedin treatment of HeLa or AS549 cells by MG32, a proteasome inhibitor, prevent phosphorylated (elongating) RNAPII degradation (Santamaria Nunez (Mol The, 2016). RNAPII. These points are now included in the Discussion page 17.
Our data are also supported by the fact that inhibition of the proteasome with Bortezomib (through binding the catalytic site of the 26S proteasome) after Lurbinectedin treatment, led to the recovery of RNAPII signal but not of ASCL1 and NEUROD1 (see figure below).

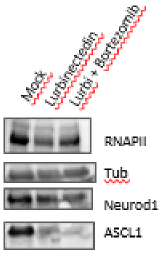

One cannot exclude that genes independent of ASCL1 and/or NEUROD1 and being under a transcriptional process (such as Housekeeping genes) light also be downregulated as also observed. Similarly, genes being dependent of the products of ASCL1 and NEUROD1 targeted genes might also be indirectly downregulated. However, it was surprising to notice the large number of ASCL1 and NEUROD1 genes downregulated by lurbinectedin.
The authors use ChIP-Seq to characterize "genes being transcribed" but ChIP-Seq doesn't actually measure this - it measures whether the gene locus is accessible. The assumption that this corresponds to level of transcription is not always true. The same mistake is made with regard to the presence of RNAPII - its presence suggests but does not demonstrate that the gene in question is being actively transcribed.
The referee is right; this remark was also provided by the other referees. We will modify and write "genes being targeted". However, Chip-seq show that genes are (i) targeted by activators ASCL1 and NEUROD1, (ii) bound by RNAPII, (iii) surrounded by H3K27ac, a hallmark of enhancer activity strengthened by ATAC signature, all of these indicate that they are likely being submitted to an active transcription process (4089 for ASCL1 and 3215 for NEUROD1, **Table 1**). Moreover, the presence of RNAPII (further involved in the ubiquitin/proteasome degradation process) and to the fact that chromatin was opened according to ATAC and H3K27ac signatures, suggest that these genes are likely being involved in a transcription process (**Figure 2A-E and 2G).** Previous work (Santamaria-Nunez, 2016) demonstrated that degradation of RNAPII occurred only if phosphorylated. **Figure 4D and 4E** show that RNAPII located near Lurbinectedin was phosphorylated, likely being elongating, a prerequisite for its further degradation when stalled in front of the lesion. We enthusiastically overinterpreted a little; we now moderated our writing.
Although the specificity of lurbinectedin is not demonstrated here, there are interesting data, namely the large classes of genes transcription of which is dependent on ASCL1 or NEUROD1 in cell lines such as DMS-53 which expresses both. Are the genes bound by ASCL1 or by NEUROD1 in DMS-53 similarly reflected in ASCL1-only or NEUROD1-only lines? To what extent? Does this correspond with expression data by RNA-Seq?
Accordingly, we defined the number of transcripts from NCI-H82 and NCI-H510A cell lines with the same parameters used for DMS-53 in figure 1 (i.e. coverage equal to or higher than 1 and a TPM cut off value of 4). We found that in DMS53, 13255 genes were transcribed, 11750 genes in NCI-H82 and 13140 genes In NCI-H510A. Of the 5357 transcribed genes bound by ASCL1 in DMS53: 4492 were commonly found to be transcribed in NCI-H82 (NEUROD1 only) cell line, whether 4938 were found to be commonly transcribed in NCI-H510A (ASCL1 only) cell line. Of the 4551 transcribed genes bound by NEUROD1 in DMS53: 3770 were commonly found to be transcribed in NCI-H82 cell line, whether 4163 were found to be commonly transcribed in NCI-H510A cell line.
This is now added page14 and shown as a figure in **Appendix Figure S3A-C)**

Minor concerns

Figure 6b does not present the rankings of the pathways called out by functional annotation, or how many of the 6833 dysregulated genes are implicated in each pathway. A better figure is needed.

We keep in account your comment and modify Figure 6A and 6B

The authors frequently anthropomorphize the drug, and the cancer cell, suggesting they are acting in purposeful manners. For example, for the drug: "This leverage is used by the marine agent lurbinectedin..."; "...this is effectively exploited by lurbinectedin..."; "...lurbinectedin efficiently takes advantage of the tumorigenic properties of SCLC..."

The referee is right; I like to see the bottle half full and maybe I anthropomorphise too much. The transcription addiction is mainly responsible of growing tumours in cancer cells. A large number of genes are being almost continuously being transcribed as also observed in the present study. In fact, most of the time, their promoters are in an open chromatin conformation status according to ATAC and H3K26ac signatures (**Figures 2C and 2D**) to allow the reading by RNAPII (**Figure 2E**). This situation is beneficial to DNA binders such as Lurbinectedin who exhibit specificity towards CGG triplets mainly found in CpG islands, Chromatin being opened. In fact, this situation “transcription addiction” mainly directed by ASCL1 and NEUROD1 is effectively exploited by lurbinectedin.

W14e will modulate our writing.

2nd Feb 2022

Dear Prof. Egly,

Thank you for submitting your revised manuscript to EMBO Molecular Medicine.
As discussed in our previous correspondence, while referee #3 is satisfied with the revision pending minor modifications, referee #2 still raises several serious concerns.
In particular, this referee regrets not having access to the raw NGS data. As per our journal policy, it is mandatory to deposit primary raw datasets generated in this study in an appropriate public database, and the accession numbers and database should be listed under 'Data Availability'.

We thus contacted you to request deposition of the data, and you kindly notified us today that the raw data were now publicly accessible.
Therefore, in a revised version of the current manuscript, we would like you to address the remaining concerns from both referees. Please be aware that this will be the last chance for you to address these points.

Additionally, please also address the following editorial issues:
- Please address the queries from our data editors in track changes mode in the main manuscript file labelled 'Data edited MS file'. Please use this file for any further modification (in track changes mode).
- Please provide up to 5 keywords
- Javier Diez Perez is currently not entered in our submission system and is also missing from the authors' contribution. Please also double-check the discrepancy between Carlos Maria Genes (in the manuscript) and Carlos Mario Gene Robles (in the submission system).
- Kindly rename the "References" section.
- Please upload the two EV tables as two separate files. Their legends should be removed from the manuscript and added to the respective file.
- Please include the Statistical Summary in the Appendix.
- Tables 1-4 should have their legends underneath the respective tables. The appendix legends should be removed from the manuscript and moved to the appendix.
- Please carefully check that all figures and figure panels are referenced in the text. (Callouts are currently missing for Fig. EV3A, B).
- Kindly provide a synopsis image as an independent tiff, jpeg or PNG file 550 px wide x 300-600 px high.
- As part of the EMBO Publications transparent editorial process initiative (see our Editorial at http://embomolmed.embopress.org/content/2/9/329), EMBO Molecular Medicine will publish online a Review Process File (RPF) to accompany accepted manuscripts.
This file will be published in conjunction with your paper and will include the anonymous referee reports, your point-by-point response and all pertinent correspondence relating to the manuscript. Let us know whether you agree with the publication of the RPF and as here, if you want to remove or not any figures from it prior to publication.
Please note that the Authors checklist will be published at the end of the RPF.

I look forward to receiving your revised manuscript.

With kind regards,

Lise Roth

Lise Roth, PhD
Editor
EMBO Molecular Medicine

***** Reviewer's comments *****

Referee #2 (Remarks for Author):

Peer review of the manuscript with the number EMM-2021-14841-V2, which is a revised version of the manuscript with the number EMM-2021-14841, by Costanzo F et. al. at EMBO Mol Med and with the tittle " Promoters of ASCL1- and NEUROD1-dependent genes are specific targets of lurbinectedin in SCLC cells"

1. General description of the manuscript

For the general description of the work presented by Costanzo F et. al., as well as for the relevance of the contribution to scientific field, I would like to refer to the text of my peer review of the previous version of the manuscript.

Regarding the revised version of the manuscript, I do recognize that the current version of the manuscript have improved significantly. The authors have tried to answer most of the concerns of the reviewers, and have included some additional experiments. However, critical aspects have not been addressed, as I will explain in the following points.

2. Major Concerns

2.1 Data availability

As I have previously written, the model presented by Costanzo F et. al. mainly relies on interpretation of data that were generated by NGS-techniques. I clearly suggested to the authors to deposit raw data (FASTAQ files) of each the NGS experiments (RNA-seq, ChIP-seq, ATAC-seq, Chem-seq and BrdUTP ChIP-seq) in a data depository, for example NCBI's Gene Expression Omnibus, and provide accession numbers, with which the Reviewers could access the data during the peer review process to determine the quality of the data and reproduce the data analysis.

First, the authors provided in the point-by-point response to the reviewers an accession number and a password that did not contain the data from all the NGS experiments that were referred to in the manuscript. The data from the Chem-seq and BrdUTP ChIP-seq were not inside of this first accession number. Upon request, the authors provided a second accession number and the password with the information for Chem-seq and BrdUTP ChIP-seq.

Second, whereas the authors write in the point-by-point response to the reviewers that they uploaded the raw data into the data depository as follows:
"We have now uploaded the FASTQ files corresponding to ChIP-seq, Chem-seq and RNA-seq data shown in the manuscript, "Data Availability: on Gene Expression Omnibus database (GSE179074)."
, the authors write on page 29 of the revised version of the manuscript:
"Sequence and processed data had been submitted to National Center for Biotechnology Gene Expression Omnibus with GEO accession number GSE179074."

Sequence is not equal to FASTAQ file.

Third, after checking both accession numbers provided by the authors, I only found processed data. I did not find the FASTAQ files.

Even though nowadays the guidelines of recognized scientific journals, including EMBO Mol Med, clearly state that the access to NGS data should be granted by depositing the raw data in specific data depositories, this was not the case for the previous version AND for the current revised version of the manuscript presented by Costanzo et al. Therefore, I was not able to determine the quality of the NGS data generated by the authors, even worse, I was not able to reproduce the NGS data analysis to confirm that the data were analyzed appropriately. Due to the structure of the manuscript, the interpretation of the results and the conclusion made by the authors, the access to the raw NGS data is essential to be able to perform a complete and thought-out peer review.

This is a critical aspect. Just to clarify to the Editors, I would like to make a comparison: It is as one peer reviewer ask for the original pictures of microscopy pictures OR the original picture of a Western Blot without any processing, and the authors do not provide it.

2.2 Specificity of the effects of Lurbinectedin on ASCL1- and NEUROD1-dependent promoters in SCLC cells
Based on the previous point 2.1 and on the results presented in the manuscript, specially the new results presented on the Figure 4A, where the authors show a Western Blot analyzing the total levels of DNA-dependent RNA Polymerase II (Pol II, it is difficult to sustain their claims related to the specificity of the effects of Lurbinectedin on ASCL1- and NEUROD1-dependent promoters in SCLC cells.

I observe on the Figure 4A that Lurbinectedin reduced the levels of total Pol II in almost all of the cell lines analyzed. These results point into the direction of a global effect of Lurbinectedin on total levels of Transcription, despite of ASCL1- and NEUROD1-dependent promoters. I think that reviewer 3 also commented on this specific point in the previous version of the manuscript.

Another point that is important to mention: the authors implemented for the analysis of total levels of Pol II a Mouse anti-RNAPII 7c2 (IGBMC Antibody facility), as mentioned on page 26 of the revised version of the manuscript. Since this is a "homemade" antibody, I would suggest including references in which this antibody has been characterized.

Referee #3 (Remarks for Author):

The revised manuscript is substantially improved, and I appreciate the author's clarifications.

I would still object to the closing statement of the abstract that this is a "highly specific" SCLC therapeutic. Lurbinectedin is inherently non-specific - it binds to CGC triplets throughout the genome, and thus suppresses thousands of target genes as the authors show well. Of course it suppresses ASCL1 and NEUROD1 targets in SCLC because these transcription factors are highly active in SCLC. I would strike "and highly specific."

Answers to the Editor:

Additionally, please also address the following editorial issues:

- Please address the queries from our data editors in track changes mode in the main manuscript file labelled **'Data edited MS file'**. Please use this file for any further modification (in track changes mode).

- Please provide up to 5 keywords

Transcription addiction; Lurbinectedin, ASCL1/NEUROD1; E-boxes/CpG islands.

- Javier Diez Perez is currently not entered in our submission system and is also missing from the authors' contribution. Please also double-check the discrepancy between Carlos Maria Genes (in the manuscript) and Carlos Mario Gene Robles (in the submission system).

Javier Diez Perez is now in your submission system and the authors' contribution.

Carlos Mario Gene Robles (in the submission system).

- Kindly rename the "References" section.

OK.

- Please upload the two EV tables as two separate files. Their legends should be removed from the manuscript and added to the respective file.

The two EV tables are in 2 separate files with the corresponding legends.

- Please include the Statistical Summary in the Appendix.

It is now included in the Appendix.

- Tables 1-4 should have their legends underneath the respective tables. The Appendix legends should be removed from the manuscript and moved to the appendix.

The Appendix legends have been removed and inserted below the respective Tables.

- Please carefully check that all figures and figure panels are referenced in the text. (Callouts are currently missing for Fig. EV3A, B).

These figures were referenced in the text, page 10.

- Kindly provide a synopsis image as an independent tiff, jpeg or PNG file 550 px wide x 300-600 px high.

This synopsis image is now included in the submitted manuscript.

- As part of the EMBO Publications transparent editorial process initiative (see our Editorial athttp://embomolmed.embopress.org/content/2/9/329), EMBO Molecular Medicine will publish online a Review Process File (RPF) to accompany accepted manuscripts.

This file will be published in conjunction with your paper and will include the anonymous referee reports, your point-by-point response and all pertinent correspondence relating to the manuscript. Let us know whether you agree with the publication of the RPF and as here, if you want to remove or not any figures from it prior to publication.

We will follow the EMBOMM rules.

Please note that the Authors checklist will be published at the end of the RPF.

***** Reviewer's comments *****

Referee #2 (Remarks for Author):

Peer review of the manuscript with the number EMM-2021-14841-V2, which is a revised version of the manuscript with the number EMM-2021-14841, by Costanzo F et. al. at EMBO Mol Med and with the tittle " Promoters of ASCL1- and NEUROD1-dependent genes are specific targets of lurbinectedin in SCLC cells

1). General description of the manuscript

For the general description of the work presented by Costanzo F et. al., as well as for the relevance of the contribution to scientific field, I would like to refer to the text of my peer review of the previous version of the manuscript.

Regarding the revised version of the manuscript, I do recognize that the current version of the manuscript have improved significantly. **The authors have tried to answer most of the concerns** of the reviewers, and have included some additional experiments. However, critical aspects have not been addressed, as I will explain in the following points.

2. Major Concerns
2.1. Data availability
As I have previously written, the model presented by Costanzo F et. al. mainly relies on interpretation of data that were generated by NGS-techniques. I clearly suggested to the authors to deposit raw data (FASTAQ files) of each the NGS experiments (RNA-seq, ChIP-seq, ATAC-seq, Chem-seq and BrdUTP ChIP-seq) in a data depository, for example NCBI's Gene Expression Omnibus, and provide accession numbers, with which the Reviewers could access the data during the peer review process to determine the quality of the data and reproduce the data analysis.
First, the authors provided in the point-by-point response to the reviewers an accession number and a password that did not contain the data from all the NGS experiments that were referred to in the manuscript. The data from the Chem-seq and BrdUTP ChIP-seq were not inside of this first accession number. Upon request, the authors provided a second accession number and the password with the information for Chem-seq and BrdUTP ChIP-seq.
Second, whereas the authors write in the point-by-point response to the reviewers that they uploaded the raw data into the data depository as follows: "We have now uploaded the FASTQ files corresponding to ChIP-seq, Chem-seq and RNA-seq data shown in the manuscript, "Data Availability: on Gene Expression Omnibus database (GSE179074)."
the authors write on page 29 of the revised version of the manuscript: "Sequence and processed data had been submitted to National Center for Biotechnology Gene Expression Omnibus with GEO accession number GSE179074." Sequence is not equal to FASTAQ file.
Third, after checking both accession numbers provided by the authors, I only found processed data. I did not find the FASTAQ files.
Even though nowadays the guidelines of recognized scientific journals, including EMBO Mol Med, clearly state that the access to NGS data should be granted by depositing the raw data in specific data depositories, this was not the case for the previous version AND for the current revised version of the manuscript presented by Costanzo et al. Therefore, I was not able to determine the quality of the NGS data generated by the authors, even worse, I was not able to reproduce the NGS data analysis to confirm that the data were analyzed appropriately. Due to the structure of the manuscript, the interpretation of the results and the conclusion made by the authors, the access to the raw NGS data is essential to be able to perform a complete and thought-out peer review.
This is a critical aspect. Just to clarify to the Editors, I would like to make a comparison: It is as one peer reviewer ask for the original pictures of microscopy pictures OR the original picture of a Western Blot without any processing, and the authors do not provide it.
Could you make an answer to this point?
We apologize for such concerns that disturbed the reviewing of referee 2. But we were not aware of the limitations of GEO when the projects are marked as "in private status" while the paper is on review. We sincerely believed that the reviewer had access to all the data we provided. In fact, we next understood that the link between GEO sample number and the corresponding SRA file number could not be available as far as the project is not released to public access.
In addition, we got from the SRA the following answer (see below the mail.): *"For unreleased data access to raw data is not possible. The only way is to release the data.* ***Usually, metadata is sufficient for reviewers****". For changing the release date or for making other updates please refer to the Update guide: https://www.ncbi.nlm.nih.gov/sra/docs/submitupdate/. To review your data or to generate reviewer's link to your unreleased data log in to Submission Portal's 'DataView' Interface: https://dataview.ncbi.nlm.nih.gov/*"""
Accordingly, the FASTQ files are now fully accessible. In GEO database (GSE179074<https://www.ncbi.nlm.nih.gov/geo/query/acc.cgi?acc=GSE179074> and GSE195663 https://www.ncbi.nlm.nih.gov/geo/query/acc.cgi?acc=GSE195663) the Reviewer can find processed data and the link to SRA browser (RUN selector) with the query to raw (FASTQ) files. In any case we enclose here the direct link and the accession code for the FASTQ files.

The accession code GSE195663 refers to the RNA-seq data used in figure 5F as well as EV3 and will be mentioned at the end of the Method section/Data availability of the manuscript together with the accession code GSE179074 corresponding to the rest of the data published.
The accession code GSE195663 contains the files:
GSM5844733 DMS53 SiCONTROL UNTREATED rep1
GSM5844734 DMS53 SiCONTROL UNTREATED rep2
GSM5844737 DMS53 SiCONTROL CISPT 20uM rep1
GSM5844738 DMS53 SiCONTROL CISPT 20uM rep2
GSM5844739 DMS53 SiCONTROL CISPT 50nM rep1
GSM5844740 DMS53 SiCONTROL CISPT 50nM rep2
Used for Figure 5F.
As well as:
GSM5844757 DMS53 SiA+N UNTREATED rep1
GSM5844758 DMS53 SiA+N UNTREATED rep2
Used in Figure EV3
For quick access to the raw data files we enclose the direct link to the SRA RUN selector containing the FASTQ files.

-. For GSE179074: https://www.ncbi.nlm.nih.gov/Traces/study/?acc=PRJNA742056&o=acc_s%3Aa
Accession code: PRJNA742056; each sample has its associated FASTQ file.
-. For GSE195663 https://www.ncbi.nlm.nih.gov/Traces/study/?acc=%09PRJNA801679&o=acc_s%3Aa
Accession code: PRJNA801679; each sample has its associated FASTQ file.

We checked and rechecked and we think that all the NGS experiments data are now accessible through the "Data Availability" section of the manuscript. We hope everything requested by referee is now completely available. We deeply apologize also due to some misunderstanding and also our fault.

The original pictures of microscopy pictures and Western Blots have been uploaded during the submission process and can be found in the Excel file entitled "Source data files".

2.2 Specificity of the effects of Lurbinectedin on ASCL1- and NEUROD1-dependent promoters in SCLC cells
Based on the previous point 2.1 and on the results presented in the manuscript, specially the new results presented on the Figure 4A, where the authors show a Western Blot analyzing the total levels of DNA-dependent RNA Polymerase II (Pol II, it is difficult to sustain their claims related to the specificity of the effects of Lurbinectedin on ASCL1- and NEUROD1-dependent promoters in SCLC cells.
I observe on the Figure 4A that Lurbinectedin reduced the levels of total Pol II in almost all of the cell lines analyzed. These results point into the direction of a global effect of Lurbinectedin on total levels of Transcription, despite of ASCL1- and NEUROD1-dependent promoters. I think that reviewer 3 also commented on this specific point in the previous version of the manuscript.
Indeed, referee 3 also commented on this specific point and he appreciated our clarification as seen below. We agree with reviewer 2 with her/his comment and we should moderate and make clearer our explanations. A considerably high amount of Lurbinectedin, specifically target CGG rich motifs (mainly found within CpG islands) in the promoter area (Figure EV2A). These motifs are accessible when chromatin is opened, which is the case when genes are activated. Such scenario might occur in any kind of cells when transcription is ongoing! However, as previously answered to referee 3 (*and agreed, see below*) "ASCL1 and NEUROD1 even more than other TFs, have pioneering activity, (Ireland, Micinski et al., 2020, Rudin et al., 2019) e.g. These transcription factors (overexpressed in some SCLC cells), **specifically and continuously** bind their E-box cognate sequences which are found

in a large number of transcribed genes (around 60% of gene promoters, – refer to Figure EV1 A for binding motifs and Table 1 column 2 (untreated, t=0) for ASCL1 and NEUROD1 bound genes). The overexpressed ASCL1 and NEUROD1 **continuously activate their dependent** genes, making them completely available to Lurbinectedin, that in turn primed the arrest of Pol II elongation and its further degradation. As a result, upon Lurbinectedin treatment, the expression of the ASCL1/NEUROD1 dependent genes (a part of which is crucial for cancer cells expansion and tumorigenesis), was abrogated. Moreover, we also have shown that the *ASCL1* and *NEUROD1* genes are found rapidly abrogated by lurbinectedin (Table 3).

Moreover, according to RNA-seq data, in normal conditions, one third of the genome are targeted by either ASCL1 (8131) and/or NEUROD1 (7329) and around 40% of transcribed genes were targeted either by ASCL1 or NEUROD1 (Figure 1B-C), pointing out their significant contribution to the transcription program of the SCLC cells (transcription addiction) as well as a certain degree of specificity.

In other cancer cell lines, little is known on the mechanism of their transcription addiction program. What is the mechanism behind gene activation in these cell lines? How is the expression of these genes regulated? These are still unanswered questions. If not well characterized, the same statement holds true for cell lines used just as a control. Lurbinectedin indeed can target genes being transcribed (likely at CGG rich motifs) in different cell lines and prime, as a consequence, degradation of RNAPII.

For the other cell lines shown in Figure 4a, nothing is also known about the relationship between Lurbinectedin, Pol II degradation and the nature of the abrogated genes. Even though, this represents definitely a point of interest and of further studies, it is still away from the objective of the manuscript. In fact, we show that Lurbinectedin efficiently target ASCL1 and NEUROD1 dependent genes in SCLC cell lines at CGG rich motifs when the genes are in an open and active state. Given that in SCLC cell lines these genes are constantly kept active, Lurbinectedin is the first in line in promptly stopping their expression and exhibit a certain specificity. We will moderate and make clearer our writing throughout the manuscript.

Another point that is important to mention: the authors implemented for the analysis of total levels of Pol II a Mouse anti-RNAPII 7c2 (IGBMC Antibody facility), as mentioned on page 26 of the revised version of the manuscript. Since this is a "homemade" antibody, I would suggest including references in which this antibody has been characterized.

This homemade antibody 7c2 was rather well characterized and has been extensively used by us as well as by other research teams. We here enclose studies in which the antibody has been utilized. We will include one of them in the manuscript.

- -. Kanagaraj, Radhakrishnan et al. "RECQ5 helicase associates with the C-terminal repeat domain of RNA polymerase II during productive elongation phase of transcription." *Nucleic acids research* vol. 38,22 (2010): 8131-40. doi:10.1093/nar/gkq697
- -. Santamaría Nuñez G, Robles CM, Giraudon C, Martínez-Leal JF, Compe E, Coin F, Aviles P, Galmarini CM, Egly JM. Lurbinectedin Specifically Triggers the Degradation of Phosphorylated RNA Polymerase II and the Formation of DNA Breaks in Cancer Cells. Mol Cancer Ther. 2016 Oct;15(10):2399-2412. doi: 10.1158/1535-7163.MCT-16-0172. Epub 2016 Sep 14. PMID: 27630271.
- -. Le May N, Mota-Fernandes D, Vélez-Cruz R, Iltis I, Biard D, Egly JM. NER factors are recruited to active promoters and facilitate chromatin modification for transcription in the absence of exogenous genotoxic attack. Mol Cell. 2010 Apr 9;38(1):54-66. doi: 10.1016/j.molcel.2010.03.004. PMID: 20385089.

Below, we also copy our previous answer to referee 3 concerning "The global effect of Lurbinectedin on total levels of Transcription" **which appreciated the author's clarifications".**

- *We understand the referee concerns and apologize for having been unclear. We should point out that ASCL1 and NEUROD1 even more than other TFs, have pioneering activity, (Ireland, Micinski*

*et al., 2020, Rudin et al., 2019) e.g. they activate a large number of genes upon binding to their E-box cognate sequence. Such binding is followed by a chromatin modification as shown by ATAC and H3K27ac signatures (**Figure 2C-D and Table 1**), thus allowing the formation of the preinitiation complex and further the reading of the gene by RNAPII. The opening of the chromatin represents an opportunity for lurbinectedin which might target the DNA at some CGG rich motifs located at the CpG islands.*

- *Moreover, RNA-seq show that a huge number of (6785) genes were down regulated, 4h after 50nM lurbinectedin treatment of DMS53 cells (**Table 2**). As mentioned by the referee, "the transcriptional program of SCLC lines is indeed highly dependent on expressed ASCL1 and/or NEUROD1 transcriptional regulators. Among them, 2194 were targeted by 50nM lurbinectedin 4h post treatment and downregulated **(Table 3):** 76% (1672) of them are ASCL1 and/or NEUROD1 targeted demonstrating a certain degree of specificity; the others are likely genes either regulated by the proteins produced by the ASCL1/NEUROD1 dependent genes or housekeeping genes being likely in an open chromatin conformation.*

Referee #3 (Remarks for Author):
The revised manuscript is substantially improved, and **I appreciate the author's clarifications**. I would still object to the closing statement of the abstract that this is a "highly specific" SCLC therapeutic. Lurbinectedin is inherently non-specific - it binds to CGC triplets throughout the genome, and thus suppresses thousands of target genes as the authors show well. Of course, it suppresses ASCL1 and NEUROD1 targets in SCLC because these transcription factors are highly active in SCLC. I would strike "and highly specific."
We deeply thank this referee who appreciates our clarification. We followed his suggestions to moderate our conclusions. We agree and eliminated it throughout the text, accordingly.

15th Feb 2022

Dear Prof. Egly,

Thank you for submitting your revised manuscript to EMBO Molecular Medicine.
We have now received the report from referee #3, and we will be able to accept your manuscript once the following editorial issues will be addressed:

1/ Manuscript text:
- Please accept the changes and remove the text in blue.
- In the material and methods, please indicate whether the cells were authenticated (when applicable) and tested for mycoplasma contamination, and kindly provide the dilutions used for the antibodies.
- Please remove the synopsis from the main text and upload it separately.

2/ Checklist:
Please indicate N/A in all sections related to animal models and human subjects.
Please update the Data Accessibility section.
Please fill in section G, Dual use research of concern.

3/ Competing interests:
Please remove "Data and materials availability: [...] Reprints and permission information is available online at ....".
Moreover, editorial board members, EMBO council members, EMBO publications advisory board members, and EMBO staff must disclose their relationship with EMBO in the author disclosure statement using the standard phrase: "[Author] is an editorial advisory board/EMBO Member. This has no bearing on the editorial consideration of this article for publication."

4/ Thank you for providing the Paper Explained. I slightly edited it, please let me know if you agree with the following, or amend as you see fit:
Problem
SCLC is an extremely aggressive cancer, characterized by rapid growth, early metastasis, and refractoriness to current therapies. In contrast to NSCLC, SCLC did not benefit from significant therapeutic advances over the last decades. We explored a new therapeutic strategy based on a better understanding of SCLC development, and particularly on its addiction to transcription.
Results
We found that the SCLC transcription addiction was mainly directed by activators such as ASCL1 and NEUROD1, who continuously stimulated the expression of their target genes. These genes, constantly in an open chromatin environment, were susceptible to lurbinectedin, a marine alkaloid that specifically targets G rich DNA triplets in CpG island motifs located downstream of the gene transcription start site. Accordingly, lurbinectedin was used to block ASCL1 and NEUROD1 dependent transcription programs, and thus trigger apoptotic programs in a panel of SCLC cell lines.
Impact
This study demonstrates how targeting specific areas of the genome results in a more effective and less harmful anti-tumor effect. Our data clarify the mechanism of action of ASCL1 and NEUROD1, revealing their genomic targets in different genomic contexts, which represents a step forward for personalized medicine in SCLC patients.

5/ Synopsis:
I modified your text to fit our style and format (mostly by shortening it), please let me know if you agree with the following or amend as you see fit:
SCLCs poorly respond to current treatments, underlying the need for new therapeutic strategies. This study reports how the DNA binder lurbinectedin benefits from the SCLC cells transcription addiction to block the transcriptional program of key transcription factors involved in disease progression.

- ASCL1 and NEUROD1 target their E-box cognate sequence and control the expression of a large number of genes in SCLC.
- Lurbinectedin, a marine alkaloid, targets the central G rich triplets found in CpG islands mainly located downstream from the genes transcription start site.
- Lurbinectedin binding abolishes the expression of ASCL1 and NEUROD1 target genes, such as INSM1, MYC, MYB and BCL2.
- Blocking ASCL1 and NEUROD1 transcriptional program drives SCLC cells towards programmed cell death and deeply impacts tumorigenesis.

Thank you for providing a nice synopsis picture, please resize to 550 px wide x 300-600 px high.

Thank you for bearing with these last changes, I look forward to receiving your revised manuscript at your earliest convenience.

With kind regards,

Lise

Lise Roth, PhD
Editor
EMBO Molecular Medicine

To submit your manuscript, please follow this link:

Link Not Available

***** Reviewer's comments *****

Referee #3 (Comments on Novelty/Model System for Author):

This is a mechanistic paper, it does not have high impact on any medical decision making, but seeks to provide insight into how this novel alkylating agent is affecting small cell lung cancer through affecting transcription.

Referee #3 (Remarks for Author):

I believe that concerns of reviewers #1 and #2 have been adequately addressed by the authors. Reviewer #3 in the most recent iteration had additional concerns that are beyond the scope of expertise of this particular reviewer.

The authors performed the requested changes.

17th Feb 2022

Dear Prof. Egly,

Thank you for addressing our last editorial queries. I am pleased to inform you that your manuscript is accepted for publication and is now being sent to our publisher to be included in the next available issue of EMBO Molecular Medicine!

Please read below for additional IMPORTANT information regarding your article, its publication and the production process.

Congratulations on your interesting work,

With my best wishes,

Lise

Lise Roth, Ph.D
Scientific Editor
EMBO Molecular Medicine

Follow us on Twitter @EmboMolMed
Sign up for eTOCs at embopress.org/alertsfeeds

EMBO PRESS

YOU MUST COMPLETE ALL CELLS WITH A PINK BACKGROUND ➔

PLEASE NOTE THAT THIS CHECKLIST WILL BE PUBLISHED ALONGSIDE YOUR PAPER

Corresponding Author Name: Jean Marc Egly
Journal Submitted to: EMBO molecular medicine
Manuscript Number: EMM-2021-14841

USEFUL LINKS FOR COMPLETING THIS FORM

http://www.antibodypedia.com
http://1degreebio.org
http://www.equator-network.org/reporting-guidelines/improving-bioscience-research-repor
http://grants.nih.gov/grants/olaw/olaw.htm
http://www.mrc.ac.uk/Ourresearch/Ethicsresearchguidance/Useofanimals/index.htm
http://ClinicalTrials.gov
http://www.consort-statement.org
http://www.consort-statement.org/checklists/view/32-consort/66-title
http://www.equator-network.org/reporting-guidelines/reporting-recommendations-for-tumo
http://datadryad.org
http://figshare.com
http://www.ncbi.nlm.nih.gov/gap
http://www.ebi.ac.uk/ega
http://biomodels.net/
http://biomodels.net/miriam/
http://jjj.biochem.sun.ac.za
https://osp.od.nih.gov/biosafety-biosecurity-and-emerging-biotechnology/
http://www.selectagents.gov/

Reporting Checklist For Life Sciences Articles (Rev. June 2017)

This checklist is used to ensure good reporting standards and to improve the reproducibility of published results. These guidelines are consistent with the Principles and Guidelines for Reporting Preclinical Research issued by the NIH in 2014. Please follow the journal's authorship guidelines in preparing your manuscript.

A- Figures
1. Data
The data shown in figures should satisfy the following conditions:

- the data were obtained and processed according to the field's best practice and are presented to reflect the results of the experiments in an accurate and unbiased manner.
- figure panels include only data points, measurements or observations that can be compared to each other in a scientifically meaningful way.
- graphs include clearly labeled error bars for independent experiments and sample sizes. Unless justified, error bars should not be shown for technical replicates.
- if n< 5, the individual data points from each experiment should be plotted and any statistical test employed should be justified
- Source Data should be included to report the data underlying graphs. Please follow the guidelines set out in the author ship guidelines on Data Presentation.

2. Captions

Each figure caption should contain the following information, for each panel where they are relevant:

- a specification of the experimental system investigated (eg cell line, species name).
- the assay(s) and method(s) used to carry out the reported observations and measurements
- an explicit mention of the biological and chemical entity(ies) that are being measured.
- an explicit mention of the biological and chemical entity(ies) that are altered/varied/perturbed in a controlled manner.

- the exact sample size (n) for each experimental group/condition, given as a number, not a range;
- a description of the sample collection allowing the reader to understand whether the samples represent technical or biological replicates (including how many animals, litters, cultures, etc.).
- a statement of how many times the experiment shown was independently replicated in the laboratory.
- definitions of statistical methods and measures:
  - common tests, such as t-test (please specify whether paired vs. unpaired), simple $\chi 2$ tests, Wilcoxon and Mann-Whitney tests, can be unambiguously identified by name only, but more complex techniques should be described in the methods section;
  - are tests one-sided or two-sided?
  - are there adjustments for multiple comparisons?
  - exact statistical test results, e.g., P values = x but not P values < x;
  - definition of 'center values' as median or average;
  - definition of error bars as s.d. or s.e.m.

Any descriptions too long for the figure legend should be included in the methods section and/or with the source data.

In the pink boxes below, please ensure that the answers to the following questions are reported in the manuscript itself. Every question should be answered. If the question is not relevant to your research, please write NA (non applicable). We encourage you to include a specific subsection in the methods section for statistics, reagents, animal models and human subjects.

B- Statistics and general methods

Please fill out these boxes ➔ (Do not worry if you cannot see all your text once you press return)

1.a. How was the sample size chosen to ensure adequate power to detect a pre-specified effect size?	Sample size for NGS studies were stablished following guidelines for NGS experiment design. For in vitro experiments, sample size of at least 3 indipendent experiments was chosen in order to give enough statistical strenght to the data.
1.b. For animal studies, include a statement about sample size estimate even if no statistical methods were used.	NA
2. Describe inclusion/exclusion criteria if samples or animals were excluded from the analysis. Were the criteria pre-established?	All samples generated were used into analysis
3. Were any steps taken to minimize the effects of subjective bias when allocating animals/samples to treatment (e.g. randomization procedure)? If yes, please describe.	No
For animal studies, include a statement about randomization even if no randomization was used.	NA
4.a. Were any steps taken to minimize the effects of subjective bias during group allocation or/and when assessing results (e.g. blinding of the investigator)? If yes please describe.	No
4.b. For animal studies, include a statement about blinding even if no blinding was done	NA
5. For every figure, are statistical tests justified as appropriate?	Yes
Do the data meet the assumptions of the tests (e.g., normal distribution)? Describe any methods used to assess it.	NGS statisticals analysis performed includes assumtion testing in the model used. Deseq2 uses estimate variance-mean dependence in count data from high-throughput sequencing assays and test for differential expression based on a model using the negative binomial distribution, that is tested before performing the analysis using this model. For data analyzed with t-test or 2-way ANOVA data normality has been analyzed through Shapiro-Wilk (W) test.
Is there an estimate of variation within each group of data?	All F tests corresponding to the t-test has been assessed and provided with the manuscript.

Is the variance similar between the groups that are being statistically compared?	Yes

C- Reagents

6. To show that antibodies were profiled for use in the system under study (assay and species), provide a citation, catalog number and/or clone number, supplementary information or reference to an antibody validation profile. e.g., Antibodypedia (see link list at top right), 1DegreeBio (see link list at top right).	Immunofluorescence, Western Blots and ChIP has been performed on Humam derived cell lines with the following antibody list : -Recombinant Anti-MASH1/Achaete-scute homolog 1 antibody [EPR19840] (ab211327) DOI: 10.1101/gad.328336.119. Recombinant Anti-NeuroD1 antibody [EPR20766] (ab213725) DOI: 10.3389/fendo.2019.00735. RNAPII RPB1 7c2 antibody (IGBMC antibody facility) DOI: 10.1038/s41467-019-10131-1. Anti-RNA polymerase II CTD repeat YSPTSPS (phospho S2) antibody (ab5095) DOI: 10.1002/1878-0261.12906. Anti-Ubiquitin Antibody (P4D1): sc-8017 DOI: 10.1038/ncb2522. Anti-Biotin antibody (ab53494) DOI: 10.1002/ctm2.373. Histone H3K27ac antibody (pAb) DOI: 10.1038/nsmb.2274. Anti-gamma H2A.X (phospho S139) antibody (ab2893). DOI: 10.1016/j.cell.2020.11.042.
7. Identify the source of cell lines and report if they were recently authenticated (e.g., by STR profiling) and tested for mycoplasma contamination.	All cell lines have been obtained from ATCC and regularly tested for Mycoplasm contramination through activity of mycoplasmal enzymes test.

* for all hyperlinks, please see the table at the top right of the document

D- Animal Models

8. Report species, strain, gender, age of animals and genetic modification status where applicable. Please detail housing and husbandry conditions and the source of animals.	NA
9. For experiments involving live vertebrates, include a statement of compliance with ethical regulations and identify the committee(s) approving the experiments.	NA
10. We recommend consulting the ARRIVE guidelines (see link list at top right) (PLoS Biol. 8(6), e1000412, 2010) to ensure that other relevant aspects of animal studies are adequately reported. See author guidelines, under 'Reporting Guidelines'. See also: NIH (see link list at top right) and MRC (see link list at top right) recommendations. Please confirm compliance.	NA

E- Human Subjects

11. Identify the committee(s) approving the study protocol.	NA
12. Include a statement confirming that informed consent was obtained from all subjects and that the experiments conformed to the principles set out in the WMA Declaration of Helsinki and the Department of Health and Human Services Belmont Report.	NA
13. For publication of patient photos, include a statement confirming that consent to publish was obtained.	NA
14. Report any restrictions on the availability (and/or on the use) of human data or samples.	NA
15. Report the clinical trial registration number (at ClinicalTrials.gov or equivalent), where applicable.	NA
16. For phase II and III randomized controlled trials, please refer to the CONSORT flow diagram (see link list at top right) and submit the CONSORT checklist (see link list at top right) with your submission. See author guidelines, under 'Reporting Guidelines'. Please confirm you have submitted this list.	NA
17. For tumor marker prognostic studies, we recommend that you follow the REMARK reporting guidelines (see link list at top right). See author guidelines, under 'Reporting Guidelines'. Please confirm you have followed these guidelines.	NA

F- Data Accessibility

18: Provide a "Data Availability" section at the end of the Materials & Methods, listing the accession codes for data generated in this study and deposited in a public database (e.g. RNA-Seq data: Gene Expression Omnibus GSE39462, Proteomics data: PRIDE PXD000208 etc.) Please refer to our author guidelines for 'Data Deposition'.<br><br>Data deposition in a public repository is mandatory for:<br>a. Protein, DNA and RNA sequences<br>b. Macromolecular structures<br>c. Crystallographic data for small molecules<br>d. Functional genomics data<br>e. Proteomics and molecular interactions	All NGS data are available at Gene Expression Omnibus under GSE179074 (https://www.ncbi.nlm.nih.gov/geo/query/acc.cgi?acc=GSE179074) and GSE195663 (https://www.ncbi.nlm.nih.gov/geo/query/acc.cgi?acc= GSE195663)
19. Deposition is strongly recommended for any datasets that are central and integral to the study; please consider the journal's data policy. If no structured public repository exists for a given data type, we encourage the provision of datasets in the manuscript as a Supplementary Document (see author guidelines under 'Expanded View' or in unstructured repositories such as Dryad (see link list at top right) or Figshare (see link list at top right).	Yes
20. Access to human clinical and genomic datasets should be provided with as few restrictions as possible while respecting ethical obligations to the patients and relevant medical and legal issues. If practically possible and compatible with the individual consent agreement used in the study, such data should be deposited in one of the major public access-controlled repositories such as dbGAP (see link list at top right) or EGA (see link list at top right).	access through reviewer codes and and is scheduled to be released on Dec 31, 2021
21. Computational models that are central and integral to a study should be shared without restrictions and provided in a machine-readable form. The relevant accession numbers or links should be provided. When possible, standardized format (SBML, CellML) should be used instead of scripts (e.g. MATLAB). Authors are strongly encouraged to follow the MIRIAM guidelines (see link list at top right) and deposit their model in a public database such as Biomodels (see link list at top right) or JWS Online (see link list at top right). If computer source code is provided with the paper, it should be deposited in a public repository or included in supplementary information.	NA

G- Dual use research of concern

22. Could your study fall under dual use research restrictions? Please check biosecurity documents (see link list at top right) and list of select agents and toxins (APHIS/CDC) (see link list at top right). According to our biosecurity guidelines, provide a statement only if it could.	NA
